# Distinct epigenetic profiles in children with perinatally-acquired HIV on antiretroviral therapy

**DOI:** 10.1038/s41598-019-46930-1

**Published:** 2019-07-19

**Authors:** Stephanie Shiau, Renate Strehlau, Shuang Wang, Avy Violari, Catherine Do, Faeezah Patel, Afaaf Liberty, Izabela Krupska, Stephen M. Arpadi, Marc Foca, Ashraf Coovadia, Elaine J. Abrams, Benjamin Tycko, Mary Beth Terry, Louise Kuhn

**Affiliations:** 1 Gertrude H. Sergievsky Center, Vagelos College of Physicians and Surgeons, Columbia University Irving Medical Center, New York, NY 10032 USA; 2 Department of Epidemiology, Mailman School of Public Health, Columbia University Irving Medical Center, New York, NY 10032 USA; 3 Empilweni Services and Research Unit, Rahima Moosa Mother and Child Hospital, University of the Witwatersrand, Johannesburg, South Africa; 4 Department of Biostatistics, Mailman School of Public Health, Columbia University Irving Medical Center, New York, NY 10032 USA; 5 Perinatal HIV Research Unit, Chris Hani Baragwanath Hospital, University of the Witwatersrand, Johannesburg, South Africa; 6 Department of Biomedical Research, Division of Genetics & Epigenetics, Hackensack Meridian School of Medicine at Seton Hall and the Center for Innovation and Discovery, Nutley, NJ 07110 USA; 7 JP Sulzberger Columbia Genome Center, Columbia University Irving Medical Center, New York, 10032 NY USA; 8 Department of Pediatrics, Vagelos College of Physicians and Surgeons, Columbia University Irving Medical Center, New York, NY 10032 USA; 9 ICAP at Columbia University, New York, NY 10032 USA

**Keywords:** HIV infections, Molecular medicine, Infection

## Abstract

Perinatally-acquired HIV has persistent effects on long-term health outcomes, even after early treatment. We hypothesize that epigenetic indicators, such as DNA methylation, may elucidate cellular processes that explain these effects. Here, we compared DNA methylation profiles in whole blood from 120 HIV-infected children on antiretroviral therapy (ART) and 60 frequency age-matched HIV-uninfected children aged 4–9 years in Johannesburg, South Africa. Using an individual CpG site approach, we found 1,309 differentially-methylated (DM) CpG sites between groups, including 1,271 CpG sites that were hyper-methylated in the HIV-infected group and 38 CpG sites that were hypo-methylated in the HIV-infected group. Six hyper-methylated CpG sites were in *EBF4*, which codes for a transcription factor involved in B-cell maturation. The top hypomethylated site was in the promoter region of *NLRC5*, encoding a transcription factor that regulates major histocompatibility complex (MHC) class I molecule expression. Using a differentially-methylated region (DMR) approach, we found 315 DMRs between groups, including 28 regions encompassing 686 CpG sites on chromosome 6. A large number of the genes identified in both the CpG site and DMR approaches were located in the MHC region on chromosome 6, which plays an important role in the adaptive immune system. This study provides the first evidence that changes in the epigenome are detectable in children with perinatally-acquired HIV infection on suppressive ART started at an early age.

## Introduction

Despite the success of programs to prevent mother-to-child HIV transmission (MTCT) and improve accessibility of antiretroviral therapy (ART), an estimated 2.6 million children live with HIV globally, 90% of them in sub-Saharan Africa^1^. In the absence of a cure for HIV, this cohort will live with the burden of the disease and remain on lifelong ART as they grow into adolescence and adulthood. It is well-established that perinatally-acquired HIV has persistent effects on long-term health outcomes, even after early treatment. Reported morbidities include cognitive deficits, metabolic abnormalities and increased cardiovascular disease risk, bone loss, and renal complications^2–7^.

Perinatal or early life insults have been linked to altered epigenetic markers that contribute to disease manifestation^8–12^. Similarly, we hypothesized that epigenetic indicators, such as DNA methylation (DNAm), may elucidate cellular processes that explain the persistent effects of HIV on long-term health outcomes. DNAm is one of the most commonly studied epigenetic changes in humans and involves the addition of a methyl group to a cytosine followed by a guanine (cytosine-phosphate-guanine; CpG)^13^. These modifications can alter gene expression without altering the primary DNA sequence^14^.

Recent work has uncovered associations between HIV and DNAm in the host genome in adults. A study of 137 HIV-infected, ART-treated white non-Hispanic males (mean age 48 years) and 44 uninfected males identified 81,361 CpG sites associated with HIV infection, including decreased DNAm in the chromosomal region encoding the human leukocyte antigen (HLA) locus^15^. Another study of 261 HIV-infected (mean age 49 years, 96.9% male, 67% African-American, 79% on ART) and 117 HIV-uninfected adults identified 20 CpG sites associated with HIV infection, including 2 CpG sites in *NLRC5*, which codes for a key regulator of major histocompatibility complex (MHC) class I gene expression^16^. A third study of 19 ART-naïve adults with HIV (mean age 51.1 years) and 19 HIV-negative male participants found an association of HIV infection with one CpG site in *VPS37B*, encoding a regulator of viral budding and transport^17^. Although all three studies found evidence of differential DNAm associated with HIV, these studies were conducted in primarily adult male populations, and findings may be confounded by lifestyle and environmental factors expected to influence DNAm.

To make progress in this area, we compared DNAm profiles in ART-treated HIV-infected children and HIV-uninfected children in South Africa, a country bearing a large burden of the HIV epidemic^1^. Children with perinatally-acquired HIV represent a unique model population. There is compelling evidence that early life exposures influence epigenetics, and in contrast to adults, children are likely to have different epigenetic responses to HIV that shape their development during critical life periods. In addition, as many of the behavioral risk factors that may lead to epigenetic changes (e.g. smoking, physical inactivity, and drug use) are not predominant in early childhood, it is important to study epigenetic processes not only in adults, but also in children.

## Results

### Characteristics of the study participants

Demographic characteristics of the 119 HIV-infected and 60 uninfected South African children ages 4–9 years included in this study are in Table 1. All characteristics were similar between groups. The HIV-infected children had a median CD4 percentage of 33.1% and HIV RNA quantity <400 copies/mL at the time of measurement. ART was started at a mean (SD) age of 7.7 (6.1) months; by design 60 started ART <6 months of age and 59 started ART 6–24 months of age and children had been on ART an average of 5.8 years at the time of measurement. Characteristics of children started on ART <6 months and 6–24 months are also presented in Table 1. Those started on ART <6 months of age were younger than those started on ART 6–24 months at the time of DNAm measurement.

**Table 1:** Characteristics of 119 HIV-infected and 60 HIV-uninfected school-aged children in Johannesburg, South Africa; HIV-infected children are stratified by age at start of antiretroviral therapy (ART), <6 months vs. 6–24 months.

Characteristic	HIV-infected (N = 119)	HIV-uninfected (N = 60)	P	HIV-infected, started ART <6 months (N = 60)	HIV-infected, started ART 6-24 months (N = 59)	P
Male, N (%)	55 (46.2)	30 (50.0)	0.63	29 (48.3)	26 (44.1)	0.64
Age (years), Mean (SD)	6.42 (1.4)	6.43 (1.4)	0.97	6.05 (1.2)	6.80 (1.6)	0.004
Age (years), N (%)						
4–5	24 (20.0)	12 (20.0)	1.0	13 (21.7)	11 (18.7)	0.03
5–6	28 (23.3)	14 (23.3)		18 (30.0)	10 (17.0)	
6–7	26 (21.9)	13 (21.7)		16 (26.7)	10 (17.0)	
7–8	20 (16.8)	10 (16.7)		9 (15.0)	11 (17.0)	
8–9	19 (16.0)	10 (16.7)		4 (6.7)	15 (25.4)	
9–10	2 (1.7)	1 (1.7)		0 (0.0)	2 (3.4)	
Type of home, N (%)						
House	74 (62.2)	38 (63.3)	0.78	37 (61.7)	37 (62.7)	0.89
Flat	5 (4.2)	3 (5.0)		2 (3.3)	3 (5.1)	
Shack	23 (19.3)	8 (13.3)		13 (21.7)	10 (17.0)	
Outbuilding	1 (0.8)	0 (0.0)		0 (0.0)	1 (1.7)	
Rented room	16 (13.5)	11 (18.3)		8 (13.3)	8 (13.6)	
Household member smokes, N (%)	41 (34.5)	22 (36.7)	0.77	19 (31.7)	22 (37.3)	0.52
Primary cooking method, N (%)						
Paraffin burner	9 (7.6)	1 (1.7)	0.19	6 (10.0)	3 (5.1)	0.34
Electric cooker	106 (89.1)	55 (91.7)		51 (85.0)	55 (93.2)	
Gas stove	4 (3.4)	4 (6.7)		3 (5.0)	1 (1.7)	
Mother born in South Africa, N (%)	109 (91.6)	56 (93.3)	0.78	57 (95.0)	52 (88.1)	0.2
Mother primary caregiver, N (%)	108 (90.8)	57 (95.0)	0.62	53 (88.3)	55 (93.2)	0.32
Highest caregiver grade, N (%)						
No School	0 (0.0)	1 (1.7)	0.47	0 (0.0)	0 (0.0)	0.53
1–11 (Primary/Secondary)	66 (55.5)	36 (60.0)		35 (58.3)	31 (52.5)	
12 (finished high school)	53 (44.2)	23 (38.3)		25 (41.7)	28 (47.5)	
Caregiver has paid job, N (%)	52 (43.7)	22 (36.7)	0.37	25 (41.7)	27 (45.8)	0.65
Caregiver marital status, N (%)						
Single	86 (72.3)	43 (71.7)	0.21	44 (73.3)	42 (71.2)	0.51
Married	25 (21.0)	17 (28.3)		11 (18.3)	14 (23.7)	
Divorced/Widowed	5 (4.2)	0 (0.0)		4 (6.7)	1 (1.7)	
Other	3 (2.5)	0 (0.0)		1 (1.7)	2 (3.4)	
Pre-treatment CD4 percentage, Mean (SD)	25.2 (11.1)	—	—	27.3 (10.6)	22.9 (11.3)	0.04
Missing	7			1	6	
Pre-treatment CD4 count (cells/mm^3^), Mean (SD)	1401 (927)	—	—	1661 (936)	1112 (834)	0.002
Missing	9			2	7	
Pre-treatment HIV RNA quantity (copies/mL), N (%)						
<100,000	7 (7.2)			3 (5.8)	4 (8.9)	0.91
100,000–340,000	20 (20.6)			10 (19.2)	10 (22.2)	
340,000–750,000	13 (13.4)	—	—	7 (13.5)	6 (13.3)	
≥750,000	57 (58.8)			32 (61.5)	25 (55.6)	
Missing	22			8	14	
Age at ART start (months), Mean (SD)	7.7 (6.1)	—	—	3.2 (1.3)	12.3 (5.5)	<0.001
Enrollment CD4 percentage, Mean (SD)	33.4 (6.4)	—	—	32.5 (6.9)	34.4 (5.8)	0.11
Missing	3			2	1	
Enrollment CD4 count (cells/mm^3^), Mean (SD)	1181 (476)	—	—	1159 (511)	1203 (442)	0.62
Missing	3			2	1	
Enrollment HIV RNA quantity (copies/mL), N (%)						
LDL	96 (80.7)			50 (83.3)	46 (78.0)	0.49
<40	16 (13.5)	—	—	8 (13.3)	8 (13.6)	
40–400	7 (5.9)			2 (3.3)	5 (8.5)	
>400	0 (0.0)			0 (0.0)	0 (0.0)	

### Genome-wide analysis of CpG sites with differential methylation associated with HIV

Using the 370,683 probes considered suitable for analysis, we first conducted an unadjusted analysis to test for DNAm differences at individual CpG sites between HIV-infected and uninfected children. A total of 1,639 CpG sites showed significant differential methylation between groups with an FDR q-value < 0.05 and |Δβ| > 0.05. In an analysis adjusted for sex, age, and cell type proportion, we found 1,309 DM CpG sites between groups (Table S1). There was an overlap of 1,282 CpG sites between the 1,639 in the unadjusted analysis and 1,309 in the adjusted analysis.

**Figure 1:**
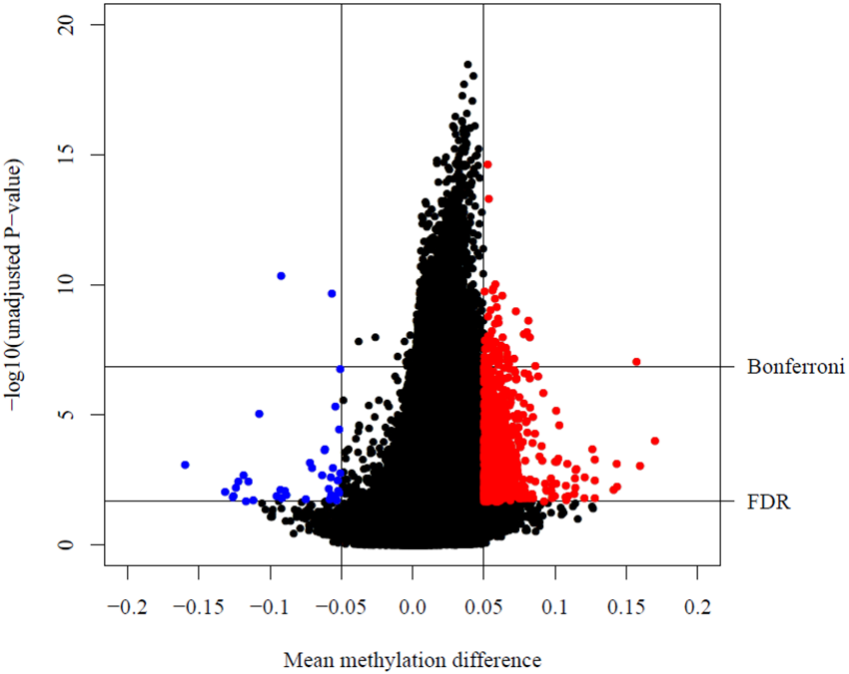
Volcano plot displays mean DNA methylation difference between HIV-infected and HIV-uninfected groups at genome-wide CpG sites vs. −log10(unadjusted P-value) after adjustment for age, sex, and cell type proportion. Selected differentially methylated CpG sites (N = 1,309) with FDR q-value < 0.05 and |Δβ| > 0.05 are presented in blue (hypo-methylated in HIV-infected group) and red (hyper-methylated in HIV-infected group). FDR line is drawn at −log10(0.02123351); Bonferroni line is drawn at −log10(1.35e-07).

Figure 1 shows a volcano plot of the adjusted results. Strikingly, 1,271 of the 1,309 DM sites, shown in red, were hyper-methylated in the HIV-infected group compared to the HIV-uninfected group while only 38 sites, shown in blue, were hypo-methylated. Overall, the DM CpGs were enriched in “open sea” locations [57.1% of the DM sites; odds ratio (OR) = 2.34, p-value < 0.01, relative to the genome-wide average], and relatively depleted (6.3% of the DM sites, OR = 0.15, p-value < 0.0) in CpG island locations (Fig. S1).

Out of 1,309 selected sites, 69 CpG sites located in 47 known genes met a more stringent Bonferroni threshold (p < 1.35 × 10^−7^) (Table 2). The top 2 sites (cg09239591, cg23101680) by significance level were located in the promoter region (TSS1500) of *XDH* and gene body of *SPERT*, respectively. 67 of 69 sites were hyper-methylated in the HIV-infected group compared to the HIV-uninfected group while 2 sites were hypo-methylated (top left quadrant of the plot in Fig. 1). Four of the top 69 significant CpG sites (cg17069396, cg16426670, cg05825244, and cg24263062) on chromosome 20 were located in the gene body of *EBF4*. All 4 were hyper-methylated in HIV-infected children. One of the 4 CpG sites (cpg05825244) had the largest |Δβ| (0.157) of the 69 sites meeting the Bonferroni threshold. The two hypo-methylated sites (cg07839457, cg13316433) were among the top 8 overall sites by significance level. The first site was located in the promoter region (TSS1500) of *NLRC5*. Gene information for the second CpG site was unknown.

**Table 2:** Top selected differentially methylated CpG sites (N = 69) associated with HIV after adjustment for age, sex, and cell type proportion meeting Bonferroni threshold (p < 1.35 × 10 − 7) and with |Δβ| > 0.05, sorted by P-value from smallest to largest.

CpG	CHR	Position	Gene	Relation to Gene	Relation to CpG Island	β (HIV+)	β (HIV−)	Δβ	P	Adj P
cg09239591	2	31638511	XDH	TSS1500	OpenSea	0.675	0.623	0.053	2.44E-15	1.97E-11
cg23101680	13	46277286	SPERT	Body	OpenSea	0.717	0.663	0.053	4.89E-14	1.83E-10
cg07839457	16	57023022	NLRC5	TSS1500	N_Shore	0.261	0.353	−0.092	4.69E-11	2.87E-08
cg12027254	17	76055290	TNRC6C	Body;Body	OpenSea	0.824	0.766	0.058	1.00E-10	4.99E-08
cg07970325	6	106497542			OpenSea	0.569	0.512	0.057	1.39E-10	6.38E-08
cg26280998	6	53589544			OpenSea	0.715	0.659	0.056	1.72E-10	7.29E-08
cg12880685	10	120489658	C10orf46	Body	OpenSea	0.428	0.378	0.050	1.85E-10	7.66E-08
cg13316433	5	83140500			OpenSea	0.150	0.207	−0.057	2.28E-10	8.94E-08
cg00390724	3	18484742			N_Shore	0.519	0.456	0.063	2.66E-10	1.01E-07
cg22690339	6	38249061	BTBD9	Body	OpenSea	0.792	0.735	0.058	3.47E-10	1.22E-07
cg05929755	1	110663559			OpenSea	0.398	0.339	0.059	7.36E-10	2.15E-07
cg12021671	15	42423807			OpenSea	0.744	0.690	0.054	1.02E-09	2.75E-07
cg08237220	3	156332967			OpenSea	0.531	0.459	0.072	1.07E-09	2.85E-07
cg05006384	14	95942319	C14orf49	TSS200	OpenSea	0.648	0.595	0.053	1.74E-09	4.09E-07
cg13707760	9	95986383	WNK2	Body	OpenSea	0.509	0.449	0.060	2.03E-09	4.58E-07
cg18394552	5	159428643			OpenSea	0.732	0.650	0.081	2.42E-09	5.17E-07
cg10336193	1	206848389			OpenSea	0.644	0.584	0.060	2.88E-09	5.88E-07
cg22348290	8	72459499			N_Shore	0.767	0.709	0.058	3.22E-09	6.37E-07
cg26898099	5	95192949	C5orf27	Body	OpenSea	0.706	0.650	0.055	6.44E-09	1.07E-06
cg14418857	7	121120431			OpenSea	0.616	0.535	0.080	6.86E-09	1.13E-06
cg22647738	17	34304462	CCL16	3′UTR	OpenSea	0.639	0.561	0.078	8.18E-09	1.29E-06
cg05512157	12	50901878	DIP2B	Body	S_Shelf	0.587	0.533	0.054	8.71E-09	1.35E-06
cg21912162	5	71447818	MAP1B	Body	OpenSea	0.329	0.276	0.053	9.65E-09	1.46E-06
cg23720384	21	23747055			OpenSea	0.665	0.583	0.082	1.10E-08	1.61E-06
cg04961225	12	91332479			OpenSea	0.578	0.515	0.063	1.12E-08	1.63E-06
cg10689689	11	63677197	MARK2	3′UTR	S_Shore	0.685	0.634	0.051	1.42E-08	1.93E-06
cg15826519	1	19808575	CAPZB	Body	N_Shelf	0.796	0.742	0.054	1.49E-08	2.00E-06
cg09083958	12	131516365	GPR133	Body	N_Shore	0.696	0.642	0.054	1.53E-08	2.04E-06
cg02341197	21	34185927	C21orf62	1stExon;5′UTR	OpenSea	0.466	0.408	0.058	1.54E-08	2.05E-06
cg08937102	1	1690389	NADK	Body	N_Shore	0.481	0.428	0.053	1.75E-08	2.24E-06
cg20954180	3	54606265	CACNA2D3	Body	OpenSea	0.847	0.794	0.053	1.75E-08	2.25E-06
cg17069396	20	2731102	EBF4	Body	Island	0.128	0.069	0.058	1.81E-08	2.30E-06
cg16426670	20	2675996	EBF4	Body	S_Shore	0.812	0.750	0.062	2.29E-08	2.74E-06
cg15773890	6	17259549			OpenSea	0.866	0.812	0.054	2.73E-08	3.12E-06
cg03705319	12	12848516	GPR19	5′UTR	N_Shore	0.745	0.693	0.052	2.75E-08	3.14E-06
cg00602295	16	81328089			OpenSea	0.441	0.375	0.065	2.81E-08	3.18E-06
cg17009978	3	183959000	VWA5B2;MIR1224	Body;TSS200	N_Shore	0.719	0.667	0.052	2.84E-08	3.21E-06
cg24399529	15	69373153	MIR548H4;TMEM84	Body;TSS200	OpenSea	0.256	0.205	0.051	2.92E-08	3.28E-06
cg23429698	8	98878169			N_Shelf	0.324	0.268	0.056	3.13E-08	3.44E-06
cg23992886	7	116420651	MET;MET	Body;Body	OpenSea	0.635	0.578	0.058	3.43E-08	3.68E-06
cg00399027	16	85676861	KIAA0182	5′UTR;Body	S_Shore	0.607	0.547	0.060	3.72E-08	3.89E-06
cg04042861	2	231989824	PSMD1;HTR2B	TSS200;Body	OpenSea	0.406	0.346	0.060	4.25E-08	4.29E-06
cg18835596	6	135540107	MYB	3′UTR	OpenSea	0.725	0.659	0.066	4.46E-08	4.45E-06
cg03182584	7	36364854	KIAA0895	3′UTR	OpenSea	0.689	0.631	0.058	4.53E-08	4.49E-06
cg00105306	15	85194978	WDR73	Body	N_Shelf	0.626	0.569	0.057	4.88E-08	4.75E-06
cg24499605	1	3142925	PRDM16	Body;Body	OpenSea	0.550	0.489	0.061	5.85E-08	5.41E-06
cg07589393	7	26984305			OpenSea	0.724	0.674	0.050	6.01E-08	5.52E-06
cg09722609	5	149887422	NDST1	TSS1500	OpenSea	0.607	0.551	0.055	6.05E-08	5.54E-06
cg04339360	13	73635568	KLF5	Body	S_Shore	0.710	0.644	0.066	6.23E-08	5.67E-06
cg20426710	22	50248907	ZBED4	5′UTR	N_Shore	0.519	0.461	0.057	6.33E-08	5.74E-06
cg07264238	17	33474376	UNC45B	TSS1500	OpenSea	0.778	0.707	0.071	7.23E-08	6.32E-06
cg25718604	20	57601474	TUBB1	3′UTR	S_Shelf	0.831	0.774	0.057	7.34E-08	6.40E-06
cg19683780	8	42907576			N_Shelf	0.784	0.733	0.051	7.67E-08	6.60E-06
cg12449049	6	25088999	CMAH	Body	OpenSea	0.686	0.636	0.050	8.32E-08	6.97E-06
cg00864012	17	62294665	TEX2	5′UTR	OpenSea	0.724	0.670	0.053	8.38E-08	7.01E-06
cg00991744	12	41581484	PDZRN4	TSS1500	N_Shore	0.852	0.791	0.061	8.42E-08	7.04E-06
cg09620718	16	20702872	ACSM1	TSS1500	OpenSea	0.784	0.733	0.051	8.51E-08	7.08E-06
cg08066875	2	163197084			N_Shelf	0.637	0.581	0.056	8.63E-08	7.15E-06
cg08324152	19	57172404			N_Shelf	0.283	0.216	0.067	9.18E-08	7.49E-06
cg05825244	20	2730488	EBF4	Body	Island	0.428	0.271	0.157	9.32E-08	7.58E-06
cg00876141	4	47837775	CORIN	Body	N_Shore	0.451	0.396	0.055	9.35E-08	7.59E-06
cg06096336	2	231989800	PSMD1;HTR2B	Body;1stExon;5′UTR	OpenSea	0.571	0.519	0.052	9.83E-08	7.88E-06
cg11329129	11	705692	EPS8L2	TSS1500	N_Shore	0.417	0.367	0.051	9.86E-08	7.89E-06
cg12876838	4	5897223			S_Shelf	0.754	0.702	0.052	1.10E-07	8.58E-06
cg26475911	17	73056187	KCTD2	Body	OpenSea	0.458	0.390	0.067	1.14E-07	8.78E-06
cg13561554	15	78795944			N_Shelf	0.620	0.567	0.053	1.19E-07	9.04E-06
cg26413942	5	124081751	ZNF608	TSS1500	OpenSea	0.421	0.361	0.060	1.24E-07	9.27E-06
cg24263062	20	2730191	EBF4	Body	Island	0.636	0.550	0.086	1.30E-07	9.59E-06
cg13958199	9	127018815	NEK6	TSS1500	N_Shore	0.682	0.631	0.051	1.33E-07	9.77E-06

Next, the 1,309 selected CpG sites were ordered by chromosome and position to identify 3 or more adjacent sites in the same gene. We identified 18 genes consisting of 60 individual CpG sites (Table 3). The majority of the genes (N = 14) had 3 adjacent significant CpG sites; 3 genes (*FOXP1*, *FNDC3B*, *RPS6KA2*) had 4 adjacent sites, and 1 gene (*EBF4*) had 6 adjacent sites.

**Table 3:** Adjacent sites (3 or more) on the same gene among the selected CpG sites (N = 1,309) with methylation levels associated with HIV after adjustment for age, sex, and cell type proportion, sorted by chromosome and position.

CpG	CHR	Position	Gene	Relation to Gene	Relation to CpG Island	β (HIV+)	β (HIV−)	Δβ	P	Adj P
cg24499605	1	3142925	PRDM16	Body	OpenSea	0.55	0.489	0.061	5.85E-08	5.41E-06
cg22497969	1	3143018	PRDM16	Body	OpenSea	0.784	0.728	0.057	7.34E-07	3.35E-05
cg26300135	1	3324243	PRDM16	Body	S_Shore	0.904	0.835	0.069	1.23E-02	3.34E-02
cg07330114	3	11624023	VGLL4	Body;TSS200	OpenSea	0.238	0.177	0.06	7.50E-05	9.33E-04
cg00691123	3	11632974	VGLL4	Body	OpenSea	0.573	0.521	0.052	1.93E-02	4.66E-02
cg25357825	3	11697138	VGLL4	Body	OpenSea	0.344	0.294	0.051	1.20E-04	1.30E-03
cg18067134	3	71084634	FOXP1	Body	OpenSea	0.728	0.667	0.061	3.56E-05	5.50E-04
cg08173709	3	71103443	FOXP1	Body	OpenSea	0.726	0.669	0.057	5.42E-07	2.71E-05
cg20382675	3	71591429	FOXP1;MIR1284	5′UTR;TSS200;5′UTR	OpenSea	0.542	0.487	0.055	6.38E-03	2.06E-02
cg18558767	3	71592056	FOXP1;MIR1284	5′UTR;TSS1500;5′UTR	OpenSea	0.442	0.391	0.051	1.19E-02	3.25E-02
cg06508738	3	1.39E + 08	PRR23C	TSS200	Island	0.623	0.567	0.057	3.98E-05	5.95E-04
cg24530147	3	1.39E + 08	PRR23C	TSS200	Island	0.773	0.707	0.066	5.35E-05	7.34E-04
cg14325153	3	1.39E + 08	PRR23C	TSS200	Island	0.537	0.47	0.067	2.01E-04	1.86E-03
cg17797940	3	1.72E + 08	FNDC3B	5′UTR	S_Shore	0.539	0.479	0.061	9.52E-04	5.46E-03
cg14016236	3	1.72E + 08	FNDC3B	5′UTR	OpenSea	0.494	0.424	0.07	2.50E-03	1.07E-02
cg06740950	3	1.72E + 08	FNDC3B	Body;Body	OpenSea	0.473	0.416	0.057	1.19E-04	1.29E-03
cg09357350	3	1.72E + 08	FNDC3B	Body;Body	OpenSea	0.641	0.583	0.058	9.07E-04	5.27E-03
cg17009978	3	1.84E + 08	VWA5B2;MIR1224	Body;TSS200	N_Shore	0.719	0.667	0.052	2.84E-08	3.21E-06
cg12821315	3	1.84E + 08	VWA5B2;MIR1224	Body;TSS200	N_Shore	0.822	0.772	0.05	5.97E-07	2.90E-05
cg21545762	3	1.84E + 08	VWA5B2;MIR1224	Body;TSS200	Island	0.562	0.51	0.052	1.79E-06	6.39E-05
cg01734045	4	1.1E + 08	COL25A1	Body	OpenSea	0.292	0.24	0.052	9.82E-07	4.14E-05
cg08760493	4	1.1E + 08	COL25A1	Body	OpenSea	0.533	0.48	0.053	5.18E-04	3.59E-03
cg11165912	4	1.1E + 08	COL25A1	Body	N_Shelf	0.258	0.207	0.051	1.25E-05	2.61E-04
cg00421164	6	7111068	RREB1	5′UTR	S_Shore	0.827	0.774	0.053	1.90E-04	1.79E-03
cg09754197	6	7113738	RREB1	5′UTR	S_Shelf	0.741	0.69	0.051	2.60E-05	4.40E-04
cg09326832	6	7243643	RREB1	Body	N_Shelf	0.25	0.198	0.051	3.04E-03	1.22E-02
cg10718056	6	28884599	TRIM27	Body	OpenSea	0.515	0.462	0.053	1.38E-03	7.05E-03
cg10401017	6	28887586	TRIM27	Body	N_Shelf	0.83	0.767	0.063	3.89E-04	2.94E-03
cg05216056	6	28887836	TRIM27	Body	N_Shelf	0.646	0.595	0.051	7.20E-03	2.25E-02
cg11387340	6	1.67E + 08	RPS6KA2	Body	OpenSea	0.366	0.314	0.052	3.52E-03	1.35E-02
cg23464284	6	1.67E + 08	RPS6KA2	Body	OpenSea	0.532	0.457	0.076	1.67E-02	4.18E-02
cg13072943	6	1.67E + 08	RPS6KA2	Body	OpenSea	0.619	0.568	0.051	7.96E-03	2.42E-02
cg05791862	6	1.67E + 08	RPS6KA2	Body	OpenSea	0.734	0.683	0.051	4.65E-05	6.64E-04
cg00084338	6	1.71E + 08	DLL1	Body	N_Shore	0.598	0.495	0.103	2.67E-05	4.49E-04
cg27246129	6	1.71E + 08	DLL1	Body	N_Shore	0.55	0.48	0.07	2.71E-05	4.52E-04
cg15931921	6	1.71E + 08	DLL1	Body	N_Shore	0.748	0.669	0.08	1.95E-05	3.59E-04
cg19161559	7	1.01E + 08	CUX1	Body	OpenSea	0.72	0.669	0.052	5.42E-05	7.40E-04
cg23445003	7	1.02E + 08	CUX1	Body	OpenSea	0.575	0.515	0.061	1.80E-02	4.43E-02
cg17974145	7	1.02E + 08	CUX1	Body	OpenSea	0.617	0.561	0.055	2.14E-03	9.56E-03
cg24786658	7	1.13E + 08	GPR85	TSS1500;5′UTR	OpenSea	0.705	0.647	0.058	1.90E-05	3.52E-04
cg25263801	7	1.13E + 08	GPR85	TSS1500;1stExon;5′UTR	OpenSea	0.719	0.664	0.055	8.11E-05	9.85E-04
cg09506675	7	1.13E + 08	GPR85	TSS1500;TSS200	OpenSea	0.546	0.491	0.055	8.81E-04	5.17E-03
cg10071929	9	1.31E + 08	CIZ1	TSS1500;5′UTR	N_Shore	0.189	0.138	0.051	6.18E-03	2.02E-02
cg13642260	9	1.31E + 08	CIZ1	5′UTR	Island	0.215	0.164	0.05	1.04E-02	2.94E-02
cg09976142	9	1.31E + 08	CIZ1	5′UTR	Island	0.296	0.236	0.06	8.49E-03	2.54E-02
cg01832662	14	86086389	FLRT2	5′UTR	OpenSea	0.231	0.173	0.058	1.24E-02	3.34E-02
cg26235748	14	86087511	FLRT2	5′UTR	OpenSea	0.531	0.454	0.077	1.01E-05	2.23E-04
cg22859727	14	86090320	FLRT2	3′UTR	OpenSea	0.432	0.38	0.052	1.75E-06	6.28E-05
cg23047825	15	99317340	IGF1R	Body	OpenSea	0.791	0.728	0.063	4.84E-03	1.70E-02
cg08920032	15	99332004	IGF1R	Body	OpenSea	0.623	0.568	0.055	2.51E-03	1.07E-02
cg10342963	15	99443213	IGF1R	Body	OpenSea	0.759	0.709	0.05	6.58E-03	2.11E-02
cg16616918	17	1686627	SMYD4	Body	OpenSea	0.692	0.635	0.056	8.23E-05	9.95E-04
cg09940188	17	1686671	SMYD4	Body	OpenSea	0.599	0.538	0.061	4.37E-04	3.19E-03
cg23679982	17	1686737	SMYD4	Body	OpenSea	0.636	0.57	0.066	9.76E-05	1.12E-03
cg13518079	20	2675072	EBF4	Body	S_Shore	0.35	0.262	0.088	3.33E-07	1.90E-05
cg05857996	20	2675418	EBF4	Body	S_Shore	0.786	0.686	0.101	6.88E-06	1.69E-04
cg16426670	20	2675996	EBF4	Body	S_Shore	0.812	0.75	0.062	2.29E-08	2.74E-06
cg24263062	20	2730191	EBF4	Body	Island	0.636	0.55	0.086	1.30E-07	9.59E-06
cg05825244	20	2730488	EBF4	Body	Island	0.428	0.271	0.157	9.32E-08	7.58E-06

### Analysis of differentially-methylated regions (DMRs) associated with HIV

Using a separate procedure, we searched for genomic regions (in a window of 1000 bases) where DNAm was associated with HIV in a coordinated manner. We found 315 DMRs associated with HIV after adjustment for age, sex, and cell type proportion with Stouffer p-value < 0.05, maximum |Δβ| change in the region >0.05, and containing at least 2 or more CpG sites (Table S2). The top 10 regions were associated with the following genes, with 1 region not mapping to any gene and 4 regions mapping to 2 or more genes: *COL11A2*, *NOTCH4*, *DLL1*, *FAM120B*, *SLC25A2*, *GTF2H4*, *VARS2*, *RN7SL175P*, *HCG22*, *SPATA8-AS1*, *TAP*1, *PSMB9*, *HOXB3*, *HOXB4*, *MIR10A*. Four of these genes (*DLL1*, SLC25A2, HCG22, and PSMB9) were also identified by the 1,309 selected CpG sites in the previous section (Table S1). Overall, 81 of the 315 regions were associated with genes that were previously identified by the 1,309 selected CpG sites (Tables S1 and S2). 10 regions were associated with genes previously identified by the 69 CpG sites meeting the Bonferroni threshold, including *HTR2B*, *XDH*, *PDZRN4* (2 regions), *SPERT*, NLRC5, *UNC45B*, *EBF4* (2 regions), and *ZBED4*.

**Figure 2:**
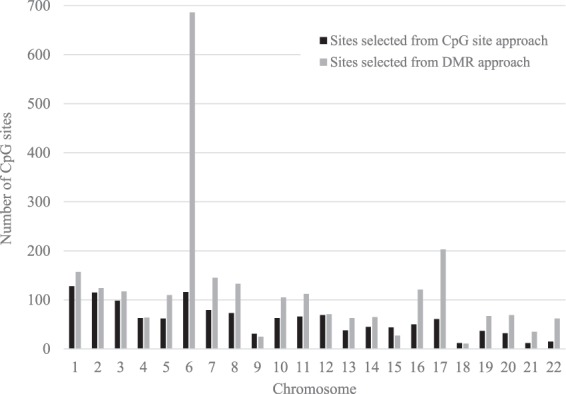
Number of individual CpG sites selected by the CpG approach (N = 1,309) and by the differentially methylated region (DMR) approach (N = 2,572) by location on the chromosome.

Figure 2 shows the total number of individual CpG sites selected by the CpG site approach (N = 1,309) and by the DMR approach (N = 2,572) by location on the chromosome. In the CpG site approach, a large number of significant sites were observed on chromosomes 1, 2, 3, and 6. In the DMR approach, the largest number of significant sites were observed on chromosome 6. In addition, 6 of the top 10 regions were located on chromosome 6.

**Figure 3:**
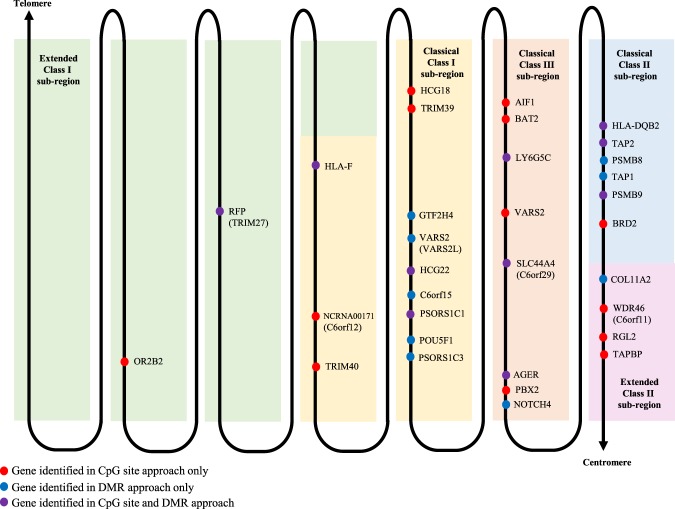
Gene map of the extended major histocompatibility complex (xMHC) on chromosome 6 highlighted with genes identified in the genome-wide site and region approach. Figure adapted from Horton *et al*.^58^.

On chromosome 6, a large number of the genes identified in the CpG site approach as well as the DMR approach were located in the MHC region. Gene locations on the MHC region were mapped in a figure adapted from Horton *et al*.^18^ (Fig. 3), with red indicating genes identified in the CpG site approach only, blue indicating genes identified in the DMR approach only, and purple indicating genes identified in both. Overall, 13 genes in the MHC classical class I sub-region, 6 genes in the class II sub-region, 8 genes in the class III sub-region, 2 genes in the extended class I sub-region, and 4 genes in the extended class II sub-region were found to be associated with differential DNAm in HIV.

### Gene-set enrichment analyses

Among the 1,309 DM HIV-associated CpG sites, 973 mapped to genes, including 862 unique genes. Gene-set enrichment analysis of these genes identified 95 significant biological processes (Table S3). Top processes included regulation of multicellular organismal process, negative regulation of biological process, negative regulation of cellular process, and cell adhesion. 203 of the 315 selected DMRs mapped to at least 1 known gene. However, gene-set enrichment analysis of this latter set of genes did not find any significant results.

### Genome-wide analysis of CpG sites with differential DNAm associated with HIV, stratified by sex

In a repeat analysis stratified by sex, 1,078 CpG sites were selected by the same criteria (FDR q-value < 0.05 and |β| > 0.05) for boys and 555 CpG sites were selected for girls. A total of 60 CpG sites overlapped between boys and girls, and all 60 were among the 1,309 CpGs selected in the overall analysis (Table S4), including CpG sites in *EBF4*, *NLRC5*, *DLL1*, and *PRDM16*.

The relationship between the selected CpG sites identified in the analysis of all children compared to the selected CpG sites in the analyses in boys and analyses in girls is shown in Fig. S2. 750 CpG sites were unique to boys only and 272 CpG sites were unique to girls only. For boys the top CpG site (smallest P-value) was located in the gene body of *CLNK*. For girls the top CpG site was located in the gene body of *GMDS*. We also ordered CpG sites by chromosome and position to identify 3 or more adjacent sites in the same gene. 9 genes met this criteria for boys and 6 genes met this criteria for girls (Table S5). 2 CpG sites for boys (*MAN1B1*, 20.5%; *ADAMTS17*, 23.3%) and 1 CpG site for girls (*TRIM2*, 23.7%) had more than a 20% DNAm difference between HIV-infected and uninfected children (all were hyper-methylated for HIV-infected children).

### Genome-wide CpG sites with differential DNAm, stratified by age at starting ART

Next, we repeated the analysis of CpG sites associated with HIV status, stratified by age at ART start. The mean (SD) age at ART initiation was 3.2 (1.3) for those starting ART <6 months and 12.3 (5.5) for those starting ART 6–24 months of age. After adjustment for sex, age, and cell type proportion, 1,692 CpG sites were DM between HIV-infected children starting ART <6 months of age and HIV-uninfected children, and 738 CpG sites were DM between HIV-infected children starting ART 6–24 months of age and HIV-uninfected children. A total of 390 CpG sites overlapped between children starting ART <6 months and 6–24 months, and all 390 were among the 1,309 CpGs selected in the overall analysis (Table S6), including CpG sites in *EBF4*, *NLRC5*, *DLL1*, *FOXP1*, and *PRDM16*.

The concordance between the selected CpG sites identified in the analysis of all children compared to the selected CpG sites in the analysis of HIV-infected children starting ART <6 months and the analysis of HIV-infected children starting ART 6–24 months is shown in Fig. S3. 800 sites were unique to children starting <6 months and 155 CpG sites were unique to children starting 6–24 months. Among the 800 unique sites to the analysis of children who started ART <6 months, the top 3 CpG sites were in *B3GNT7*, *MYPN*, and *SUV420H1*. We also ordered the 800 CpG sites by chromosome and position to identify 3 or more adjacent sites in the same gene. Identified genes included *JARID2*, *LMTK3*, *DKFZp761E198*, *SHANK2*, *SLITRK5*, *SLC25A2*, *C1orf35*, as well as genes part of the protocadherin gamma gene cluster (PCDHG). The top CpG site among the 155 CpG sites unique to the analysis of children who started ART 6–24 months was in *LARGE*. Ordering the 155 CpG sites by chromosome and position identified *COLEC11* as having 3 or more adjacent sites in the same gene.

### Validation of selected genes by targeted bisulfite sequencing

**Figure 4:**
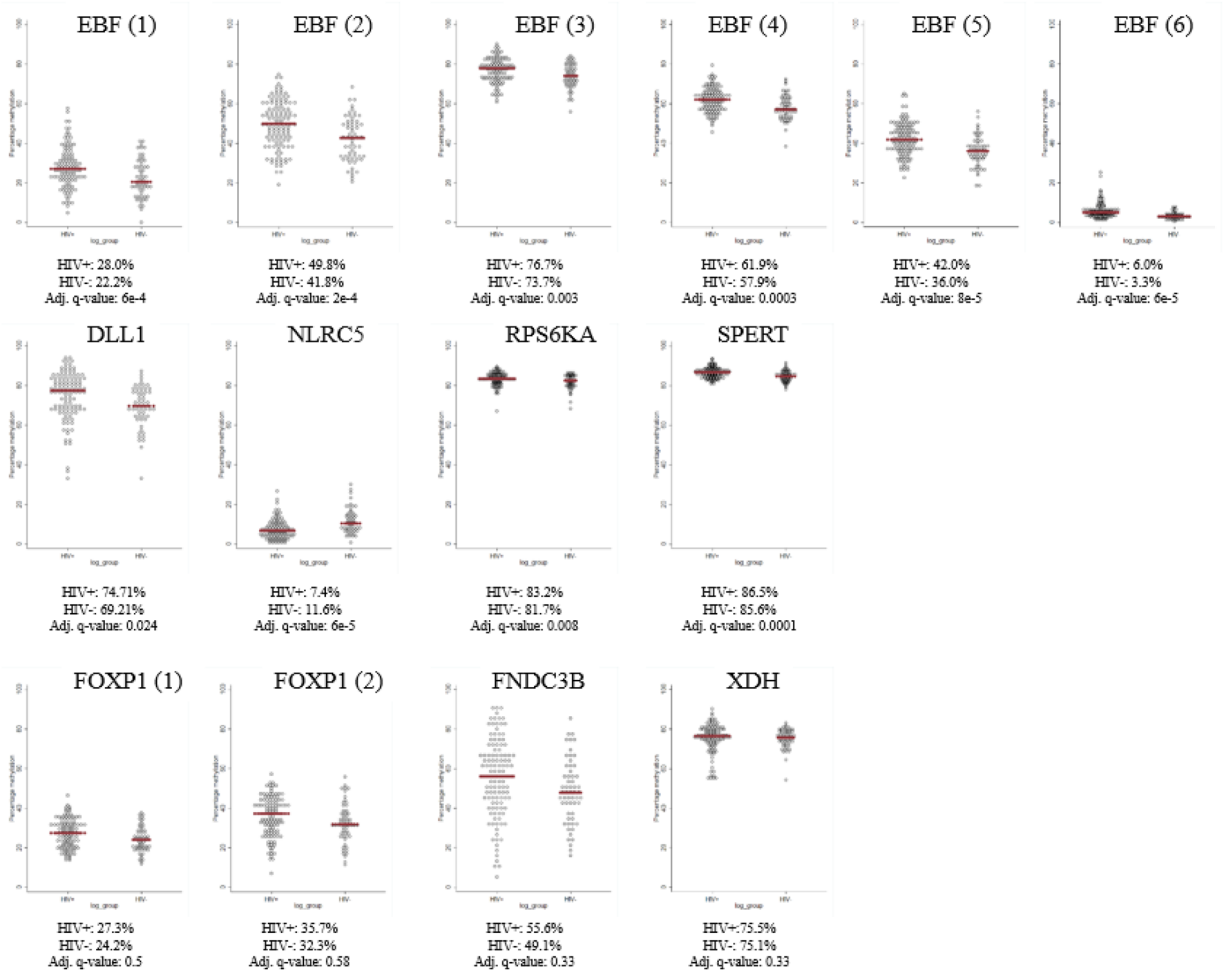
CpG methylation values (percentage) in HIV-infected and HIV-uninfected groups in 14 gene regions from targeted bisulfite sequencing. The red line represents the mean methylation. Adjusted q-values are from multivariable linear regression with methylation percentage as the dependent variable and HIV status as the primary independent variable, along with sex, cell composition and age as covariates.

We selected 16 amplicons covering 9 genes of interest (*EBF4*, *XDH*, *SPERT*, *NLRC5*, *FOXP1*, *FNDC3B*, *RPS6KA2*, *DLL1*, *HCG22*) for validation by targeted bisulfite sequencing (Table S7). 1 amplicon (*HCG22*) did not meet quality control standards. The distributions of methylation values by HIV group in the amplicons that passed quality control are summarized in Fig. 4, with the 2 amplicons for *NLRC5* combined. Mean methylation in all 6 amplicons in *EBF4* was greater for the HIV-infected group after adjustment for sex, blood cell composition, and age. There was also significantly higher methylation in the HIV-infected group at the amplicons queried in *DLL1*, *RPS6KA*, and *SPERT*, and less methylation at the *NLRC5* amplicon. The directions of these associations were consistent with the differences at the CpG sites identified in the 450 K epigenome-wide association study (EWAS) approach, confirming the findings. No significant differences were detected at *FOXP1*, *FNDC3B*, or *XDH*, after adjustment for sex, blood cell composition, and age. Annotation maps for *EBF4*, *DLL1*, *RPS6KA*, *SPERT*, and *NLRC5* are presented in Fig. S4, showing the amplicon location as well as the DM CpG site from the 450K EWAS. Differential methylation for *SPERT* was likely only at the index CpG site from the 450K EWAS.

## Discussion

Our study provides the first evidence that changes in the epigenome are detectable in children with perinatally-acquired HIV infection on suppressive ART. Using a genome-wide approach, we found 1,309 DM CpG sites between treated HIV-infected children and age-matched uninfected children from the same community. Our finding of widespread methylation differences in the MHC region in children with HIV, combined with findings of methylation remodeling in the HLA cluster in adults with HIV^15^, provides strong support of epigenetic modifications by HIV in this region. We found strong evidence for differential methylation in *NLRC5*, which encodes for a transcription factor that regulates MHC class I molecule expression; this gene was also implicated in a study of DNAm in adults with HIV^16^. In addition, we identified differential methylation in other novel host genes that suggest relevant pathways affected by HIV disease and/or its treatments.

In contrast to the mainly adult male studies that found more hypo-methylated sites in HIV-infected individuals compared to HIV-uninfected individuals^15,16^, the substantial majority of the DM CpG sites in our sample of children were hyper-methylated (97%) in the HIV-infected group, even just among boys (99%). These differences, if replicated, suggest that early life exposure to HIV may shape the epigenome differently than later life exposure^19^. In addition, the relation between hyper- or hypo-methylation and gene expression of HLA genes will require further study, as HIV infection has been shown to be associated with both increased and decreased expression of HLA genes^20,21^.

We found significant methylation differences associated with HIV on chromosome 6 in the MHC region which plays an important role in the adaptive immune response^18^. Class I MHC molecules present peptides from inside the cell and are recognized by CD8 T-cells. Although no significant methylation differences were observed in the classical HLA class I genes (e.g. *HLA-A*, *HLA-B*, *HLA-C*), we did detect differences in a non-classical HLA class I gene (*HLA-F*). A laboratory study found *in vitro* that the activating natural killer cell receptor *KIR3DS1* bound to the ligand *HLA-F*, leading to production of antiviral cytokines and inhibition of HIV replication^22^. This study also found that infection of CD4+ T cells with HIV increased transcription of the gene encoding *HLA-F*; our finding suggests this may be due to an epigenetic mechanism. Other genes with differential methylation in this region included *PSORS1C1* and *PSORS1C3*, previously implicated as genes associated with control of HIV in non-progressors in genome-wide association studies, and *POU5F1* which was previously found to have gene expression modulated by treatment with nucleoside reverse transcriptase inhibitors^23–25^.

Class II MHC molecules present peptides from outside the cell and are recognized by CD4 T-cells. We detected differential methylation in *HLA-DQB2*, one of the classical HLA Class II genes. In addition, other genes in the class II region encoding for immune function were DM. *TAP1* and *TAP2* encode for the transporters associated with antigen processing and the movement of HLA molecules. *PSMB8* and *PSMB9* code for components of the immune-proteasome that degrade proteins into peptides for association with MHC class I molecules. There was also differential methylation of class III MHC molecules, including *NOTCH4*, which codes for a receptor in the notch signaling pathway which has been extensively studied in T-cell development^26^. It is possible *NOTCH4* may be affected by exposure to ritonavir-boosted lopinavir, as alterations in this pathway have previously been reported among adults with exposure to protease inhibitors^27^.

Outside of the MHC region, HIV was associated with differential DNAm in other genes related to the adaptive immune system including *NLRC5* and *EBF4*. Both findings were validated in targeted bisulfite sequencing analyses. *NLRC5*, a NOD-like receptor (NLR) family member, encodes for a transcription factor that regulates transcription of MHC class I genes^28–31^. A top CpG site identified to be associated with HIV was in *NLRC5* in a recent adult EWAS by Zhang *et al*.^16^. Although we only detected DM in 1 of 2 significant CpG sites reported in Zhang *et al*., we also found decreased methylation in the group with HIV at cg07839457. *NLRC5* can associate with and activate promoters of MHC class I genes, including *HLA-F* and induce the expression of related genes in the MHC class I pathway, including *TAP1*. *NLRC5* also regulates NF-kB and type I interferon signaling, and can be a potential target for manipulation of immune responses against infectious diseases^32^.

4 out of the 6 DM CpG sites located in *EBF4* met the Bonferroni threshold (cg17069396, cg16426670, cg05825244, and cg24263062) and were hyper-methylated in HIV-infected children. *EBF4* encodes for a transcription factor in the Olf-1/EBF family that is involved in neural development and B-cell maturation; it has been identified as an affected gene in an EWAS of individuals with down syndrome and unaffected relatives as controls^33,34^. B-cell deficiencies have been reported early studies of individuals with HIV, and there is evidence that HIV directly interacts with B-cells and can lead to B-cell hyperactivity via cytokines and growth factors^35,36^. Dysregulated methylation in the *EBF4* region suggests an epigenetic mechanism for these deficiencies is also possible. HIV may also affect methylation of other genes related to B-cell development including *DLL1*, which was confirmed by targeted bisulfite sequencing. *DLL1* helps to mediate cell fate decisions during hematopoiesis, blocking the differentiation of progenitor cells into the B-cell lineage while promoting cells with the characteristics of a T-cell/NK-cell precursor^37^. We observed increased methylation associated with HIV at several CpG sites in *FOXP1*, although not confirmed after adjustment in targeted bisulfite sequencing, a gene in the forkhead box transcription factor family that plays an important role in the regulation of gene transcription during development^38,39^ and has been implicated in the biology of B-cell malignancies^40,41^. *FOXP1* is in the same *Foxp* subfamily as *FOXP3*; these genes encode for a family of transcription factors recognized for its involvement in autoimmune disease, speech and language disorders, and lung development^38,39,42^. *In vitro*, HIV infection of human T-cells increases DNAm in *FOXP3* and downregulates *Foxp3* expression^43^.

Of note, among the top sites found to be associated with HIV in the adult study by Zhang *et al*. was cg11429292, located 10 kb upstream of the CD4 gene, which encodes the primary receptor for HIV-1 and regulates T helper cell function. This CpG site met our cut off for epigenome wide significance (FDR q-value < 0.05, |Δβ| > 0.05) and was also hyper-methylated in HIV-infected children, but did not meet the more stringent Bonferroni threshold.

The top two selected CpG sites associated with HIV ranked by significance were located in *XDH* and *SPERT*. *XDH* (xanthine dehydrogenase) encodes for a protein that can be converted into *XO* (xanthine oxidase), an enzyme that generates reactive oxygen species (ROS) that is present in many organs, including the liver, gut, lung, kidney, heart, and brain as well as the plasma^44^. It has been shown that during states of infection and inflammation, activity of *XO* can increase by 10–50%^45,46^. HIV appears to progress faster during oxidative stress, a state in which there is an overabundance of free radicals. In a study of adults, HIV-infected adults were found to have 22% higher *XO* activity^47^. We saw increased average methylation of *XDH* in HIV-infected children suppressed on ART compared to uninfected children in the overall sample as well as in boys and girls, but this did not remain significant in targeted bisulfite sequencing. Further studies are needed to elucidate whether this signal is associated with increased oxidative stress. The role of *SPERT* (spermatid associate) in HIV infection is unknown, but significant methylation differences were found in targeted bisulfite sequencing.

60 of the CpG sites selected in the male-only and female-only analyses were among the CpG sites selected in the sex-combined analysis, including many in implicated genes discussed above (e.g. *EBF4*, *DLL1*, *NLRC5*) and confirming their association with HIV in children of both sexes. Top implicated genes unique to one sex may require special attention and further investigation. For boys, *CLNK* codes for a protein that plays a role in the regulation of immunoreceptor signaling, *MAN1B1* encodes an enzyme belonging to the glycosyl hydrolase 47 family, which can cause autosomal-recessive intellectual disability when mutated, and *ADAMTS17* codes for a member of the *ADAMTS* protein family which can remodel the extracellular matrix. For girls, *GMDS* encodes for a protein that plays a role in biosynthesis of GDP-fucose and *TRIM2* encodes for a protein that is part of the TRIM family and plays a neuroprotective role. Our review of the literature did not identify any studies reporting known differences in these genes between boys and girls.

Although our stratified analysis of age at starting ART (<6 months vs. 6–24 months) confirmed many of the CpG sites in the combined analysis in their overlap, it also identified a large number of non-overlapping DM CpG sites unique to children who start ART <6 months and unique to children who start ART6–24 months. Although the groups in this study are defined by the age at which they started ART, the differences could also be due to a longer duration on ART in the children who started ART at <6 months. Thus, we are unable to separate the potential age-related effects of treatment versus time of treatment. Continued follow-up of our cohort, or similar type of cohorts, where methylation markers are measured after a similar length of treatment can be used to disentangle the age and time of treatment effects. In addition, future focused studies of the unique genes identified in our analysis using targeted bisulfite sequencing are warranted to better understand the role of ART timing and duration.

This study has several limitations. First, we used whole blood to assess DNAm differences, as cell sorting prior to DNAm studies was not possible in this setting. To adjust for methylation signals due to different cell types, we used a method to adjust for cellular heterogeneity that has been widely used in other EWAS^48^. Although it has been noted that this algorithm may not be adequate in studies of young children, other methods have not yet been developed^49^, we felt confident that adjusting for cell types using this method was appropriate in our study given the high correlation between the methylation-estimated and the laboratory-estimated CD4+ T-cell percentage. Second, our study demonstrated changes in the epigenome in children with perinatally-acquired HIV on suppressive ART, but cannot disentangle HIV from ART exposures. The epigenome of these children may have been affected intrauterine by their mother’s HIV status or antiretroviral use as well as their own chronic HIV infection and ART exposures. We specifically selected a homogeneous group of HIV-infected children who were started on treatment early before 2 years of age and all suppressed on ritonavir-boosted lopinavir-based regimens. Therefore, due to lack of variability in the sample, we were unable to assess associations between methylation differences at specific CpG sites and clinical outcomes such as HIV RNA quantity or CD4 percentage. Comparison of HIV-infected and uninfected children at age 4–9 years does not allow for separate consideration of ART- vs. HIV-related effects on DNAm. Due to the recent emphasis on early identification and prompt initiation of treatment in all children under 5 years, inclusion of untreated HIV-infected children of this age is neither feasible nor ethical. *In utero* ART exposure may also have influenced DNAm in the HIV-infected children. Our study was conducted at a single time point, reducing the ability to understand how the relationship between HIV and ART on DNAm may change throughout childhood or to address the potential for reverse causality. Other study designs to address these concerns are warranted, including longitudinal studies of DNAm differences in children before and after starting ART. Finally, we used the Illumina Infinium HumanMethylation450 BeadChip array, which has been the most popular and practical choice for EWAS to date. However, it only covers ~1.5% of overall genomic CpG sites, which are biased towards promoter and protein-coding regions. Future studies should use the newer EPIC array (850 K) which covers an additional 413,745 new CpG sites, many in other genomic regions.

In conclusion, using high-throughput technology and a genome-wide approach, our study provides evidence that children with perinatally-acquired HIV, who started ART under 2 years of age and are well controlled on ART at 4–9 years of age, have widespread changes in DNAm compared to HIV-uninfected children matched by age and with similar demographic characteristics. Using a robust analysis pipeline that considered both individual sites as well as DMRs and used selection criteria that prioritized statistical significance and overall signal (FDR q-value < 0.05 and |Δβ| > 0.05) with cell proportion correction, we confirmed methylation differences in the MHC region and the *NLRC5* gene reported in adults with HIV on ART^15,16^, and identified new candidate CpG sites that may contribute to our understanding of HIV pathogenesis.

## Materials and Methods

### Study population

The study population consisted of HIV-infected and uninfected children aged 4–9 years participating in an observational cohort study being conducted at two sites in Johannesburg, South Africa: Empilweni Services and Research Unit (ESRU) at Rahima Moosa Mother and Child Hospital and the Perinatal HIV Research Unit (PHRU) at Chris Hani Baragwanath Hospital. The HIV-infected children were former participants in clinical trials at these sites^50–54^ and the HIV-uninfected children included those attending the study site for preventive health services.

From 553 HIV-infected children and 300 HIV-uninfected children enrolled in the cohort, 180 were selected for this analysis in 3 groups as follows. After limiting the HIV-infected group to Black African children who were started on ART ≤24 months of age, consistently on treatment, and currently suppressed (HIV RNA <400 copies/mL) on a LPV/r-based regimen at their enrollment visit, we randomly selected 60 children who started ART <6 months of age (Group 1) and 60 children who started ART 6–24 months of age (Group 2). After excluding any siblings of the selected 120 HIV-infected children from the HIV-uninfected children, we selected a random sample of 60 HIV-uninfected children frequency matched by age at enrollment (Group 3).

Children provided assent if they were old enough to do so and displayed adequate mental capacity, and a parent and/or legal guardian provided informed consent. The study was approved by the Institutional Review Boards of Columbia University in New York, USA and the University of the Witwatersrand in Johannesburg, South Africa. All methods were performed in accordance with the relevant guidelines and regulations.

### DNAm quantification

At their enrollment visit in the cohort study, each participant had blood samples obtained under standardized conditions and kept frozen at −80 °C. Samples were shipped on dry ice from Johannesburg, South Africa to Columbia University Medical Center (CUMC) in New York, NY, USA. Genomic DNA (50 ng/µL) was isolated and quantified with PicoGreen (Life Technologies, Carlsbad, CA) by the Biomarkers Shared Resource at CUMC. All but one (N = 179) samples yielded sufficient DNA.

DNAm levels were measured at the Genomics Shared Resource at Roswell Park Cancer Institute (Buffalo, NY). Genomic DNA samples were bisulfite converted using the EZ DNA Methylation kit (Zymo, Inc.), according to the manufacturer’s instructions. The samples were then amplified, enzymatically fragmented, and hybridized to Infinium HumanMethylation450 BeadChips (Illumina, San Diego, CA). Samples were randomly distributed across the BeadChips taking into account group, sex, and age to minimize potential batch effects^55^. Following hybridization, the chips were stained, washed, and scanned using the Illumina iScan System and raw intensity data (IDAT) files were obtained.

### Filtering, normalization, estimation of cell type proportions, and batch effects

Pre-processing pipeline procedures, including filtering and normalization was performed with the R/Bioconductor package *RnBeads*^56,57^. A detailed description of the probes filtered can be found in Table S8. A total of 370,683 CpG sites remained for analysis. In addition, background subtraction was performed using the normal out of band background (*noob*) method provided in the methylumi package^58^. Probe type adjustment to correct for Infinum I/II probe types was done using the Beta Mixture Quantile Method (BMIQ) method^59^.

The DNAm status at each CpG site (β-value) was calculated using the intensities of the methylated (M) and unmethylated (U) probe intensities: β-value = M/(M + U + α), and α is an arbitrary offset (100) to stabilize β-values. As recommended, β-values were logit transformed to the M-value scale for statistical testing^60^.

Since whole blood is a heterogeneous tissue consisting of different cell types, we accounted for potential confounding by cell type. The Houseman algorithm was used to estimate the proportions of cell types in our samples based on the Reinius *et al*. adult reference dataset using the *minfi* package^48,61^. Due to composition differences between cord blood and blood in childhood, an adult reference dataset was used rather than a cord blood reference dataset^62^. To evaluate the plausibility of the estimates, we compared the estimated mean cell percentages of white blood cells to two pediatric references^63,64^ as well as mean differentials from one of our previous studies^50–52^ (Table S9). We then compared estimated mean cell percentages of lymphocytes subsets to a pediatric reference^65^ (Table S10). Next, we compared the methylation-estimated CD4+ T-cell percentage to a measurement by laboratory assay (Fig. S5) and found the two to be highly correlated (r = 0.71, p < 0.001) and acceptable.

Estimated mean blood cell percentages of white blood cells and lymphocytes for HIV-infected and HIV-uninfected children, as well as for HIV-infected children who started ART <6 months and 6–24 months are presented in Table S11. Compared to HIV-uninfected children, HIV-infected children had higher estimated blood cell proportions of CD8 T-lymphocytes and NK lymphocytes, as well as a lower proportion of granulocytes, suggesting it was important to correct for cell type proportion in our analysis.

Although we did not expect there to be batch effects since samples were randomly distributed across the BeadChips by design, we applied a surrogate variable approach to assess for batch effects^66^. We did not detect any uncorrected batch effects and therefore, no surrogate variables were included in the analysis.

### Statistical analysis for epigenome-wide association study

The primary analysis was to identify DM CpG sites associated with HIV status using a genome-wide site approach. To do this, we fit a model using the empirical Bayes moderated linear regression approach implemented by *limma*^67^ with DNAm as the dependent variable and HIV status as the primary independent variable. We conducted an unadjusted analysis as well as an analysis adjusted for age, sex, and cell type proportions. Statistical significance after multiple testing comparison was established using the Benjamini-Hochberg (BH) false discovery rate (FDR) with q-value of 0.05^68^. CpG sites were DM if they had an FDR q-value < 0.05 and |Δβ| > 0.05, where Δβ is the mean difference between the average DNAm of the groups. A more stringent Bonferroni threshold (p < 1.35 × 10^−7^) was also considered. Selected CpG sites were ordered by chromosome and position to identify 3 or more sites on the same gene.

Next, we applied the *DMRcate* region approach described by Peters *et al*. to identify DMRs^69^. This method uses the t-statistic calculated on each CpG site by *limma* and applies a Gaussian kernel smoother with a bandwidth of 1000 base pairs and scaling factor of 2. The model was parameterized identically to the site approach above, with HIV status as the predictor of interest, adjusting for age, sex, and cell type proportions. As part of the package, a Stouffer transformation was applied to combine the *limma* p-values as a metric for ranking. We selected regions with Stouffer p-value < 0.05, maximum |Δβ| change in the region > 0.05, and containing at least two or more CpG sites. We compared the number of CpG sites selected and genes identified by the DMR approach to those identified by the CpG site approach.

CpG sites were annotated using the Illumina 450k annotation file with their relation to CpG islands (Island, Shore, Shelf, Open Sea) as well as to the nearest gene (TSS1500, TSS200, 5′UTR, 1st exon, Body, 3′UTR, Intergenic). DMRs were annotated with their associated gene symbol and genomic areas.

To investigate the potential biological meaning of the genes identified in the loci and region analysis, we conducted gene-set enrichment analysis. To assess significant over-representation of biological processes among the genes associated with these selected sites and regions we used the Database for Annotation, Visualization, and Integrated Discovery (DAVID)^70,71^. Only annotated genes were included in this analysis.

Due to sex differences in response to ART that have been reported in children with HIV^72,73^, we performed secondary sex-stratified analyses, repeating the analysis to identify DM CpG sites associated with HIV in boys and girls; adjustment was made for age and cell type proportion. In addition, given recent interest in the benefits of early ART initiation, we also evaluated if age at ART initiation led to differences in DNAm patterns associated with HIV. We conducted a “stratified” analysis to identify DM CpG sites associated with HIV among those <6 months of age and those 6–24 months of age. Both groups were compared to the HIV-uninfected group; adjustment was made for sex, age, and cell type proportion.

Characteristics of HIV groups were compared using chi-squared or Fisher’s exact tests for categorical variables and t-tests or Wilcoxon tests for continuous variables. All analyses were conducted using the R statistical package version 3.3.2 and SAS software version 9.4 (Cary, North Carolina).

### Validation of analytic pipeline

To validate our analytic pipeline, we performed an EWAS of the 370,683 CpG sites for sex, identifying 5,087 CpG sites associated with sex (FDR q-value < 0.05). We compared our findings to 4 other studies that identified autosomal sex-associated CpG sites^74–77^. Table S12 lists the top 20 significant CpG sites associated with sex in our sample, as well as their ranking among the top sites of the other 4 studies. The top two sites in our sample were cg11643285 located on *RFTN1* (top site in 2 out of 4 other studies) and cg03691818 located on *KRT77* (top site in all 4 other studies). 10/20 of the top sites in Inoshita *et al*., 11/20 of the top sites in Spiers *et al*., 9/20 of the top sites in Xu *et al*., and 9/30 of the top sites in Yousefi *et al*. overlapped with the top 20 sites in our study. Only one CpG site (cg09513416) in our top 20 was not ranked as a top site in the other 4 studies. 46 (78%) of the 60 sites reported by at least one of the other 4 studies were among the top 1000 significant sites in our sample. These results validated our data collection, processing, normalization, and analysis.

### Validation of top hits by targeted bisulfite sequencing

Targeted bisulfite sequencing was utilized for validation and fine-mapping of 16 selected amplicons covering 9 genes of interest (Table S7**)**. In brief, genomic DNA (1 µg) was bisulfite-converted using the EpiTect 96 Bisulfite Kit (Qiagen). Primers were designed in MethPrimer 1, and bisulfite-converted DNA was amplified and multiplexed by PCR on a Fluidigm Access Array high throughput PCR machine^78^. PCRs were performed in triplicates and pooled to ensure sequence complexity. For one locus, NLRC5, since the PCRs were weaker, PCRs from 2 overlapping amplicons were pooled. The library was diluted to a final concentration of 10pM with 35% of PhiX library. Paired-end reads (250 bp) were generated with an Illumina MiSeq sequencer. Fastq files were generated by the MiSeq sequencer. After trimming for low-quality bases (Phred score <30), Illumina and Fluidigm adaptors and reads with a length <40 bp with TrimGalore, the reads were aligned to the human genome (GRC37) using Bismark 2 with the following settings: -D 50 -R 10 -score_min L,0,−0.6^79^. Since the sequences are PCR-based, reads were not de-duplicated. Methylation calling was performed using Bismark extractor. Net methylation was assessed when the coverage was at least 30X and reported by CpG and averaged across amplicon. The median coverage per amplicon ranged from 85x-3440x. In addition, low PCR complexity can reflect less representative estimate of the DNAm level. Therefore, for each amplicon, we identified samples where the complexity (estimated by the number of DNAm patterns represented in at least 1% of the total reads mapping a given amplicon) was less than the mean complexity score of the given amplicon across samples minus 1 standard deviation. The findings were robust after filtering out these samples. For each amplicon, group comparisons were performed using multivariable linear regression with methylation percentage as the dependent variable and HIV status as the primary independent variable, along with sex, cell composition, and age as covariates. FDR q-values were calculated using the BH method.

## Supplementary information

Supplementary Info

## Data Availability

The datasets analyzed during the current study are available from the corresponding author on reasonable request.
