# Supplementary material for: Distinct epigenetic profiles in children with perinatally-acquired HIV on antiretroviral therapy
Source: Sci Rep. 2019 Jul 19;9:10495. doi: 10.1038/s41598-019-46930-1 (PMC6642153; doi:10.1038/s41598-019-46930-1)
Supplement: Supplementary file 1 — Supplementary Info [file 41598_2019_46930_MOESM1_ESM.pdf]

Supplementary Information

Distinct epigenetic profiles in children with perinatally-acquired HIV on antiretroviral therapy

Stephanie Shiau, Renate Strehlau, Shuang Wang, Avy Violari, Catherine Do, Faeezah Patel, Afaaf Liberty, Izabela Krupska, Stephen M. Arpadi, Marc Foca, Ashraf Coovadia, Elaine J. Abrams, Benjamin Tycko, Mary Beth Terry, Louise Kuhn

Fig. S1.

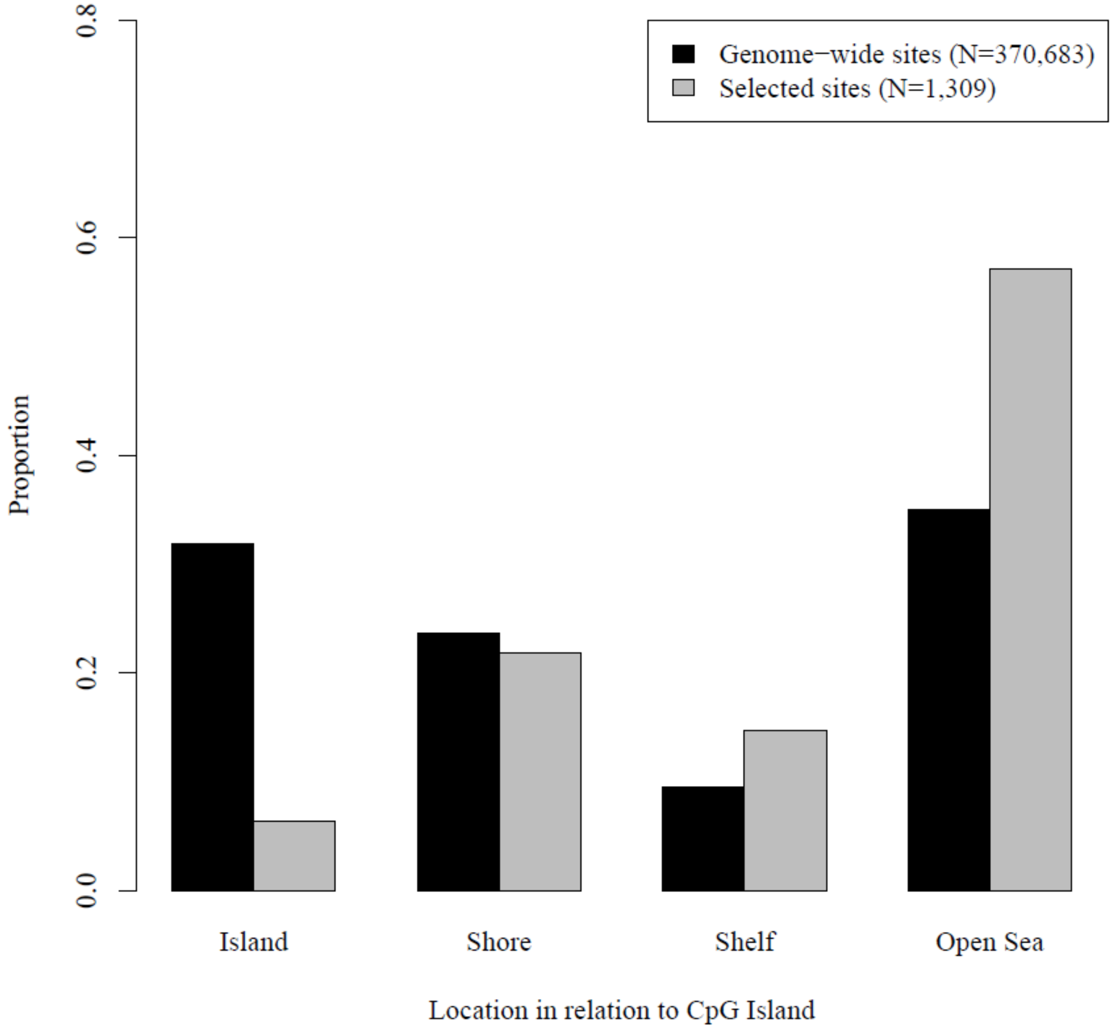

Proportion of CpG site locations in relation to CpG islands for genome-wide CpG sites (N=370,683) and selected CpG sites comparing HIV-infected and uninfected groups (N=1,309)

Fig. S2.

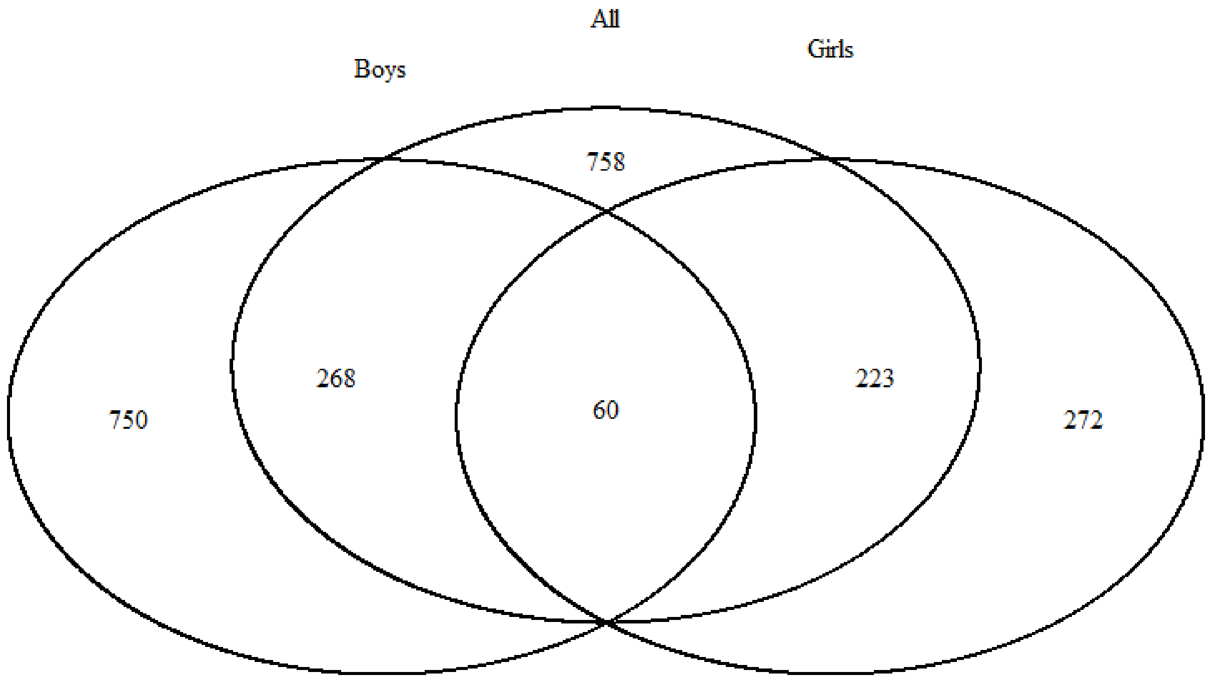

Overlap of selected genome-wide CpG sites with differential methylation associated with HIV in all children (N=1,309), in boys only (N=1,078), and in girls only (N=555)

Fig. S3.

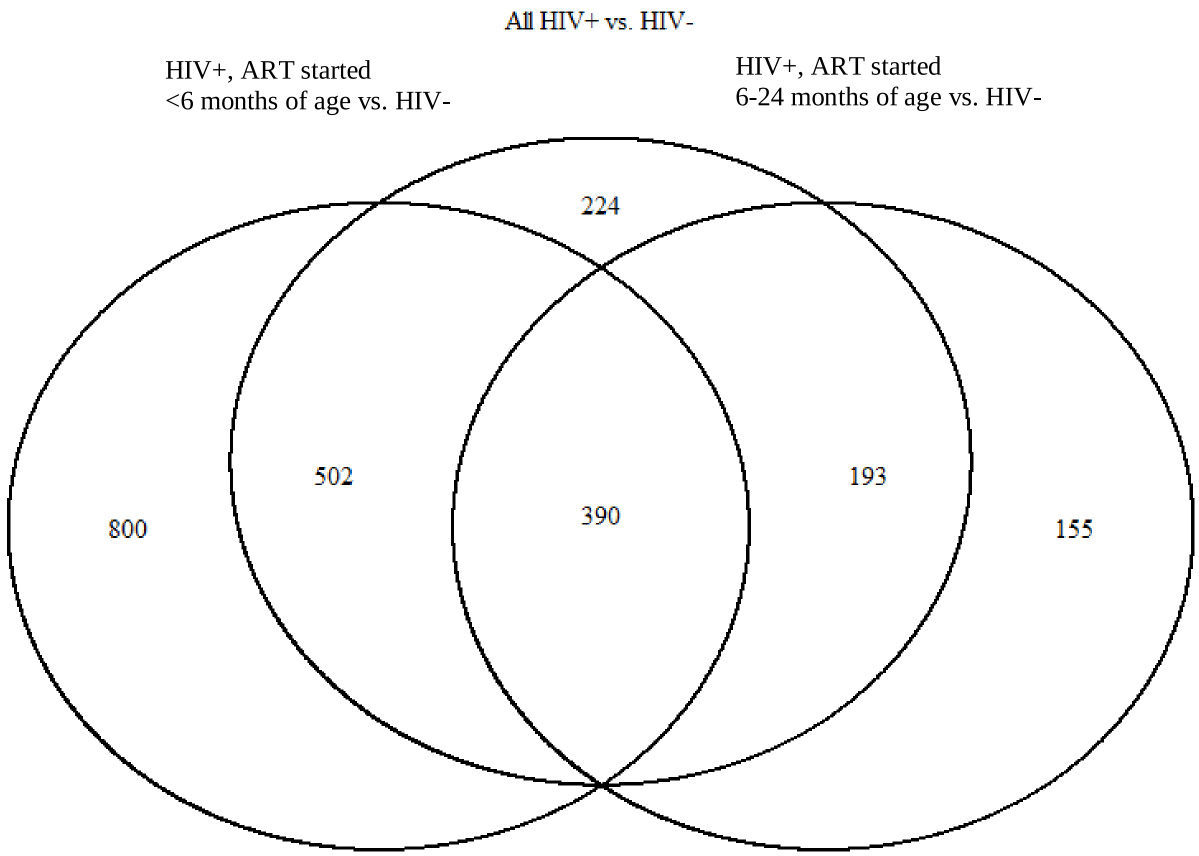

Overlap of selected genome-wide CpG sites with differential methylation associated with HIV in all HIV-infected children compared to all HIV-uninfected children (N=1,309), in HIV-infected children starting antiretroviral therapy (ART) <6 months of age compared to HIV-uninfected children (N=1,692), and in HIV-infected children starting ART 6-24 months of age compared to HIV-uninfected children (N=738)

Fig. S4.

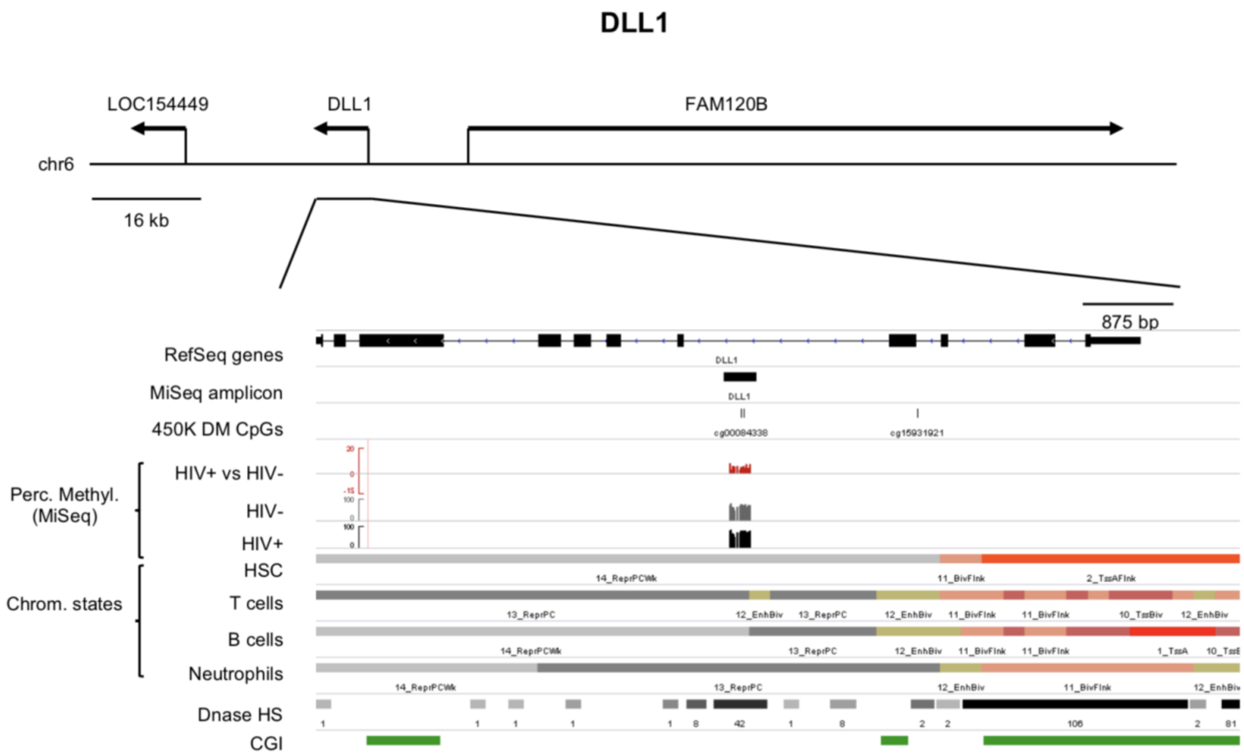
DLL1
LOC154449
DLL1
FAM120B
chr6
16 kb
875 bp
RefSeq genes
MiSeq amplicon
450K DM CpGs
Perc. Methyl. (MiSeq)
HIV+ vs HIV-
HIV-
HIV+
Chrom. states
HSC
T cells
B cells
Neutrophils
Dnase HS
CGI

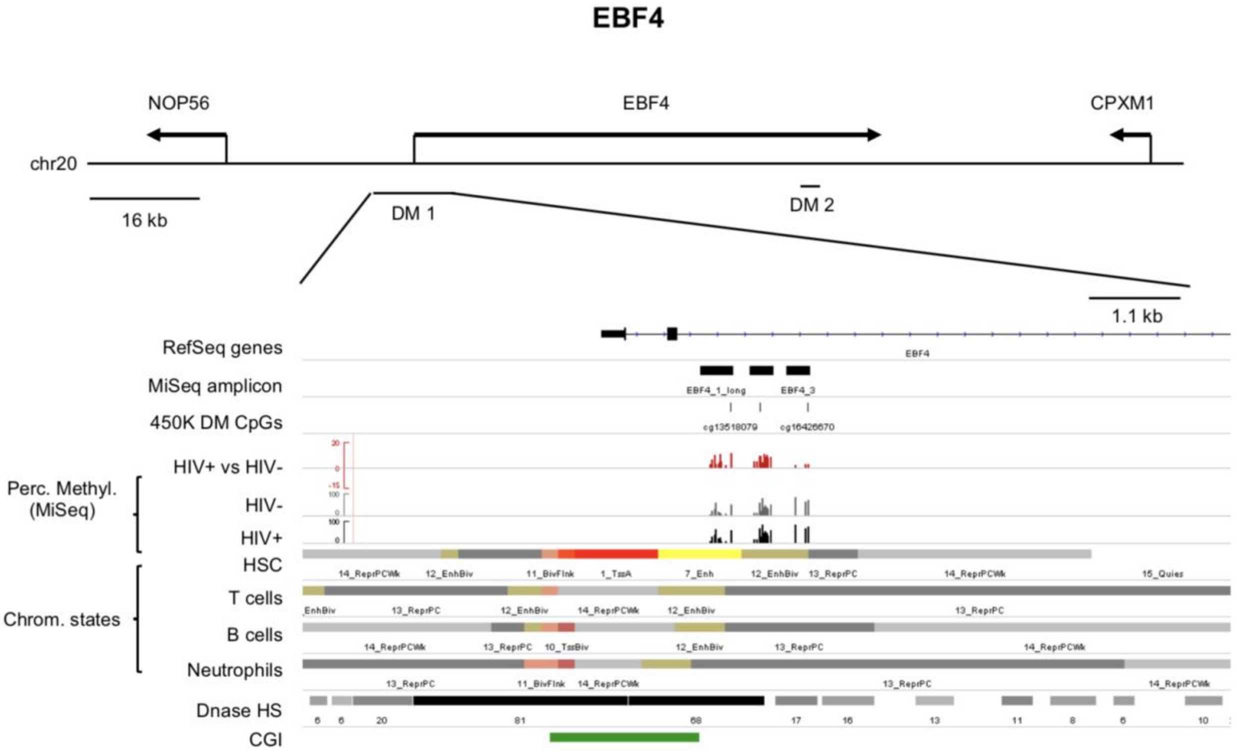
EBF4
NOP56
EBF4
CPXM1
chr20
16 kb
DM 1
DM 2
1.1 kb
RefSeq genes
MiSeq amplicon
450K DM CpGs
Perc. Methyl. (MiSeq)
HIV+ vs HIV-
HIV-
HIV+
Chrom. states
HSC
T cells
B cells
Neutrophils
Dnase HS
CGI

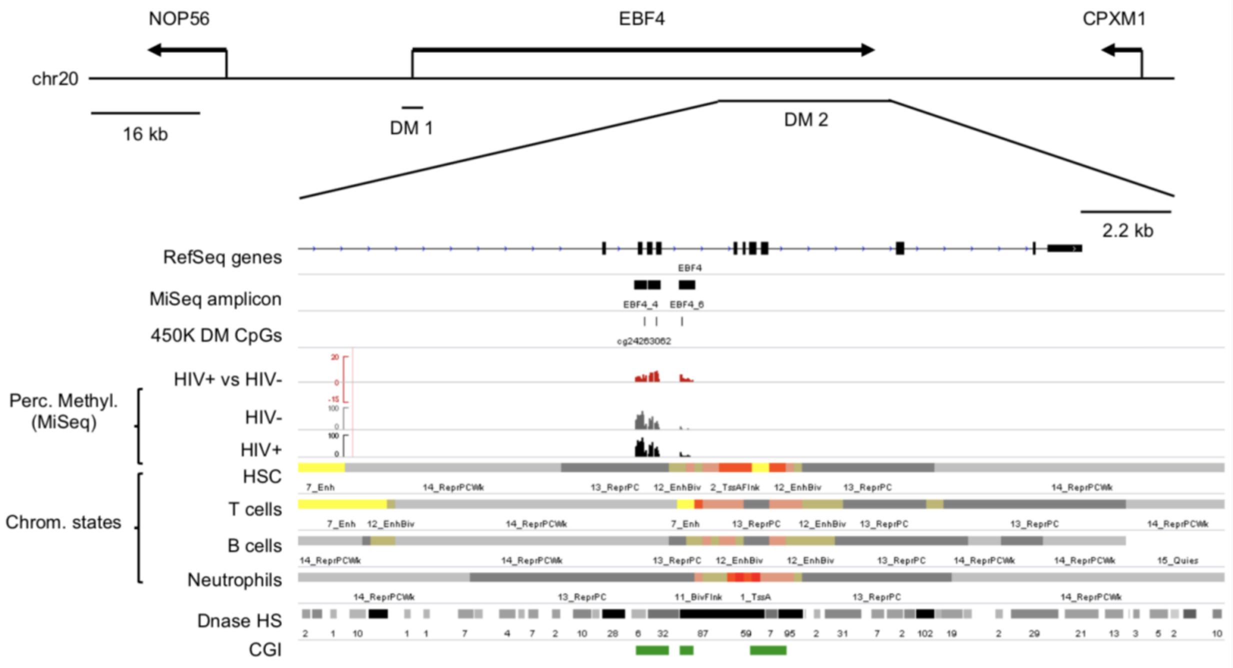
NOP56
EBF4
CPXM1
chr20
16 kb
DM 1
DM 2
2.2 kb
RefSeq genes
MiSeq amplicon
EBF4_4
EBF4_6
450K DM CpGs
cg24263062
Perc. Methyl. (MiSeq)
HIV+ vs HIV-
HIV-
HIV+
Chrom. states
HSC
T cells
B cells
Neutrophils
Dnase HS
CGI

NLRC5

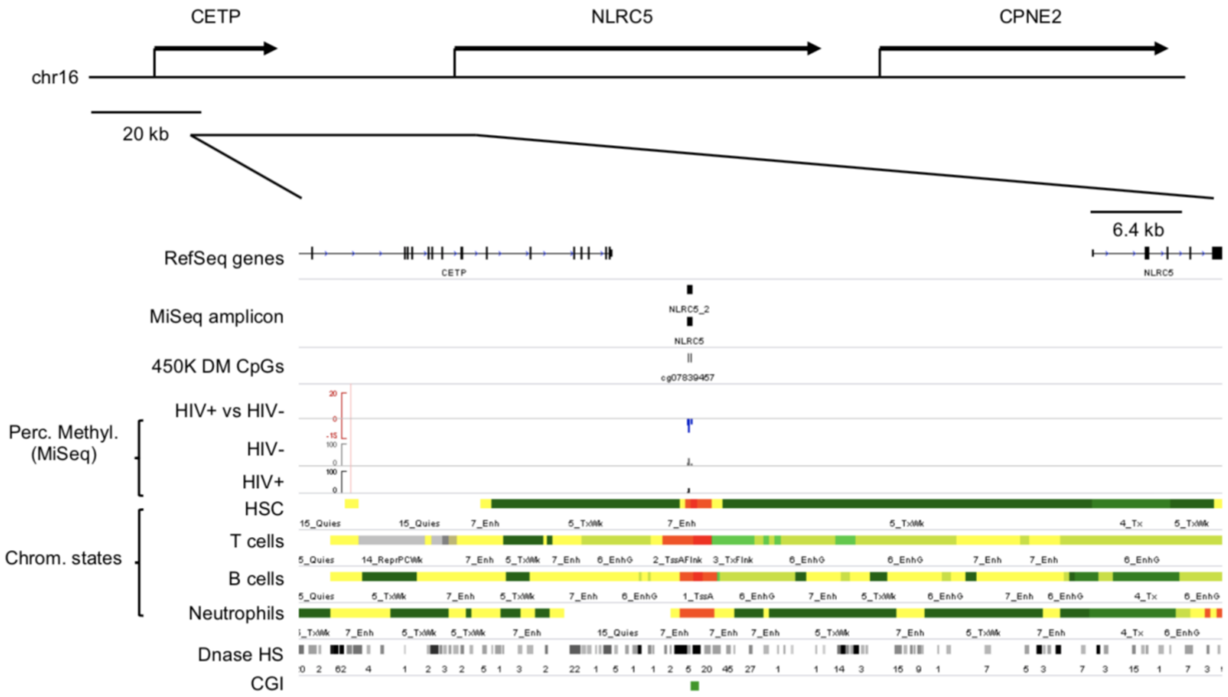
CETP
NLRC5
CPNE2
chr16
20 kb
6.4 kb
RefSeq genes
MiSeq amplicon
NLRC5_2
450K DM CpGs
cg07839457
Perc. Methyl. (MiSeq)
HIV+ vs HIV-
HIV-
HIV+
Chrom. states
HSC
T cells
B cells
Neutrophils
Dnase HS
CGI

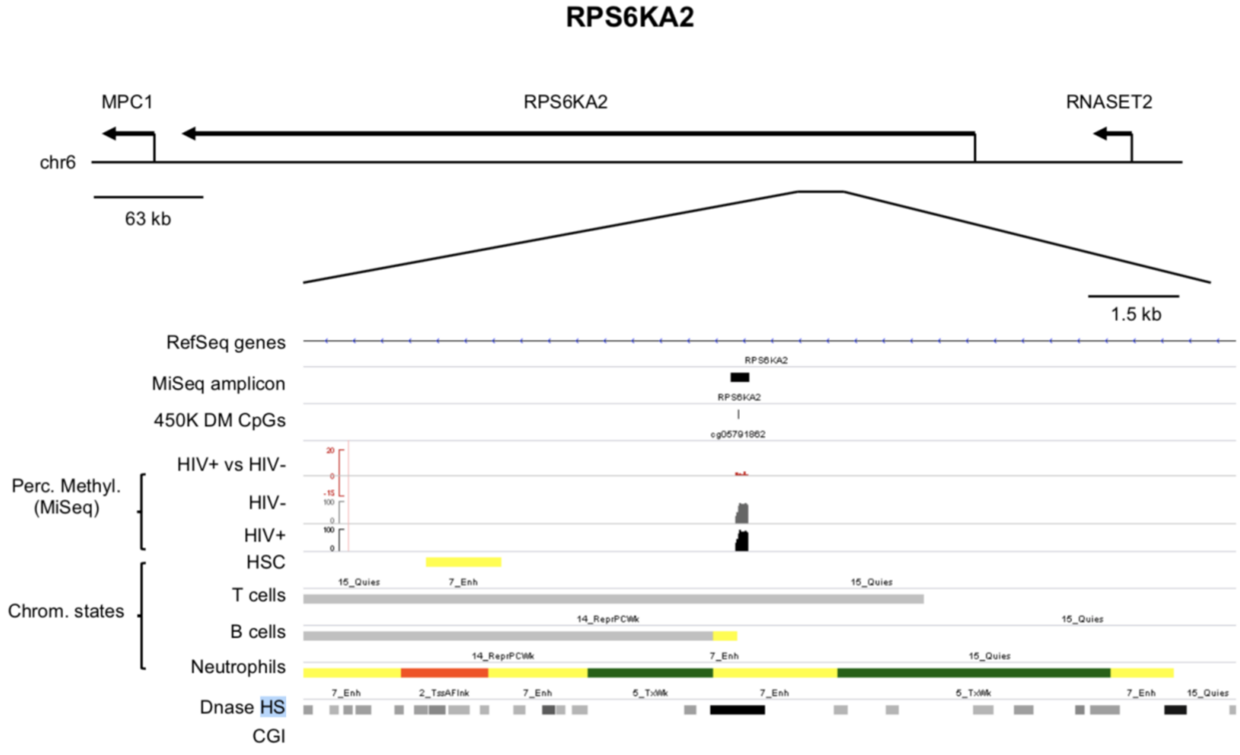

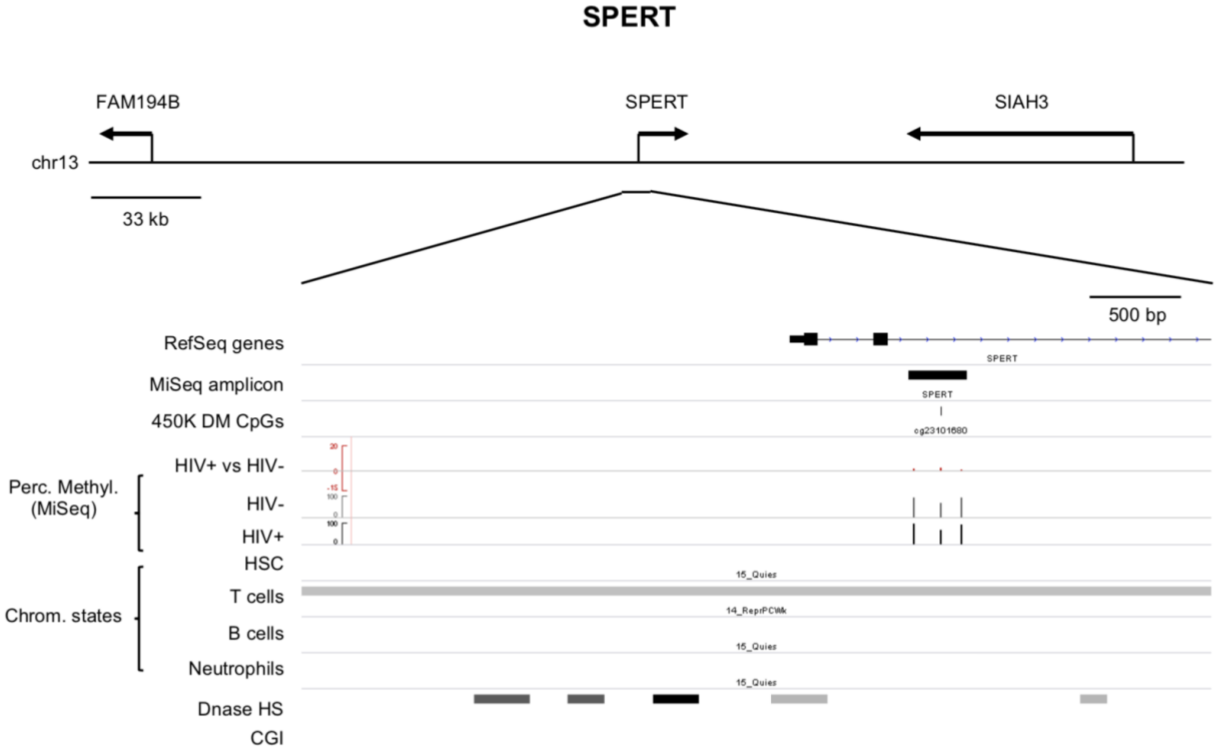

Map showing relevant ENCODE tracks and the amplicons utilized for targeted bisulfite sequencing for amplicons with statistically significant differences between HIV groups

Fig. S5.

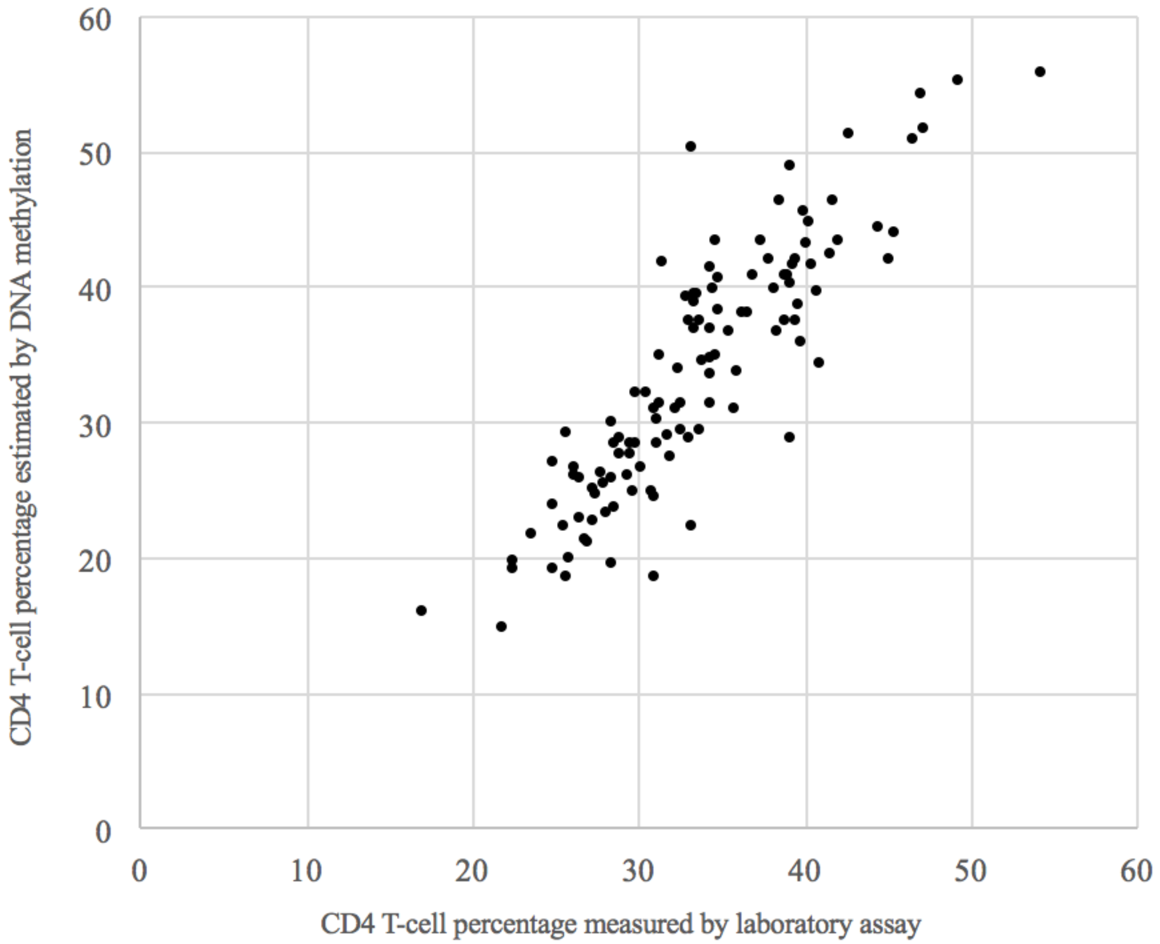

Relationship of $CD4^+$ T-cell percentage of lymphocytes estimated by DNA methylation vs. CD4+ T-cell percentage measured by laboratory assay in samples for 179 HIV-infected children

Table S1. Selected differentially methylated CpG sites (N=1,309) associated with HIV after adjustment for age, sex, and cell type proportion with FDR q-value <0.05 and |Δβ| >0.05, sorted by P-value from smallest to largest

CpG	CHR	Position	Gene	Relation to Gene	Relation to CpG Island	β (HIV+)	β (HIV-)	Δβ	P	Adj P
cg09239591	2	31638511	XDH	TSS1500	OpenSea	0.675	0.623	0.053	2.44E-15	1.97E-11
cg23101680	13	46277286	SPERT	Body	OpenSea	0.717	0.663	0.053	4.89E-14	1.83E-10
cg07839457	16	57023022	NLRC5	TSS1500	N_Shore	0.261	0.353	-0.092	4.69E-11	2.87E-08
cg12027254	17	76055290	TNRC6C	Body	OpenSea	0.824	0.766	0.058	1.00E-10	4.99E-08
cg07970325	6	106497542			OpenSea	0.569	0.512	0.057	1.39E-10	6.38E-08
cg26280998	6	53589544			OpenSea	0.715	0.659	0.056	1.72E-10	7.29E-08
cg12880685	10	120489658	C10orf46	Body	OpenSea	0.428	0.378	0.050	1.85E-10	7.66E-08
cg13316433	5	83140500			OpenSea	0.150	0.207	-0.057	2.28E-10	8.94E-08
cg00390724	3	18484742			N_Shore	0.519	0.456	0.063	2.66E-10	1.01E-07
cg22690339	6	38249061	BTBD9	Body	OpenSea	0.792	0.735	0.058	3.47E-10	1.22E-07
cg05929755	1	110663559			OpenSea	0.398	0.339	0.059	7.36E-10	2.15E-07
cg12021671	15	42423807			OpenSea	0.744	0.690	0.054	1.02E-09	2.75E-07
cg08237220	3	156332967			OpenSea	0.531	0.459	0.072	1.07E-09	2.85E-07
cg05006384	14	95942319	C14orf49	TSS200	OpenSea	0.648	0.595	0.053	1.74E-09	4.09E-07
cg13707760	9	95986383	WNK2	Body	OpenSea	0.509	0.449	0.060	2.03E-09	4.58E-07
cg18394552	5	159428643			OpenSea	0.732	0.650	0.081	2.42E-09	5.17E-07
cg10336193	1	206848389			OpenSea	0.644	0.584	0.060	2.88E-09	5.88E-07
cg22348290	8	72459499			N_Shore	0.767	0.709	0.058	3.22E-09	6.37E-07
cg26898099	5	95192949	C5orf27	Body	OpenSea	0.706	0.650	0.055	6.44E-09	1.07E-06
cg14418857	7	121120431			OpenSea	0.616	0.535	0.080	6.86E-09	1.13E-06
cg22647738	17	34304462	CCL16	3'UTR	OpenSea	0.639	0.561	0.078	8.18E-09	1.29E-06
cg05512157	12	50901878	DIP2B	Body	S_Shelf	0.587	0.533	0.054	8.71E-09	1.35E-06
cg21912162	5	71447818	MAP1B	Body	OpenSea	0.329	0.276	0.053	9.65E-09	1.46E-06
cg23720384	21	23747055			OpenSea	0.665	0.583	0.082	1.10E-08	1.61E-06
cg04961225	12	91332479			OpenSea	0.578	0.515	0.063	1.12E-08	1.63E-06
cg10689689	11	63677197	MARK2	3'UTR	S_Shore	0.685	0.634	0.051	1.42E-08	1.93E-06
cg15826519	1	19808575	CAPZB	Body	N_Shelf	0.796	0.742	0.054	1.49E-08	2.00E-06
cg09083958	12	131516365	GPR133	Body	N_Shore	0.696	0.642	0.054	1.53E-08	2.04E-06
cg02341197	21	34185927	C21orf62	5'UTR;1stExon	OpenSea	0.466	0.408	0.058	1.54E-08	2.05E-06
cg08937102	1	1690389	NADK	Body	N_Shore	0.481	0.428	0.053	1.75E-08	2.24E-06
cg20954180	3	54606265	CACNA2D3	Body	OpenSea	0.847	0.794	0.053	1.75E-08	2.25E-06
cg17069396	20	2731102	EBF4	Body	Island	0.128	0.069	0.058	1.81E-08	2.30E-06
cg16426670	20	2675996	EBF4	Body	S_Shore	0.812	0.750	0.062	2.29E-08	2.74E-06
cg15773890	6	17259549			OpenSea	0.866	0.812	0.054	2.73E-08	3.12E-06
cg03705319	12	12848516	GPR19	5'UTR	N_Shore	0.745	0.693	0.052	2.75E-08	3.14E-06
cg00602295	16	81328089			OpenSea	0.441	0.375	0.065	2.81E-08	3.18E-06
cg17009978	3	183959000	VWA5B2;MIR1224	Body;TSS200	N_Shore	0.719	0.667	0.052	2.84E-08	3.21E-06
cg24399529	15	69373153	MIR548H4;TMEM84	Body;TSS200	OpenSea	0.256	0.205	0.051	2.92E-08	3.28E-06
cg23429698	8	98878169			N_Shelf	0.324	0.268	0.056	3.13E-08	3.44E-06
cg23992886	7	116420651	MET	Body	OpenSea	0.635	0.578	0.058	3.43E-08	3.68E-06
cg00399027	16	85676861	KIAA0182	5'UTR;Body	S_Shore	0.607	0.547	0.060	3.72E-08	3.89E-06
cg04042861	2	231989824	PSMD1;HTR2B	TSS200;Body	OpenSea	0.406	0.346	0.060	4.25E-08	4.29E-06

cg18835596	6	135540107	MYB	3'UTR	OpenSea	0.725	0.659	0.066	4.46E-08	4.45E-06
cg03182584	7	36364854	KIAA0895	3'UTR	OpenSea	0.689	0.631	0.058	4.53E-08	4.49E-06
cg00105306	15	85194978	WDR73	Body	N_Shelf	0.626	0.569	0.057	4.88E-08	4.75E-06
cg24499605	1	3142925	PRDM16	Body	OpenSea	0.550	0.489	0.061	5.85E-08	5.41E-06
cg07589393	7	26984305			OpenSea	0.724	0.674	0.050	6.01E-08	5.52E-06
cg09722609	5	149887422	NDST1	TSS1500	OpenSea	0.607	0.551	0.055	6.05E-08	5.54E-06
cg04339360	13	73635568	KLF5	Body	S_Shore	0.710	0.644	0.066	6.23E-08	5.67E-06
cg20426710	22	50248907	ZBED4	5'UTR	N_Shore	0.519	0.461	0.057	6.33E-08	5.74E-06
cg07264238	17	33474376	UNC45B	TSS1500	OpenSea	0.778	0.707	0.071	7.23E-08	6.32E-06
cg25718604	20	57601474	TUBB1	3'UTR	S_Shelf	0.831	0.774	0.057	7.34E-08	6.40E-06
cg19683780	8	42907576			N_Shelf	0.784	0.733	0.051	7.67E-08	6.60E-06
cg12449049	6	25088999	CMAH	Body	OpenSea	0.686	0.636	0.050	8.32E-08	6.97E-06
cg00864012	17	62294665	TEX2	5'UTR	OpenSea	0.724	0.670	0.053	8.38E-08	7.01E-06
cg00991744	12	41581484	PDZRN4	TSS1500	N_Shore	0.852	0.791	0.061	8.42E-08	7.04E-06
cg09620718	16	20702872	ACSM1	TSS1500	OpenSea	0.784	0.733	0.051	8.51E-08	7.08E-06
cg08066875	2	163197084			N_Shelf	0.637	0.581	0.056	8.63E-08	7.15E-06
cg08324152	19	57172404			N_Shelf	0.283	0.216	0.067	9.18E-08	7.49E-06
cg05825244	20	2730488	EBF4	Body	Island	0.428	0.271	0.157	9.32E-08	7.58E-06
cg00876141	4	47837775	CORIN	Body	N_Shore	0.451	0.396	0.055	9.35E-08	7.59E-06
cg06096336	2	231989800	PSMD1;HTR2B	Body;1stExon;5'UTR	OpenSea	0.571	0.519	0.052	9.83E-08	7.88E-06
cg11329129	11	705692	EPS8L2	TSS1500	N_Shore	0.417	0.367	0.051	9.86E-08	7.89E-06
cg12876838	4	5897223			S_Shelf	0.754	0.702	0.052	1.10E-07	8.58E-06
cg26475911	17	73056187	KCTD2	Body	OpenSea	0.458	0.390	0.067	1.14E-07	8.78E-06
cg13561554	15	78795944			N_Shelf	0.620	0.567	0.053	1.19E-07	9.04E-06
cg26413942	5	124081751	ZNF608	TSS1500	OpenSea	0.421	0.361	0.060	1.24E-07	9.27E-06
cg24263062	20	2730191	EBF4	Body	Island	0.636	0.550	0.086	1.30E-07	9.59E-06
cg13958199	9	127018815	NEK6	TSS1500	N_Shore	0.682	0.631	0.051	1.33E-07	9.77E-06
cg02909097	17	2843206	RAP1GAP2	Body	OpenSea	0.703	0.652	0.050	1.41E-07	1.02E-05
cg16396907	11	114229775			OpenSea	0.229	0.179	0.050	1.50E-07	1.07E-05
cg03477332	5	16178784	MARCH11	Body	N_Shore	0.413	0.360	0.053	1.51E-07	1.07E-05
cg02577773	2	208027870	KLF7	Body	N_Shelf	0.652	0.588	0.064	1.56E-07	1.09E-05
cg12588917	6	29692082	HLA-F	Body	Island	0.395	0.445	-0.051	1.79E-07	1.21E-05
cg00787180	14	91751731	CCDC88C	Body	N_Shelf	0.393	0.340	0.052	1.79E-07	1.21E-05
cg14659930	3	114128301	ZBTB20	5'UTR	OpenSea	0.694	0.626	0.068	1.80E-07	1.21E-05
cg26780125	8	125388806			S_Shelf	0.350	0.300	0.050	1.82E-07	1.22E-05
cg19624420	14	48541885			OpenSea	0.869	0.798	0.071	2.00E-07	1.31E-05
cg04982834	1	93258288	EVI5	TSS1500	OpenSea	0.666	0.609	0.057	2.14E-07	1.38E-05
cg04223006	5	82845599	VCAN	Body	OpenSea	0.433	0.362	0.072	2.23E-07	1.42E-05
cg07995200	6	108865761			OpenSea	0.809	0.759	0.050	2.32E-07	1.46E-05
cg27579313	17	7381622	ZBTB4;ZBTB4	5'UTR	N_Shore	0.559	0.481	0.078	2.49E-07	1.54E-05
cg16565528	8	91010920			N_Shelf	0.835	0.784	0.051	2.73E-07	1.64E-05
cg25879365	1	40510058	CAP1	5'UTR	S_Shelf	0.598	0.517	0.081	2.73E-07	1.64E-05
cg06040034	8	1618448	DLGAP2	Body	S_Shore	0.780	0.719	0.061	2.80E-07	1.67E-05
cg09029192	17	76015204	TNRC6C	5'UTR	OpenSea	0.709	0.654	0.054	3.16E-07	1.84E-05
cg14905634	12	73057369	TRHDE	3'UTR	OpenSea	0.707	0.654	0.053	3.33E-07	1.90E-05
cg13518079	20	2675072	EBF4	Body	S_Shore	0.350	0.262	0.088	3.33E-07	1.90E-05
cg00059652	17	67605498			OpenSea	0.639	0.579	0.061	3.37E-07	1.92E-05
cg25671837	6	127513912	RSPO3	Body	OpenSea	0.437	0.369	0.067	3.53E-07	1.99E-05

cg04873188	2	70143463	MXD1	Body	S_Shore	0.567	0.494	0.073	3.91E-07	2.14E-05
cg25670076	6	90807675	BACH2	5'UTR	OpenSea	0.773	0.708	0.065	4.04E-07	2.19E-05
cg18640098	5	9888660	LOC285692	Body	OpenSea	0.869	0.807	0.063	4.05E-07	2.19E-05
cg02127888	16	1665900	CRAMP1L	Body	S_Shore	0.428	0.346	0.082	4.06E-07	2.20E-05
cg08036553	1	64649805			OpenSea	0.844	0.790	0.055	4.16E-07	2.24E-05
cg11681597	1	249116587	SH3BP5L	Body	N_Shelf	0.507	0.456	0.051	4.45E-07	2.35E-05
cg12039595	1	244480459			OpenSea	0.512	0.455	0.057	4.49E-07	2.36E-05
cg13174253	8	146278678	C8orf33	Body	S_Shore	0.623	0.573	0.050	4.50E-07	2.37E-05
cg22986770	8	61648691	CHD7	5'UTR	OpenSea	0.477	0.404	0.073	4.51E-07	2.37E-05
cg00076653	4	15341878	C1QTNF7	Body	OpenSea	0.527	0.472	0.055	4.66E-07	2.43E-05
cg01232748	4	54928819	CHIC2	Body	N_Shore	0.511	0.451	0.060	4.86E-07	2.50E-05
cg00638210	3	126732518	PLXNA1	Body	N_Shore	0.685	0.630	0.055	5.33E-07	2.67E-05
cg22143698	5	10608058	ANKRD33B	Body	OpenSea	0.199	0.147	0.052	5.35E-07	2.68E-05
cg23824801	12	54653403	CBX5	TSS200;5'UTR	OpenSea	0.280	0.218	0.062	5.41E-07	2.70E-05
cg08173709	3	71103443	FOXP1	Body	OpenSea	0.726	0.669	0.057	5.42E-07	2.71E-05
cg02320356	8	127237106			OpenSea	0.789	0.734	0.055	5.51E-07	2.74E-05
cg08899667	6	31761055	VARS	Body	N_Shelf	0.620	0.568	0.052	5.56E-07	2.76E-05
cg25217710	1	156609523			N_Shelf	0.649	0.591	0.058	5.57E-07	2.76E-05
cg12409074	11	129870648	PRDM10	5'UTR	N_Shore	0.489	0.430	0.060	5.65E-07	2.78E-05
cg12821315	3	183959034	VWA5B2;MIR1224	Body;TSS200	N_Shore	0.822	0.772	0.050	5.97E-07	2.90E-05
cg16356622	1	36351841	EIF2C1	Body	S_Shelf	0.522	0.466	0.056	6.43E-07	3.05E-05
cg10264529	14	24562064	PCK2	TSS1500	N_Shore	0.634	0.573	0.061	6.67E-07	3.13E-05
cg20755748	12	31799116	C12orf72	TSS1500	OpenSea	0.572	0.515	0.057	6.73E-07	3.15E-05
cg02302089	7	23401203	IGF2BP3	Body	OpenSea	0.415	0.354	0.061	6.76E-07	3.15E-05
cg11274172	11	75736215	UVRAG	Body	OpenSea	0.479	0.427	0.052	6.81E-07	3.17E-05
cg22497969	1	3143018	PRDM16	Body	OpenSea	0.784	0.728	0.057	7.34E-07	3.35E-05
cg05236731	13	41035061	LOC646982	Body	OpenSea	0.852	0.797	0.055	8.46E-07	3.72E-05
cg15847685	4	62129666			OpenSea	0.786	0.726	0.060	8.59E-07	3.76E-05
cg07349663	1	160261619	COPA	Body	OpenSea	0.658	0.598	0.061	8.74E-07	3.80E-05
cg14799457	18	19927125			N_Shore	0.317	0.262	0.055	9.46E-07	4.03E-05
cg16312514	11	70650521	SHANK2	Body	OpenSea	0.590	0.533	0.057	9.51E-07	4.04E-05
cg01734045	4	109967535	COL25A1	Body	OpenSea	0.292	0.240	0.052	9.82E-07	4.14E-05
cg20433822	10	124690751	C10orf88	3'UTR	OpenSea	0.278	0.214	0.063	1.09E-06	4.46E-05
cg25199005	1	246446300	SMYD3	Body	OpenSea	0.777	0.713	0.064	1.12E-06	4.56E-05
cg07143462	2	157289513			N_Shelf	0.688	0.622	0.066	1.20E-06	4.78E-05
cg08597292	6	89996028	GABRR2	Body	OpenSea	0.898	0.845	0.053	1.21E-06	4.81E-05
cg20117260	19	41937434	ATP5SL	3'UTR;Body	OpenSea	0.584	0.534	0.050	1.24E-06	4.92E-05
cg00391741	3	127307227	TPRA1	5'UTR	N_Shore	0.735	0.679	0.056	1.25E-06	4.94E-05
cg13448092	3	181135878			OpenSea	0.521	0.471	0.050	1.30E-06	5.05E-05
cg07176842	14	43972223			OpenSea	0.574	0.483	0.092	1.41E-06	5.37E-05
cg00217953	2	97527635	SEMA4C	Body	Island	0.643	0.592	0.050	1.43E-06	5.42E-05
cg26955132	12	13181499			OpenSea	0.744	0.673	0.070	1.46E-06	5.53E-05
cg08090407	7	7139271			OpenSea	0.697	0.640	0.057	1.54E-06	5.73E-05
cg20638127	6	31023286	HCG22	Body	OpenSea	0.807	0.756	0.050	1.57E-06	5.81E-05
cg00500176	19	10396158	ICAM4	TSS1500;3'UTR	N_Shore	0.591	0.537	0.054	1.70E-06	6.15E-05
cg16321846	7	4050114	SDK1	Body	OpenSea	0.751	0.684	0.067	1.71E-06	6.16E-05
cg22859727	14	86090320	FLRT2	3'UTR	OpenSea	0.432	0.380	0.052	1.75E-06	6.28E-05
cg17044311	10	101542983	ABCC2	Body	OpenSea	0.813	0.760	0.053	1.75E-06	6.28E-05

cg21545762	3	183959118	VWA5B2;MIR1224	Body;TSS200	Island	0.562	0.510	0.052	1.79E-06	6.39E-05
cg19956914	7	56147257	SUMF2	Body	OpenSea	0.663	0.610	0.053	1.80E-06	6.42E-05
cg24134261	2	33661362	RASGRP3	TSS200	OpenSea	0.403	0.346	0.057	1.88E-06	6.60E-05
cg13485718	14	93315488			OpenSea	0.560	0.498	0.062	1.89E-06	6.62E-05
cg24897320	1	110042528	CYB561D1	3'UTR	OpenSea	0.545	0.494	0.051	1.89E-06	6.63E-05
cg06121226	4	72134060	SLC4A4	Body	OpenSea	0.829	0.774	0.055	1.92E-06	6.69E-05
cg11945474	2	55717980			OpenSea	0.773	0.711	0.062	2.00E-06	6.89E-05
cg13089335	1	167203243	POU2F1	5'UTR	OpenSea	0.543	0.492	0.052	2.04E-06	7.00E-05
cg14989243	6	76203530	FILIP1	TSS200	OpenSea	0.527	0.452	0.075	2.08E-06	7.10E-05
cg24243879	10	76614628	MYST4	Body	OpenSea	0.260	0.207	0.053	2.13E-06	7.23E-05
cg12081645	4	157920476			OpenSea	0.444	0.375	0.069	2.31E-06	7.68E-05
cg16305292	8	142219965			N_Shore	0.627	0.572	0.055	2.36E-06	7.81E-05
cg02329430	15	73921385	NPTN	Body	N_Shelf	0.391	0.328	0.063	2.50E-06	8.13E-05
cg13717434	9	97854044			OpenSea	0.602	0.545	0.057	2.65E-06	8.50E-05
cg19288514	22	25594918	CRYBB3	TSS1500	OpenSea	0.526	0.473	0.053	2.75E-06	8.71E-05
cg25015371	6	108874950			N_Shelf	0.580	0.527	0.053	2.99E-06	9.26E-05
cg23970645	14	89695299	FOXN3	Body	OpenSea	0.777	0.714	0.063	3.10E-06	9.52E-05
cg26837192	2	48813184	STON1;STON1-GTF2A1L	Body	OpenSea	0.778	0.714	0.063	3.18E-06	9.69E-05
cg07425204	17	66511183	PRKAR1A	5'UTR	S_Shelf	0.481	0.421	0.060	3.26E-06	9.86E-05
cg01282174	11	119630144			OpenSea	0.484	0.415	0.069	3.35E-06	1.01E-04
cg11429292	12	6888035			OpenSea	0.178	0.128	0.050	3.41E-06	1.02E-04
cg08129017	17	17728660	SREBF1	Body	S_Shore	0.412	0.357	0.055	3.61E-06	1.06E-04
cg17082225	7	56673049			OpenSea	0.667	0.613	0.054	3.65E-06	1.07E-04
cg17301248	12	112467203	NAA25	3'UTR	OpenSea	0.642	0.564	0.078	3.66E-06	1.07E-04
cg26943120	4	5472116	STK32B	Body	OpenSea	0.631	0.574	0.057	3.68E-06	1.08E-04
cg12549908	10	4230567			OpenSea	0.761	0.705	0.056	3.80E-06	1.10E-04
cg03820688	2	55477848	MTIF2	Body	OpenSea	0.744	0.683	0.061	4.03E-06	1.15E-04
cg16479633	8	20080666			OpenSea	0.564	0.512	0.052	4.17E-06	1.17E-04
cg24760467	10	102760784	LZTS2	5'UTR	S_Shore	0.622	0.565	0.057	4.18E-06	1.18E-04
cg00583068	1	224972796			OpenSea	0.592	0.542	0.051	4.34E-06	1.21E-04
cg15034267	22	39106452	GTPBP1	Body	S_Shelf	0.768	0.700	0.068	4.36E-06	1.21E-04
cg26959655	8	103379529	UBR5	Body	OpenSea	0.525	0.473	0.052	4.36E-06	1.22E-04
cg07039560	5	140683681	SLC25A2	TSS200	Island	0.667	0.613	0.054	4.43E-06	1.23E-04
cg27416489	2	149823115	KIF5C	Body	OpenSea	0.750	0.686	0.064	4.46E-06	1.23E-04
cg25688583	15	57510460	TCF12	TSS1500;Body	OpenSea	0.674	0.610	0.064	4.48E-06	1.24E-04
cg07793033	16	85256423			OpenSea	0.745	0.695	0.050	4.63E-06	1.27E-04
cg17568809	5	142153529	ARHGAP26	Body	S_Shelf	0.742	0.681	0.061	4.83E-06	1.31E-04
cg01948202	3	122400474	PARP14	Body	S_Shore	0.156	0.211	-0.054	4.85E-06	1.31E-04
cg05264870	2	206599315	NRP2	Body	OpenSea	0.606	0.545	0.061	4.90E-06	1.32E-04
cg07922843	1	92083173			OpenSea	0.705	0.653	0.051	4.95E-06	1.33E-04
cg16153042	6	3282307	SLC22A23	Body	OpenSea	0.646	0.587	0.058	5.15E-06	1.37E-04
cg15059804	1	33766318	ZNF362	3'UTR	OpenSea	0.731	0.649	0.082	5.34E-06	1.41E-04
cg19449565	2	240230892	HDAC4	Body	OpenSea	0.898	0.843	0.055	5.85E-06	1.51E-04
cg16317516	11	47294196	MADD	5'UTR	S_Shelf	0.557	0.498	0.059	5.86E-06	1.51E-04
cg11973132	11	67052656	ADRBK1	Body	Island	0.584	0.534	0.051	5.96E-06	1.53E-04
cg06899237	6	153353519	RGS17	Body	OpenSea	0.427	0.371	0.056	6.22E-06	1.57E-04
cg06832406	10	4230482			OpenSea	0.810	0.758	0.052	6.22E-06	1.57E-04
cg04083076	1	169452126	SLC19A2	Body	N_Shelf	0.697	0.633	0.065	6.23E-06	1.57E-04

cg05475386	12	65723693	MSRB3	Body	OpenSea	0.304	0.243	0.061	6.25E-06	1.58E-04
cg22491058	1	207277466	C4BPA	TSS200	OpenSea	0.502	0.448	0.054	6.38E-06	1.60E-04
cg01123957	14	34081647	NPAS3	Body	OpenSea	0.286	0.234	0.052	6.57E-06	1.64E-04
cg00950497	10	116393423	ABLIM1	Body	S_Shore	0.630	0.569	0.062	6.73E-06	1.67E-04
cg17111025	2	111936357			OpenSea	0.944	0.887	0.056	6.87E-06	1.69E-04
cg05857996	20	2675418	EBF4	Body	S_Shore	0.786	0.686	0.101	6.88E-06	1.69E-04
cg14289429	9	134139878	FAM78A	Body	S_Shelf	0.440	0.388	0.053	6.90E-06	1.70E-04
cg13547053	2	179184864	OSBPL6	TSS200;Body	OpenSea	0.717	0.659	0.058	7.13E-06	1.74E-04
cg10897223	6	30113774	TRIM40	Body	OpenSea	0.666	0.613	0.053	7.48E-06	1.79E-04
cg03529358	17	78198361	SLC26A11	Body	S_Shelf	0.935	0.875	0.060	7.78E-06	1.85E-04
cg06071730	10	112021300	MXI1	Body	OpenSea	0.714	0.658	0.055	7.87E-06	1.87E-04
cg10366878	4	79531297	ANXA3	3'UTR	OpenSea	0.657	0.594	0.063	7.98E-06	1.88E-04
cg05684528	5	76473917			N_Shelf	0.636	0.577	0.060	8.11E-06	1.90E-04
cg10437839	11	64701910	PPP2R5B	3'UTR	OpenSea	0.657	0.604	0.052	8.11E-06	1.91E-04
cg18808261	3	18464935	SATB1;SATB1	5'UTR	N_Shore	0.290	0.231	0.059	8.33E-06	1.94E-04
cg08340023	16	23098187	USP31	Body	OpenSea	0.689	0.623	0.066	8.33E-06	1.94E-04
cg02220129	11	58598239			OpenSea	0.837	0.774	0.064	8.50E-06	1.97E-04
cg08602008	19	48076841			Island	0.337	0.284	0.053	8.80E-06	2.02E-04
cg19662895	14	69074455			OpenSea	0.398	0.346	0.052	9.06E-06	2.07E-04
cg02061820	3	152046751	MBNL1	Body	OpenSea	0.459	0.385	0.074	9.36E-06	2.12E-04
cg01618151	6	35707957	C6orf81	Body	OpenSea	0.512	0.461	0.050	9.55E-06	2.15E-04
cg27192248	15	65285669			S_Shelf	0.409	0.517	-0.108	9.67E-06	2.17E-04
cg07661704	4	139144433	SLC7A11	Body	OpenSea	0.561	0.496	0.065	9.74E-06	2.18E-04
cg15644413	1	36185939	C1orf216	TSS1500	S_Shore	0.699	0.643	0.056	9.98E-06	2.22E-04
cg26235748	14	86087511	FLRT2	5'UTR	OpenSea	0.531	0.454	0.077	1.01E-05	2.23E-04
cg10902738	8	134895500			OpenSea	0.667	0.605	0.062	1.04E-05	2.29E-04
cg07291349	4	40964962	APBB2	Body	OpenSea	0.643	0.572	0.071	1.07E-05	2.33E-04
cg04139359	1	45085438	RNF220	Body	S_Shelf	0.808	0.747	0.062	1.07E-05	2.33E-04
cg03819286	16	4673974	MGRN1	TSS1500	N_Shore	0.725	0.674	0.051	1.07E-05	2.33E-04
cg03546806	12	114297307	RBM19	Body	OpenSea	0.740	0.689	0.051	1.15E-05	2.45E-04
cg02059519	9	137250935	RXRA	Body	N_Shore	0.797	0.741	0.056	1.17E-05	2.49E-04
cg01621943	17	53531409			OpenSea	0.697	0.646	0.051	1.17E-05	2.49E-04
cg10196532	13	50134640	RCBTB1	Body	OpenSea	0.818	0.734	0.084	1.18E-05	2.50E-04
cg26293423	5	71613149	MRPS27	Body	N_Shelf	0.749	0.695	0.054	1.19E-05	2.52E-04
cg00021275	2	128142155			N_Shelf	0.731	0.674	0.057	1.22E-05	2.56E-04
cg20141108	1	165907859			N_Shelf	0.653	0.587	0.066	1.22E-05	2.57E-04
cg27408285	12	54653364	CBX5	1stExon;TSS200;5'UTR	OpenSea	0.331	0.265	0.066	1.23E-05	2.58E-04
cg11165912	4	110220063	COL25A1	Body	N_Shelf	0.258	0.207	0.051	1.25E-05	2.61E-04
cg05708073	1	109637162	TMEM167B	3'UTR	S_Shelf	0.745	0.690	0.056	1.26E-05	2.62E-04
cg14342532	2	233916534			OpenSea	0.355	0.299	0.056	1.28E-05	2.65E-04
cg07624918	3	136533970			N_Shelf	0.752	0.699	0.053	1.31E-05	2.69E-04
cg24235882	4	54928822	CHIC2	Body	N_Shore	0.572	0.518	0.055	1.32E-05	2.70E-04
cg20211629	18	48352490	MRO	TSS1500	OpenSea	0.585	0.531	0.054	1.33E-05	2.73E-04
cg20675439	6	82466362			S_Shelf	0.695	0.636	0.059	1.34E-05	2.73E-04
cg18470427	17	33842301	SLFN12L	Body	OpenSea	0.840	0.768	0.073	1.37E-05	2.78E-04
cg19841423	20	62366755	ZGPAT;LIME1	Body;TSS1500	S_Shore	0.713	0.660	0.053	1.38E-05	2.80E-04
cg22992279	4	26414901	RBPJ	Body	OpenSea	0.745	0.691	0.054	1.39E-05	2.81E-04
cg05808246	1	221741553			OpenSea	0.799	0.740	0.059	1.43E-05	2.88E-04

cg10440877	2	208378475			OpenSea	0.723	0.666	0.057	1.47E-05	2.93E-04
cg08106319	6	37849427	ZFAND3	Body	OpenSea	0.381	0.309	0.073	1.50E-05	2.96E-04
cg01822050	20	304156			N_Shore	0.748	0.686	0.062	1.50E-05	2.97E-04
cg20790648	3	151619923			OpenSea	0.447	0.394	0.053	1.51E-05	2.98E-04
cg16778107	16	381873	AXIN1	Body	OpenSea	0.511	0.451	0.059	1.52E-05	2.99E-04
cg05739816	6	135989395	C6orf217	Body	OpenSea	0.534	0.459	0.074	1.53E-05	3.01E-04
cg11952493	10	28570835	MPP7	5'UTR	OpenSea	0.744	0.693	0.051	1.58E-05	3.09E-04
cg14819618	8	8180214	PRAGMIN	Body	S_Shelf	0.793	0.729	0.063	1.67E-05	3.20E-04
cg11356156	19	55416788	NCR1	TSS1500	OpenSea	0.727	0.677	0.050	1.69E-05	3.24E-04
cg02543850	11	33346306	HIPK3	Body	OpenSea	0.595	0.539	0.057	1.70E-05	3.25E-04
cg00204748	12	29376779	FAR2	1stExon;5'UTR	OpenSea	0.707	0.656	0.052	1.71E-05	3.26E-04
cg00569896	4	204382			N_Shore	0.761	0.705	0.056	1.76E-05	3.34E-04
cg18784409	11	67868331	CHKA	Body	OpenSea	0.509	0.456	0.053	1.78E-05	3.36E-04
cg05850338	17	79933123			N_Shelf	0.686	0.623	0.062	1.78E-05	3.36E-04
cg19995856	14	91574593			OpenSea	0.520	0.440	0.080	1.79E-05	3.38E-04
cg03319082	16	1295309			OpenSea	0.713	0.662	0.050	1.83E-05	3.43E-04
cg24786658	7	112727423	GPR85	TSS1500;5'UTR	OpenSea	0.705	0.647	0.058	1.90E-05	3.52E-04
cg17781958	17	79428404	BAHCC1	Body	Island	0.758	0.702	0.057	1.93E-05	3.56E-04
cg15931921	6	170597588	DLL1	Body	N_Shore	0.748	0.669	0.080	1.95E-05	3.59E-04
cg22143285	17	68070411	KCNJ16	TSS1500	OpenSea	0.550	0.498	0.052	2.00E-05	3.65E-04
cg03680873	3	148844300			N_Shelf	0.600	0.535	0.065	2.04E-05	3.71E-04
cg20176142	8	81408357	ZBTB10	Body	OpenSea	0.728	0.678	0.050	2.11E-05	3.79E-04
cg12114888	8	107738435	OXR1	Body	OpenSea	0.336	0.283	0.053	2.28E-05	4.02E-04
cg14384158	9	140348828	NELF	Body	N_Shore	0.160	0.107	0.053	2.33E-05	4.08E-04
cg24392970	13	86373600	SLITRK6	TSS200	OpenSea	0.539	0.481	0.058	2.33E-05	4.08E-04
cg06857116	17	15885326	ZSWIM7	Body	OpenSea	0.594	0.540	0.054	2.36E-05	4.11E-04
cg24247537	11	457278	PTDSS2	Body	N_Shore	0.230	0.179	0.050	2.37E-05	4.12E-04
cg16674514	17	42145043	LSM12	TSS200	S_Shore	0.748	0.695	0.053	2.38E-05	4.13E-04
cg18638434	14	74486651	C14orf45;ENTPD5	Body;TSS1500	S_Shore	0.732	0.670	0.063	2.39E-05	4.14E-04
cg21636683	5	140683699	SLC25A2	TSS200	Island	0.834	0.781	0.053	2.41E-05	4.17E-04
cg03291835	13	110802745	COL4A1	Body	OpenSea	0.681	0.618	0.064	2.47E-05	4.25E-04
cg11909574	11	58598338			OpenSea	0.694	0.641	0.054	2.53E-05	4.31E-04
cg26136772	7	4754681	FOXK1	Body	N_Shelf	0.708	0.647	0.061	2.54E-05	4.33E-04
cg11367259	8	89969235			OpenSea	0.828	0.773	0.055	2.56E-05	4.35E-04
cg04567302	6	31846956	SLC44A4	TSS200	OpenSea	0.789	0.730	0.059	2.60E-05	4.40E-04
cg09754197	6	7113738	RREB1	5'UTR	S_Shelf	0.741	0.690	0.051	2.60E-05	4.40E-04
cg24023489	8	103129227	NCALD	5'UTR	OpenSea	0.723	0.673	0.051	2.67E-05	4.48E-04
cg00084338	6	170595920	DLL1	Body	N_Shore	0.598	0.495	0.103	2.67E-05	4.49E-04
cg18388786	10	134924050	GPR123	Body	N_Shore	0.312	0.254	0.058	2.70E-05	4.51E-04
cg27246129	6	170595947	DLL1	Body	N_Shore	0.550	0.480	0.070	2.71E-05	4.52E-04
cg08500171	6	31590674	BAT2;SNORA38	Body;TSS200	S_Shore	0.782	0.732	0.050	2.73E-05	4.56E-04
cg02354658	5	95151391	GLRX	3'UTR	OpenSea	0.318	0.267	0.050	2.75E-05	4.57E-04
cg14628914	6	24659344	TTRAP	Body	OpenSea	0.661	0.609	0.052	2.76E-05	4.59E-04
cg11254053	1	33497125	AK2;AK2	Body	OpenSea	0.554	0.496	0.058	2.77E-05	4.59E-04
cg00634542	2	219254588	SLC11A1	Body	N_Shore	0.556	0.495	0.062	2.85E-05	4.70E-04
cg19723805	5	33147539			OpenSea	0.452	0.396	0.055	2.92E-05	4.77E-04
cg20438472	3	184293365	EPHB3	Body	S_Shelf	0.588	0.538	0.050	3.00E-05	4.87E-04
cg05142445	14	76309157	TTLL5	Body	OpenSea	0.716	0.657	0.059	3.04E-05	4.92E-04

cg12559197	5	76654783	PDE8B	Body	OpenSea	0.515	0.440	0.074	3.21E-05	5.10E-04
cg11505048	1	183622726	RGL1;APOBEC4	5'UTR;TSS1500	OpenSea	0.766	0.715	0.051	3.23E-05	5.13E-04
cg07539709	17	80545454	FOXK2	Body	Island	0.598	0.538	0.060	3.24E-05	5.15E-04
cg14324502	9	88898587	ISCA1	TSS1500	S_Shore	0.555	0.489	0.066	3.31E-05	5.23E-04
cg12700074	6	131571435	AKAP7	TSS200;Body	OpenSea	0.180	0.127	0.053	3.32E-05	5.24E-04
cg26423322	2	33905480			OpenSea	0.539	0.487	0.053	3.46E-05	5.39E-04
cg13573513	12	14996143	ART4	5'UTR;1stExon	OpenSea	0.763	0.698	0.065	3.49E-05	5.42E-04
cg18067134	3	71084634	FOXP1	Body	OpenSea	0.728	0.667	0.061	3.56E-05	5.50E-04
cg01634388	4	129405035			OpenSea	0.301	0.238	0.063	3.57E-05	5.51E-04
cg08632701	21	37451849			OpenSea	0.860	0.912	-0.052	3.59E-05	5.54E-04
cg03529189	12	64292138	SRGAP1	Body	OpenSea	0.743	0.689	0.053	3.60E-05	5.55E-04
cg23711422	21	43442085	C21orf121	TSS200	OpenSea	0.502	0.430	0.073	3.66E-05	5.62E-04
cg08739755	2	172815385	HAT1	Body	OpenSea	0.803	0.737	0.066	3.82E-05	5.78E-04
cg19560014	12	31799118	C12orf72	TSS1500	OpenSea	0.614	0.554	0.061	3.86E-05	5.83E-04
cg16901361	8	107526473	OXR1	Body	OpenSea	0.253	0.201	0.052	3.87E-05	5.84E-04
cg05071334	8	86195487	CA13	3'UTR	OpenSea	0.643	0.589	0.054	3.88E-05	5.85E-04
cg08461760	7	25703644			OpenSea	0.790	0.736	0.054	3.94E-05	5.91E-04
cg10584024	1	84234998			OpenSea	0.541	0.469	0.072	3.95E-05	5.92E-04
cg04098052	7	24517414			OpenSea	0.674	0.620	0.053	3.95E-05	5.92E-04
cg15516558	1	161091980	DEDD	Body	S_Shelf	0.488	0.434	0.054	3.98E-05	5.95E-04
cg06508738	3	138763852	PRR23C	TSS200	Island	0.623	0.567	0.057	3.98E-05	5.95E-04
cg07951201	13	103340215	C13orf39	Body	OpenSea	0.444	0.378	0.065	4.01E-05	5.98E-04
cg12647970	6	3234297			S_Shelf	0.805	0.750	0.055	4.23E-05	6.22E-04
cg05233670	12	109058033	CORO1C	Body	OpenSea	0.479	0.415	0.064	4.24E-05	6.23E-04
cg08707875	1	224575077	WDR26	3'UTR	OpenSea	0.702	0.646	0.056	4.31E-05	6.31E-04
cg21781157	20	47874111	ZNFX1	Body	OpenSea	0.530	0.468	0.063	4.37E-05	6.37E-04
cg17846127	15	89940505	LOC254559	Body	N_Shelf	0.688	0.632	0.056	4.39E-05	6.38E-04
cg06663615	17	79423959	BAHCC1	Body	N_Shore	0.565	0.513	0.052	4.42E-05	6.42E-04
cg21043558	7	140625365	BRAF	TSS1500	S_Shore	0.717	0.663	0.054	4.53E-05	6.53E-04
cg05791862	6	167189213	RPS6KA2	Body	OpenSea	0.734	0.683	0.051	4.65E-05	6.64E-04
cg03077077	4	122685352	TMEM155;LOC100192379	5'UTR;TSS1500	N_Shore	0.684	0.624	0.060	4.66E-05	6.66E-04
cg06633438	19	6272158	MLLT1	Body	Island	0.606	0.525	0.080	4.67E-05	6.66E-04
cg22596016	17	61711557	MAP3K3	Body	OpenSea	0.266	0.201	0.065	4.82E-05	6.82E-04
cg17129188	8	134307597	NDRG1	5'UTR	N_Shore	0.774	0.722	0.052	4.82E-05	6.82E-04
cg18115721	11	73567838	MRPL48	Body	OpenSea	0.807	0.751	0.056	5.02E-05	7.01E-04
cg02112168	14	45579561	PRPF39;SNORD127	Body;TSS1500	OpenSea	0.663	0.592	0.070	5.06E-05	7.06E-04
cg27134944	13	76111107	COMMD6	Body	N_Shore	0.560	0.501	0.059	5.10E-05	7.09E-04
cg08064068	14	60671024			OpenSea	0.663	0.603	0.060	5.15E-05	7.14E-04
cg00808648	14	105779910	PACS2;PACS2	TSS1500	N_Shore	0.307	0.256	0.051	5.34E-05	7.33E-04
cg24530147	3	138763894	PRR23C	TSS200	Island	0.773	0.707	0.066	5.35E-05	7.34E-04
cg19161559	7	101465759	CUX1	Body	OpenSea	0.720	0.669	0.052	5.42E-05	7.40E-04
cg26104475	5	169693176	LCP2	Body	OpenSea	0.640	0.584	0.056	5.45E-05	7.43E-04
cg25800638	2	24527092	ITSN2	Body	OpenSea	0.741	0.688	0.053	5.46E-05	7.44E-04
cg09777883	11	112093696			N_Shelf	0.744	0.688	0.056	5.51E-05	7.50E-04
cg07769015	8	142238770	SLC45A4	TSS200	S_Shore	0.624	0.568	0.057	5.55E-05	7.53E-04
cg02065051	20	25674725	ZNF337	5'UTR	N_Shore	0.480	0.426	0.054	5.56E-05	7.54E-04
cg13607230	2	103352296	MFSD9	Body	N_Shore	0.473	0.404	0.069	5.56E-05	7.55E-04
cg08481967	6	166765599			OpenSea	0.358	0.272	0.086	5.59E-05	7.57E-04

cg17510957	11	121466629	SORL1	Body	OpenSea	0.700	0.637	0.063	5.61E-05	7.59E-04
cg22249612	12	56121485	CD63	Body	N_Shore	0.659	0.589	0.069	5.65E-05	7.63E-04
cg14210119	2	182319707			N_Shelf	0.561	0.504	0.058	5.71E-05	7.68E-04
cg20925233	17	47661136	NXPH3	3'UTR	OpenSea	0.563	0.505	0.059	5.84E-05	7.81E-04
cg17164954	6	157345266	ARID1B	Body	S_Shelf	0.457	0.396	0.061	6.06E-05	8.02E-04
cg16805291	7	36022575			Island	0.692	0.623	0.069	6.14E-05	8.08E-04
cg06190046	16	83986382	OSGIN1	5'UTR;TSS1500;Body	OpenSea	0.500	0.427	0.074	6.26E-05	8.20E-04
cg06487194	6	15345097	JARID2	Body	OpenSea	0.589	0.525	0.064	6.27E-05	8.21E-04
cg20043649	2	47537239			OpenSea	0.699	0.636	0.064	6.60E-05	8.50E-04
cg10601026	10	64579140	EGR2	TSS1500	S_Shore	0.517	0.464	0.052	6.65E-05	8.55E-04
cg14044785	2	43560368	THADA;THADA	Body	OpenSea	0.692	0.623	0.069	6.70E-05	8.60E-04
cg16702362	2	135078044	MGAT5	Body	OpenSea	0.719	0.654	0.064	6.94E-05	8.82E-04
cg05407338	17	8213522	ARHGEF15	TSS200	OpenSea	0.484	0.429	0.055	6.95E-05	8.83E-04
cg24165638	19	831456	AZU1	Body	N_Shelf	0.640	0.586	0.054	7.00E-05	8.88E-04
cg09803321	10	104913480	NT5C2	Body	OpenSea	0.727	0.670	0.057	7.10E-05	8.96E-04
cg05217983	6	45406867	RUNX2	Body	OpenSea	0.350	0.291	0.060	7.39E-05	9.23E-04
cg07330114	3	11624023	VGLL4	Body;TSS200	OpenSea	0.238	0.177	0.060	7.50E-05	9.33E-04
cg14179944	14	35867515			OpenSea	0.471	0.421	0.050	7.52E-05	9.34E-04
cg10805254	3	72433837	RYBP	Body	OpenSea	0.581	0.518	0.063	7.58E-05	9.38E-04
cg26698460	19	58716004	ZNF274	Body;5'UTR	Island	0.641	0.588	0.053	7.63E-05	9.44E-04
cg02209770	20	35062903	DLGAP4	Body	N_Shore	0.553	0.502	0.051	7.80E-05	9.58E-04
cg05687149	11	112035945	IL18	TSS1500	OpenSea	0.278	0.227	0.052	7.82E-05	9.60E-04
cg27416337	3	32748004	CNOT10	Body	OpenSea	0.343	0.280	0.063	8.04E-05	9.79E-04
cg11562411	6	35206618	SCUBE3	Body	OpenSea	0.522	0.471	0.051	8.06E-05	9.80E-04
cg03305017	7	151036715			N_Shelf	0.494	0.434	0.060	8.07E-05	9.81E-04
cg25263801	7	112727506	GPR85	TSS1500;1stExon;5'UTR	OpenSea	0.719	0.664	0.055	8.11E-05	9.85E-04
cg01290904	4	5708474	EVC2	5'UTR;Body	N_Shore	0.629	0.577	0.052	8.18E-05	9.91E-04
cg16616918	17	1686627	SMYD4	Body	OpenSea	0.692	0.635	0.056	8.23E-05	9.95E-04
cg20663365	2	127729097			N_Shore	0.836	0.783	0.053	8.52E-05	1.02E-03
cg07772516	15	52107742	TMOD2	3'UTR	OpenSea	0.611	0.537	0.074	8.54E-05	1.02E-03
cg13127231	13	111806949	ARHGEF7	Body;5'UTR	S_Shore	0.459	0.409	0.050	8.59E-05	1.03E-03
cg21579539	18	43751229			N_Shelf	0.709	0.656	0.052	8.78E-05	1.04E-03
cg02512559	16	75414554	CFDP1	Body	OpenSea	0.240	0.179	0.061	8.79E-05	1.04E-03
cg25757140	19	38039469			N_Shore	0.611	0.553	0.058	9.06E-05	1.07E-03
cg18169971	5	134702492	H2AFY	Body	OpenSea	0.815	0.764	0.051	9.08E-05	1.07E-03
cg11210069	5	140761259	PCDHGA4;PCDHGA6;PCDHGA1;<br>PCDHGA5;PCDHGB1;PCDHGA3;<br>PCDHGA2;PCDHGB2;PCDHGA7;<br>PCDHGA7;PCDHGB3	Body;TSS1500	N_Shore	0.699	0.641	0.058	9.15E-05	1.07E-03
cg05581469	12	49413435	MLL2;PRKAG1	3'UTR;TSS1500	S_Shore	0.368	0.313	0.055	9.21E-05	1.08E-03
cg09305680	8	117778069	UTP23	TSS1500	N_Shore	0.600	0.545	0.055	9.26E-05	1.08E-03
cg02134705	3	149388151	WWTR1	5'UTR	OpenSea	0.849	0.798	0.051	9.26E-05	1.08E-03
cg10976975	2	69098569	BMP10;BMP10	5'UTR;1stExon	OpenSea	0.714	0.649	0.065	9.62E-05	1.11E-03
cg23679982	17	1686737	SMYD4	Body	OpenSea	0.636	0.570	0.066	9.76E-05	1.12E-03
cg02363526	7	150674012	KCNH2	Body	N_Shore	0.552	0.502	0.051	9.83E-05	1.13E-03
cg07572909	1	113467640	SLC16A1	Body	OpenSea	0.648	0.596	0.051	1.00E-04	1.14E-03
cg24926791	6	31082187	PSORS1C1	TSS1500	OpenSea	0.653	0.483	0.170	1.01E-04	1.15E-03
cg14495033	9	140707378	EHMT1	Body	N_Shore	0.714	0.657	0.057	1.01E-04	1.15E-03

cg06865642	22	50174028	BRD1;LOC90834	Body;TSS200	N_Shelf	0.637	0.586	0.051	1.03E-04	1.16E-03
cg03885343	5	98108198	RGMB	Body	N_Shore	0.675	0.624	0.050	1.03E-04	1.17E-03
cg02391509	3	27766382			S_Shore	0.304	0.253	0.052	1.03E-04	1.17E-03
cg00791854	1	154392070	IL6R;IL6R	Body	OpenSea	0.345	0.295	0.050	1.04E-04	1.17E-03
cg06705237	9	97402555	FBP1;FBP1	TSS200;TSS1500	S_Shore	0.636	0.584	0.052	1.05E-04	1.18E-03
cg05916684	17	49008322			N_Shore	0.503	0.444	0.059	1.05E-04	1.18E-03
cg21452188	1	156095914	LMNA	Body	OpenSea	0.472	0.422	0.050	1.06E-04	1.19E-03
cg24590430	10	99097076			S_Shelf	0.792	0.718	0.074	1.06E-04	1.19E-03
cg21697512	1	208081541	CD34	Body	N_Shelf	0.575	0.515	0.060	1.07E-04	1.20E-03
cg01238669	7	21797276	DNAH11	Body	OpenSea	0.446	0.386	0.060	1.09E-04	1.21E-03
cg23098068	2	172650722	SLC25A12	Body	OpenSea	0.420	0.343	0.077	1.15E-04	1.26E-03
cg00522048	10	121172431	GRK5	Body	OpenSea	0.640	0.590	0.050	1.16E-04	1.26E-03
cg06740950	3	171878318	FNDC3B	Body	OpenSea	0.473	0.416	0.057	1.19E-04	1.29E-03
cg08131499	12	109090588	CORO1C	Body	OpenSea	0.308	0.247	0.061	1.20E-04	1.29E-03
cg25357825	3	11697138	VGLL4	Body	OpenSea	0.344	0.294	0.051	1.20E-04	1.30E-03
cg02291010	5	96079433	CAST	Body;1stExon	OpenSea	0.669	0.612	0.057	1.21E-04	1.30E-03
cg23735745	1	230248121	GALNT2	Body	OpenSea	0.304	0.249	0.055	1.21E-04	1.30E-03
cg10789050	3	31713847	OSBPL10	Body	OpenSea	0.509	0.425	0.084	1.21E-04	1.30E-03
cg25723459	11	126245829	ST3GAL4	5'UTR	OpenSea	0.678	0.619	0.059	1.21E-04	1.30E-03
cg17797863	4	2009396	WHSC2	Body	N_Shore	0.542	0.489	0.052	1.21E-04	1.31E-03
cg24506197	7	158201392	PTPRN2	Body	S_Shelf	0.308	0.249	0.058	1.25E-04	1.33E-03
cg18071202	12	1609470	LOC100292680	TSS200	OpenSea	0.552	0.499	0.053	1.26E-04	1.34E-03
cg02457461	8	9913680	MSRA	Body	S_Shore	0.251	0.193	0.058	1.27E-04	1.34E-03
cg06392753	2	102783282	IL1R1	Body	OpenSea	0.549	0.488	0.061	1.29E-04	1.36E-03
cg19947463	7	1113237	C7orf50	Body	OpenSea	0.459	0.384	0.075	1.30E-04	1.37E-03
cg00225858	16	84223841	ADAD2	TSS1500	N_Shore	0.620	0.569	0.051	1.33E-04	1.39E-03
cg24265969	3	55654481	ERC2	3'UTR	OpenSea	0.322	0.262	0.060	1.33E-04	1.39E-03
cg02769705	1	202975059	TMEM183B;TMEM183A	TSS1500	N_Shore	0.588	0.520	0.069	1.35E-04	1.41E-03
cg27301230	7	65761563	TPST1	Body	OpenSea	0.737	0.682	0.055	1.35E-04	1.41E-03
cg18281939	5	77783895	LHFPL2	3'UTR	OpenSea	0.452	0.390	0.062	1.38E-04	1.43E-03
cg02112621	3	78387362			OpenSea	0.640	0.589	0.050	1.39E-04	1.43E-03
cg16745596	19	39695801	SYCN	TSS1500	S_Shore	0.552	0.499	0.052	1.40E-04	1.44E-03
cg06794244	2	196434818			OpenSea	0.470	0.416	0.054	1.40E-04	1.44E-03
cg08915824	1	204911863	NFASC	TSS1500;5'UTR	OpenSea	0.451	0.397	0.055	1.41E-04	1.45E-03
cg21932231	2	36668864	CRIM1	Body	OpenSea	0.543	0.488	0.055	1.43E-04	1.47E-03
cg00819078	5	16615081	FAM134B	Body	N_Shore	0.686	0.627	0.059	1.48E-04	1.50E-03
cg01014262	16	48533930			S_Shelf	0.354	0.294	0.060	1.49E-04	1.51E-03
cg14989033	1	215179151	KCNK2	1stExon;5'UTR	OpenSea	0.463	0.410	0.052	1.51E-04	1.52E-03
cg24238409	10	93998677	CPEB3	Body	N_Shore	0.759	0.704	0.055	1.51E-04	1.52E-03
cg01966334	2	128378434	MYO7B	Body	N_Shelf	0.579	0.523	0.056	1.51E-04	1.52E-03
cg05141432	3	81812214	GBE1	TSS1500	S_Shore	0.361	0.309	0.052	1.53E-04	1.54E-03
cg04100124	10	69611755			S_Shelf	0.531	0.480	0.052	1.53E-04	1.54E-03
cg21252047	9	116419843			OpenSea	0.587	0.535	0.052	1.53E-04	1.54E-03
cg11071448	1	202584465	SYT2;SYT2	5'UTR	OpenSea	0.581	0.530	0.051	1.55E-04	1.55E-03
cg00796963	6	84936558	KIAA1009	5'UTR	N_Shore	0.518	0.463	0.055	1.61E-04	1.59E-03
cg21487561	2	11990393			OpenSea	0.916	0.850	0.066	1.62E-04	1.60E-03
cg23362032	7	126990845			S_Shelf	0.770	0.717	0.052	1.62E-04	1.60E-03
cg27586596	7	41426609			OpenSea	0.444	0.390	0.054	1.64E-04	1.61E-03

cg03854238	6	22071933	FLJ22536	Body	OpenSea	0.619	0.554	0.065	1.65E-04	1.62E-03
cg25287268	13	50694004	DLEU2	Body	N_Shelf	0.439	0.379	0.060	1.66E-04	1.63E-03
cg01550445	11	72929983	P2RY2	5'UTR	S_Shore	0.633	0.581	0.052	1.67E-04	1.63E-03
cg13643040	9	132456332	PRRX2	Body	N_Shelf	0.943	0.852	0.091	1.69E-04	1.65E-03
cg25731553	15	101715276			OpenSea	0.673	0.620	0.054	1.71E-04	1.66E-03
cg21203569	11	47279365	NR1H3	TSS200;TSS200;5'UTR	OpenSea	0.496	0.431	0.065	1.72E-04	1.67E-03
cg25791430	11	59574962	MRPL16	Body	N_Shelf	0.677	0.627	0.050	1.75E-04	1.69E-03
cg06838283	5	111090142	C5orf13	Body	N_Shelf	0.471	0.412	0.059	1.76E-04	1.69E-03
cg18011273	4	7203326	SORCS2	Body	OpenSea	0.665	0.611	0.054	1.78E-04	1.71E-03
cg08151370	3	43073152	FAM198A	Body	OpenSea	0.693	0.623	0.069	1.81E-04	1.73E-03
cg25589001	10	98605052	LCOR	5'UTR	OpenSea	0.557	0.501	0.055	1.81E-04	1.73E-03
cg24159514	19	11785248	ZNF833	Body	Island	0.454	0.378	0.076	1.84E-04	1.74E-03
cg00421164	6	7111068	RREB1	5'UTR	S_Shore	0.827	0.774	0.053	1.90E-04	1.79E-03
cg10368935	10	18240316	SLC39A12;SLC39A12	TSS1500	OpenSea	0.733	0.666	0.066	1.97E-04	1.83E-03
cg15939920	17	35457385	ACACA	Body	OpenSea	0.543	0.491	0.052	2.01E-04	1.86E-03
cg14325153	3	138763899	PRR23C	TSS200	Island	0.537	0.470	0.067	2.01E-04	1.86E-03
cg19619014	8	37250930			OpenSea	0.392	0.331	0.061	2.04E-04	1.88E-03
cg12341018	6	56176155			OpenSea	0.284	0.231	0.053	2.04E-04	1.88E-03
cg22798247	6	32807372	TAP2	TSS1500	S_Shore	0.862	0.736	0.126	2.05E-04	1.88E-03
cg26861460	22	44575455	PARVG	TSS1500;5'UTR;Body	OpenSea	0.528	0.474	0.054	2.06E-04	1.89E-03
cg18854872	2	198292151	SF3B1	Body	OpenSea	0.631	0.576	0.056	2.06E-04	1.89E-03
cg01966791	20	62572875	MIR1914;UCKL1	Body	S_Shore	0.479	0.411	0.068	2.08E-04	1.91E-03
cg21549285	21	42799141	MX1	5'UTR	S_Shore	0.552	0.614	-0.062	2.12E-04	1.93E-03
cg07023317	8	28961315	KIF13B	Body	OpenSea	0.740	0.688	0.053	2.13E-04	1.93E-03
cg03673989	11	380069	B4GALNT4	Body	Island	0.884	0.828	0.055	2.14E-04	1.94E-03
cg12884009	2	219079038			N_Shelf	0.780	0.729	0.051	2.21E-04	1.99E-03
cg16462183	5	54281733	ESM1;ESM1	TSS1500	OpenSea	0.606	0.552	0.053	2.22E-04	1.99E-03
cg24142603	8	72753888	MSC	3'UTR	Island	0.789	0.851	-0.062	2.28E-04	2.03E-03
cg14312063	3	85010606	CADM2	Body	S_Shore	0.505	0.443	0.062	2.30E-04	2.05E-03
cg03062454	21	30362034	RNF160	Body	N_Shelf	0.573	0.522	0.051	2.33E-04	2.07E-03
cg22422264	15	50792856	USP50;USP8	3'UTR	OpenSea	0.728	0.678	0.050	2.35E-04	2.08E-03
cg27223827	2	106054920	FHL2	1stExon;5'UTR	OpenSea	0.806	0.752	0.054	2.36E-04	2.08E-03
cg11058932	7	130372167	TSGA13	TSS1500	OpenSea	0.706	0.649	0.057	2.39E-04	2.10E-03
cg04777091	4	6050440	JAKMIP1	Body	OpenSea	0.229	0.172	0.058	2.41E-04	2.11E-03
cg16858211	7	36765349	AOAH	TSS1500	OpenSea	0.660	0.607	0.053	2.46E-04	2.14E-03
cg15694715	11	118402280	TMEM25	5'UTR	S_Shore	0.331	0.278	0.052	2.46E-04	2.15E-03
cg13059136	11	2986541	SNORA54;NAP1L4	TSS1500;Body	OpenSea	0.664	0.607	0.056	2.47E-04	2.15E-03
cg21078654	15	52130305	TMOD3	5'UTR	OpenSea	0.287	0.233	0.054	2.48E-04	2.16E-03
cg25711003	3	111522050	PHLDB2;PLCXD2	5'UTR;Body	OpenSea	0.755	0.704	0.050	2.52E-04	2.18E-03
cg05351887	16	3988869			N_Shore	0.425	0.362	0.063	2.55E-04	2.20E-03
cg02566775	6	144382964	PLAGL1	5'UTR	N_Shore	0.570	0.518	0.052	2.57E-04	2.21E-03
cg12961733	22	50165244			Island	0.552	0.498	0.054	2.58E-04	2.21E-03
cg16962463	12	89968675			OpenSea	0.281	0.226	0.055	2.61E-04	2.23E-03
cg12285640	6	140001095			OpenSea	0.738	0.681	0.057	2.61E-04	2.24E-03
cg21390082	17	33842255	SLFN12L	Body	OpenSea	0.852	0.795	0.057	2.63E-04	2.25E-03
cg09723776	8	126320143	NSMCE2	Body	OpenSea	0.626	0.573	0.053	2.66E-04	2.26E-03
cg16395997	1	3562798	WDR8	Body	S_Shore	0.774	0.718	0.057	2.67E-04	2.27E-03
cg22716738	7	27784932	TAX1BP1	5'UTR	OpenSea	0.424	0.359	0.064	2.67E-04	2.27E-03

cg10409299	7	150498843	TMEM176B;TMEM176A	TSS1500;Body	S_Shore	0.717	0.665	0.052	2.71E-04	2.29E-03
cg25338454	12	26900022	ITPR2	Body	OpenSea	0.764	0.706	0.058	2.76E-04	2.32E-03
cg21992696	2	9125026	MBOAT2	Body	OpenSea	0.429	0.362	0.066	2.78E-04	2.33E-03
cg06122613	1	151297900	PI4KB	Body	N_Shelf	0.757	0.700	0.057	2.80E-04	2.34E-03
cg23705979	7	92397161	CDK6	Body	OpenSea	0.354	0.301	0.053	2.83E-04	2.36E-03
cg02710015	12	55362424	KIAA0748	Body	OpenSea	0.498	0.430	0.069	2.84E-04	2.37E-03
cg05697976	12	29376483	FAR2	TSS200	OpenSea	0.518	0.453	0.066	2.86E-04	2.38E-03
cg13088432	8	9522664	TNKS	Body	OpenSea	0.570	0.498	0.072	2.87E-04	2.39E-03
cg11168614	14	87179368			OpenSea	0.651	0.588	0.063	2.87E-04	2.39E-03
cg02264407	13	76430665	LMO7	Body	OpenSea	0.833	0.777	0.056	2.90E-04	2.40E-03
cg14111928	10	76602391	MYST4	5'UTR	OpenSea	0.711	0.655	0.056	2.93E-04	2.42E-03
cg04231677	1	184808004	FAM129A	Body	OpenSea	0.519	0.453	0.066	2.95E-04	2.43E-03
cg09982419	15	68343575			N_Shelf	0.641	0.582	0.059	3.00E-04	2.46E-03
cg05888181	8	1952319	KBTBD11	3'UTR	S_Shore	0.666	0.598	0.068	3.06E-04	2.50E-03
cg13424302	10	80516893			OpenSea	0.417	0.359	0.058	3.06E-04	2.50E-03
cg08195176	11	116781303	SIK3	Body	OpenSea	0.604	0.551	0.053	3.07E-04	2.50E-03
cg08836481	10	882938	LARP4B	Body	OpenSea	0.760	0.708	0.052	3.08E-04	2.51E-03
cg08110488	1	82136224			OpenSea	0.282	0.228	0.053	3.08E-04	2.51E-03
cg04673465	4	85570164	CDS1	3'UTR	OpenSea	0.533	0.480	0.053	3.10E-04	2.52E-03
cg11163555	22	31736976	PATZ1	Body;3'UTR	OpenSea	0.397	0.346	0.051	3.20E-04	2.57E-03
cg10299128	4	82796846			OpenSea	0.485	0.430	0.054	3.21E-04	2.58E-03
cg03633574	3	149954846			OpenSea	0.804	0.752	0.052	3.22E-04	2.59E-03
cg20482280	14	105936300	MTA1	Body	Island	0.667	0.615	0.052	3.24E-04	2.60E-03
cg00416710	16	29196027			N_Shore	0.694	0.635	0.059	3.24E-04	2.60E-03
cg18497052	5	107148418			OpenSea	0.393	0.342	0.051	3.25E-04	2.60E-03
cg16678522	20	32149963	CBFA2T2	Body;TSS1500	OpenSea	0.698	0.644	0.054	3.27E-04	2.61E-03
cg19494591	7	140174476	MKRN1	Body	N_Shelf	0.794	0.744	0.051	3.32E-04	2.64E-03
cg05454562	6	33254447	WDR46	Body	N_Shelf	0.624	0.566	0.058	3.41E-04	2.69E-03
cg21301224	8	56813677	LYN	5'UTR	OpenSea	0.365	0.312	0.053	3.42E-04	2.69E-03
cg20610950	17	75096202			OpenSea	0.765	0.700	0.065	3.43E-04	2.70E-03
cg14251964	8	18396499	PSD3	Body	OpenSea	0.949	0.892	0.057	3.44E-04	2.70E-03
cg08598221	8	121824929	SNTB1	TSS1500	S_Shore	0.589	0.534	0.055	3.49E-04	2.73E-03
cg11952340	15	63814440	USP3	Body	OpenSea	0.604	0.547	0.056	3.49E-04	2.73E-03
cg07104135	1	201799785	IPO9	Body	S_Shore	0.273	0.220	0.053	3.51E-04	2.74E-03
cg10499974	3	46244099	CCR1	3'UTR	OpenSea	0.668	0.614	0.054	3.52E-04	2.75E-03
cg26105956	1	154471433	SHE	Body	N_Shelf	0.733	0.668	0.065	3.52E-04	2.75E-03
cg10536276	2	113956344	PSD4;LOC440839	Body	Island	0.594	0.543	0.050	3.54E-04	2.76E-03
cg19072817	5	176057992	EIF4E1B;SNCB	5'UTR;TSS1500;1stExon	S_Shore	0.325	0.269	0.055	3.57E-04	2.77E-03
cg06645778	2	64680425	HSPC159	TSS1500	N_Shore	0.565	0.513	0.052	3.62E-04	2.80E-03
cg27073113	16	56228744	DKFZP434H168;GNAO1	TSS1500;Body;Body	S_Shelf	0.289	0.224	0.065	3.64E-04	2.81E-03
cg02320474	16	1758022	MAPK8IP3	Body	OpenSea	0.434	0.371	0.063	3.65E-04	2.82E-03
cg11900393	12	721502	NINJ2	Body	S_Shelf	0.737	0.685	0.052	3.69E-04	2.84E-03
cg17775490	20	45179354	C20orf123	TSS200	OpenSea	0.269	0.216	0.053	3.71E-04	2.85E-03
cg05492071	2	202650064			S_Shelf	0.601	0.540	0.061	3.77E-04	2.88E-03
cg23232188	3	121556543	EAF2	Body	S_Shelf	0.789	0.734	0.055	3.82E-04	2.91E-03
cg26253500	7	136641740	CHRM2	5'UTR	OpenSea	0.758	0.703	0.055	3.84E-04	2.92E-03
cg21216258	9	140942584	CACNA1B	Body	S_Shore	0.590	0.518	0.073	3.87E-04	2.93E-03
cg19869035	7	2653955	IQCE	3'UTR	OpenSea	0.531	0.481	0.050	3.88E-04	2.94E-03

cg10401017	6	28887586	TRIM27	Body	N_Shelf	0.830	0.767	0.063	3.89E-04	2.94E-03
cg26742320	2	201393628	SGOL2	5'UTR	S_Shelf	0.806	0.755	0.051	3.98E-04	2.99E-03
cg07805500	6	151380818	MTHFD1L	Body	OpenSea	0.480	0.391	0.089	4.05E-04	3.02E-03
cg09439920	3	99979117	TBC1D23	TSS1500	N_Shore	0.447	0.395	0.052	4.13E-04	3.06E-03
cg26828017	16	22409023			OpenSea	0.684	0.623	0.060	4.15E-04	3.07E-03
cg07229212	8	11056726	XKR6	Body	N_Shore	0.605	0.546	0.059	4.18E-04	3.09E-03
cg01235820	20	642782	SCRT2	3'UTR	Island	0.669	0.618	0.050	4.19E-04	3.09E-03
cg07113414	8	144652930	C8orf73	Body	N_Shore	0.551	0.485	0.065	4.19E-04	3.10E-03
cg12237948	20	2085157	STK35	Body	S_Shore	0.724	0.662	0.062	4.20E-04	3.10E-03
cg20732703	7	50744032	GRB10	5'UTR;Body	OpenSea	0.521	0.470	0.051	4.21E-04	3.11E-03
cg11261850	11	76320970			OpenSea	0.382	0.329	0.053	4.22E-04	3.11E-03
cg22159528	19	44039727	ZNF575	Body	Island	0.605	0.551	0.055	4.23E-04	3.12E-03
cg05279761	3	132312243	ACAD11	Body	OpenSea	0.568	0.498	0.069	4.28E-04	3.14E-03
cg23207876	19	15162821	CASP14	TSS200	OpenSea	0.910	0.850	0.060	4.29E-04	3.15E-03
cg09940188	17	1686671	SMYD4	Body	OpenSea	0.599	0.538	0.061	4.37E-04	3.19E-03
cg07922513	1	6089454	KCNAB2	5'UTR	S_Shelf	0.748	0.696	0.052	4.40E-04	3.20E-03
cg21426759	6	30303126	TRIM39	Body	OpenSea	0.656	0.603	0.053	4.42E-04	3.21E-03
cg13640297	6	170099238	WDR27	5'UTR	N_Shelf	0.676	0.625	0.051	4.42E-04	3.22E-03
cg05894754	2	210675440	UNC80;UNC80	Body	S_Shore	0.801	0.743	0.058	4.43E-04	3.22E-03
cg07541020	10	44068714	ZNF239	5'UTR	N_Shore	0.703	0.640	0.063	4.46E-04	3.23E-03
cg26319363	4	753903	PCGF3	Body	S_Shore	0.922	0.864	0.058	4.46E-04	3.23E-03
cg19140262	6	99380488	FBXL4	5'UTR	OpenSea	0.245	0.195	0.050	4.49E-04	3.25E-03
cg01924292	12	1815334	ADIPOR2	5'UTR	OpenSea	0.656	0.584	0.072	4.52E-04	3.26E-03
cg05431947	11	12621248			OpenSea	0.275	0.224	0.050	4.55E-04	3.28E-03
cg16809457	6	90399677	MDN1	Body	OpenSea	0.694	0.644	0.050	4.56E-04	3.28E-03
cg01943931	14	73373205			OpenSea	0.886	0.829	0.057	4.70E-04	3.35E-03
cg07405121	16	2083168	SLC9A3R2	Body	N_Shelf	0.472	0.422	0.050	4.73E-04	3.37E-03
cg00117012	15	73996358	CD276	Body	OpenSea	0.810	0.708	0.102	4.74E-04	3.37E-03
cg15586392	8	142238691	SLC45A4	TSS200	S_Shore	0.612	0.554	0.058	4.81E-04	3.41E-03
cg19851560	2	88390167	SMYD1	Body	OpenSea	0.696	0.644	0.052	4.98E-04	3.49E-03
cg22630754	15	42866523	STARD9	TSS1500	N_Shore	0.687	0.626	0.061	5.04E-04	3.52E-03
cg07948875	1	169187004	NME7	Body	OpenSea	0.314	0.257	0.058	5.15E-04	3.57E-03
cg08805500	12	100522155	UHRF1BP1L	Body	OpenSea	0.306	0.250	0.056	5.17E-04	3.58E-03
cg08760493	4	109994039	COL25A1	Body	OpenSea	0.533	0.480	0.053	5.18E-04	3.59E-03
cg02819655	2	178853377	PDE11A	Body	OpenSea	0.911	0.857	0.054	5.19E-04	3.59E-03
cg22612003	2	235849504			OpenSea	0.900	0.839	0.061	5.26E-04	3.63E-03
cg13185177	3	194119885	GP5	5'UTR	S_Shore	0.739	0.676	0.062	5.27E-04	3.63E-03
cg24753094	15	71890066	THSD4	Body	OpenSea	0.706	0.579	0.128	5.29E-04	3.64E-03
cg11321181	19	17721489	UNC13A	Body	S_Shelf	0.374	0.310	0.063	5.35E-04	3.67E-03
cg12492273	7	2119499	MAD1L1	Body	S_Shelf	0.433	0.374	0.059	5.41E-04	3.69E-03
cg10603275	3	239665	CHL1	5'UTR	Island	0.180	0.125	0.055	5.49E-04	3.73E-03
cg18507018	8	92035517	TMEM55A	Body	OpenSea	0.726	0.654	0.072	5.52E-04	3.75E-03
cg21967368	2	211422328	CPS1;CPS1	Body	OpenSea	0.487	0.429	0.058	5.52E-04	3.75E-03
cg01431340	6	110680085	C6orf186	TSS1500	S_Shore	0.458	0.391	0.067	5.55E-04	3.76E-03
cg25734726	19	40948296	SERTAD3	1stExon;5'UTR	N_Shore	0.611	0.555	0.056	5.56E-04	3.77E-03
cg24406240	11	88153520			OpenSea	0.614	0.549	0.065	5.57E-04	3.77E-03
cg25698842	8	1110263			N_Shelf	0.754	0.675	0.079	5.65E-04	3.81E-03
cg14308082	10	131568021			OpenSea	0.596	0.542	0.054	5.68E-04	3.82E-03

cg15219811	10	104259328	ACTR1A	Body	N_Shelf	0.760	0.709	0.051	5.68E-04	3.83E-03
cg26445561	11	92925402	SLC36A4	Body	OpenSea	0.760	0.709	0.050	5.70E-04	3.83E-03
cg03639671	4	145430689			OpenSea	0.645	0.593	0.052	5.70E-04	3.83E-03
cg26975524	8	1713012	CLN8	5'UTR	S_Shore	0.647	0.587	0.060	5.74E-04	3.85E-03
cg16689761	7	141360223	KIAA1147	3'UTR	OpenSea	0.298	0.246	0.051	5.75E-04	3.86E-03
cg06162038	2	138727217	HNMT	Body	OpenSea	0.370	0.315	0.055	5.78E-04	3.87E-03
cg08941853	1	59449033			OpenSea	0.445	0.381	0.064	5.79E-04	3.88E-03
cg09069499	8	26216910	PPP2R2A	Body	OpenSea	0.770	0.713	0.057	5.80E-04	3.88E-03
cg19116959	4	146841472	ZNF827	Body	OpenSea	0.479	0.421	0.058	5.81E-04	3.89E-03
cg21597811	8	123687197			OpenSea	0.685	0.631	0.054	5.84E-04	3.89E-03
cg12437013	13	114161939	TMCO3	Body	N_Shelf	0.647	0.556	0.091	5.84E-04	3.90E-03
cg26162326	7	75957061	YWHAG	3'UTR	N_Shore	0.507	0.442	0.064	5.85E-04	3.90E-03
cg03864128	20	32398023	CHMP4B	TSS1500	N_Shore	0.783	0.729	0.054	5.88E-04	3.91E-03
cg26872780	2	149478494	EPC2	Body	OpenSea	0.631	0.571	0.059	5.90E-04	3.92E-03
cg00777445	17	48970357			OpenSea	0.652	0.592	0.061	5.96E-04	3.95E-03
cg24575128	3	52502445	NISCH	Body	OpenSea	0.513	0.458	0.056	6.00E-04	3.96E-03
cg02282631	5	42953543			S_Shore	0.696	0.635	0.060	6.03E-04	3.98E-03
cg03967798	4	145268453			OpenSea	0.535	0.481	0.054	6.09E-04	4.01E-03
cg04998202	1	61545546	NFIA	Body	N_Shelf	0.399	0.333	0.066	6.19E-04	4.05E-03
cg19238325	3	51988425	GPR62	TSS1500	N_Shore	0.386	0.335	0.051	6.29E-04	4.10E-03
cg21783012	1	2710376			S_Shelf	0.913	0.813	0.100	6.36E-04	4.13E-03
cg16520357	13	53625240	OLFM4	3'UTR	OpenSea	0.726	0.667	0.059	6.37E-04	4.13E-03
cg27332878	15	80765904	ARNT2	Body	OpenSea	0.261	0.203	0.058	6.38E-04	4.14E-03
cg05624376	2	169939876	DHRS9	Body	OpenSea	0.568	0.513	0.055	6.42E-04	4.16E-03
cg04350202	10	111653363	XPNPEP1	Body	OpenSea	0.554	0.501	0.053	6.43E-04	4.16E-03
cg25116430	5	151908977			OpenSea	0.801	0.745	0.055	6.46E-04	4.18E-03
cg07377422	19	46318633	RSPH6A	TSS200	Island	0.222	0.168	0.054	6.62E-04	4.24E-03
cg25865108	6	45559104			OpenSea	0.668	0.606	0.062	6.71E-04	4.28E-03
cg05265607	10	98900817	SLIT1	Body	OpenSea	0.695	0.628	0.067	6.74E-04	4.30E-03
cg00421173	4	140603682	MGST2	Body	OpenSea	0.855	0.796	0.059	6.74E-04	4.30E-03
cg03269667	3	9744891	CPNE9	TSS1500	N_Shore	0.920	0.819	0.101	6.75E-04	4.30E-03
cg23969338	5	140474005	PCDHB2	TSS1500	N_Shore	0.462	0.408	0.055	6.84E-04	4.34E-03
cg09643312	2	160655081	CD302	TSS1500	S_Shore	0.492	0.434	0.057	6.86E-04	4.35E-03
cg20048037	8	120605539	ENPP2	Body	OpenSea	0.581	0.528	0.054	6.88E-04	4.36E-03
cg21222426	9	20339790			OpenSea	0.340	0.281	0.059	6.91E-04	4.37E-03
cg09101062	5	43487508	C5orf34	Body	S_Shelf	0.857	0.794	0.062	6.96E-04	4.40E-03
cg11578532	1	161008127	TSTD1	Body	N_Shore	0.513	0.459	0.054	7.00E-04	4.42E-03
cg13062455	14	81978021	SEL1L	Body	OpenSea	0.615	0.546	0.069	7.01E-04	4.42E-03
cg26620655	7	1113029	C7orf50	Body	OpenSea	0.494	0.432	0.062	7.19E-04	4.50E-03
cg15965190	2	208370032			OpenSea	0.297	0.242	0.055	7.22E-04	4.51E-03
cg19173375	6	27880117	OR2B2	TSS200	OpenSea	0.403	0.344	0.060	7.23E-04	4.51E-03
cg05696877	1	79088769	IFI44L	5'UTR	OpenSea	0.449	0.521	-0.072	7.33E-04	4.56E-03
cg24403804	6	16805426			OpenSea	0.284	0.231	0.053	7.33E-04	4.56E-03
cg03950493	11	126582183	KIRREL3	Body	OpenSea	0.516	0.443	0.073	7.49E-04	4.62E-03
cg23395177	14	77498304			N_Shore	0.693	0.585	0.109	7.51E-04	4.63E-03
cg23403750	5	114610319	CCDC112	Body	OpenSea	0.697	0.643	0.054	7.62E-04	4.68E-03
cg03818715	6	7591348	SNRNP48	Body	S_Shore	0.588	0.528	0.059	7.62E-04	4.68E-03
cg17083429	10	89627854	PTEN	Body	S_Shelf	0.507	0.436	0.071	7.65E-04	4.69E-03

cg04885885	16	54225800			N_Shelf	0.606	0.463	0.143	7.65E-04	4.69E-03
cg21341487	20	60760919	GTPBP5	5'UTR	S_Shelf	0.497	0.440	0.057	7.71E-04	4.71E-03
cg26537280	1	95699037	RWDD3	TSS1500	N_Shore	0.479	0.418	0.061	7.72E-04	4.72E-03
cg21444421	12	90393807			OpenSea	0.585	0.525	0.060	7.75E-04	4.73E-03
cg09858862	1	23887514	ID3	TSS1500	S_Shore	0.585	0.518	0.067	7.80E-04	4.75E-03
cg04936009	2	223846845			OpenSea	0.582	0.529	0.053	7.82E-04	4.76E-03
cg10880928	9	139715701	C9orf86	Body	Island	0.370	0.318	0.052	7.84E-04	4.77E-03
cg25656283	10	50733196	ERCC6;PGBD3	Body;TSS1500	OpenSea	0.762	0.709	0.053	7.99E-04	4.83E-03
cg21005683	4	159191054			OpenSea	0.948	0.895	0.053	8.02E-04	4.84E-03
cg04139465	19	51020297	LRRC4B	3'UTR	Island	0.540	0.468	0.071	8.06E-04	4.86E-03
cg24599598	10	3050893			OpenSea	0.433	0.382	0.051	8.12E-04	4.89E-03
cg01701649	16	48589954	N4BP1	Body	OpenSea	0.382	0.316	0.067	8.27E-04	4.95E-03
cg15084543	1	79472408	ELTD1	5'UTR;1stExon	Island	0.360	0.298	0.061	8.44E-04	5.02E-03
cg24361586	17	62744534			OpenSea	0.709	0.653	0.056	8.53E-04	5.06E-03
cg20384240	1	183799423	RGL1	Body	OpenSea	0.368	0.313	0.055	8.54E-04	5.06E-03
cg26298737	1	225964937	SRP9;SRP9	TSS1500	N_Shore	0.572	0.519	0.053	8.56E-04	5.07E-03
cg11942594	3	167659189			OpenSea	0.223	0.173	0.050	8.56E-04	5.07E-03
cg22243039	17	42147465	G6PC3	TSS1500	N_Shore	0.337	0.282	0.055	8.66E-04	5.11E-03
cg27260684	16	85063742	KIAA0513	5'UTR	S_Shore	0.502	0.444	0.058	8.71E-04	5.13E-03
cg12628061	1	56453730			OpenSea	0.767	0.701	0.066	8.73E-04	5.13E-03
cg00364747	8	10915402	XKR6	Body	N_Shore	0.968	0.912	0.056	8.81E-04	5.17E-03
cg09506675	7	112727914	GPR85	TSS1500;TSS200;TSS200	OpenSea	0.546	0.491	0.055	8.81E-04	5.17E-03
cg03075631	2	74697095			N_Shelf	0.367	0.526	-0.160	8.91E-04	5.21E-03
cg14191024	11	110070509			OpenSea	0.727	0.665	0.061	8.94E-04	5.22E-03
cg24553417	13	50244794	EBPL	Body	OpenSea	0.570	0.519	0.051	8.98E-04	5.24E-03
cg01121603	2	242005101	SNED1	Body	Island	0.804	0.753	0.051	8.99E-04	5.24E-03
cg08023751	2	112655112	MERTK	TSS1500	N_Shore	0.689	0.638	0.052	9.01E-04	5.25E-03
cg27584135	3	15975501			OpenSea	0.609	0.536	0.073	9.04E-04	5.26E-03
cg09357350	3	171894094	FNDC3B	Body	OpenSea	0.641	0.583	0.058	9.07E-04	5.27E-03
cg06881914	11	59951663	MS4A6A	TSS1500	OpenSea	0.642	0.591	0.051	9.12E-04	5.29E-03
cg23670353	1	59833489	FGGY	Body	OpenSea	0.540	0.479	0.061	9.17E-04	5.31E-03
cg17359629	13	114259310	TFDP1	Body	N_Shore	0.651	0.592	0.059	9.19E-04	5.32E-03
cg02884928	14	105251610	AKT1	Body	Island	0.600	0.543	0.058	9.20E-04	5.33E-03
cg18084609	14	31345041	COCH	Body	S_Shore	0.603	0.551	0.052	9.21E-04	5.33E-03
cg25577212	7	24740509	DFNA5	Body	OpenSea	0.896	0.839	0.057	9.25E-04	5.35E-03
cg11588787	13	28181449	LNX2	5'UTR	OpenSea	0.345	0.279	0.065	9.28E-04	5.36E-03
cg26642420	4	146601345	C4orf51	TSS200	OpenSea	0.742	0.690	0.052	9.32E-04	5.38E-03
cg11345976	2	30864107	LCLAT1	3'UTR	OpenSea	0.759	0.685	0.073	9.37E-04	5.39E-03
cg27588384	3	45674281	LIMD1	Body	OpenSea	0.740	0.684	0.056	9.43E-04	5.42E-03
cg09729012	3	72395774			OpenSea	0.396	0.325	0.071	9.46E-04	5.43E-03
cg17797940	3	171759519	FNDC3B	5'UTR	S_Shore	0.539	0.479	0.061	9.52E-04	5.46E-03
cg20255272	1	1373678	VWA1	Body;3'UTR	N_Shore	0.570	0.519	0.051	9.60E-04	5.49E-03
cg04476070	16	88679600	ZC3H18	Body	OpenSea	0.737	0.680	0.057	9.61E-04	5.49E-03
cg19782686	4	124180616	SPATA5	Body	OpenSea	0.540	0.477	0.063	9.64E-04	5.51E-03
cg19214707	7	3157722			OpenSea	0.398	0.238	0.160	9.66E-04	5.51E-03
cg15843217	4	699224	PCGF3	TSS1500	N_Shore	0.442	0.390	0.052	9.68E-04	5.52E-03
cg13607226	9	124262699	GGTA1	TSS1500	S_Shore	0.519	0.460	0.058	9.72E-04	5.54E-03
cg26536240	16	89509760	ANKRD11	5'UTR	OpenSea	0.742	0.683	0.058	9.75E-04	5.55E-03

cg00357551	5	169407472	FAM196B;DOCK2	5'UTR;Body;1stExon	OpenSea	0.540	0.485	0.056	9.77E-04	5.55E-03
cg26282731	4	148939288	ARHGAP10	Body	OpenSea	0.761	0.710	0.051	9.78E-04	5.56E-03
cg13276631	3	46872239	PRSS42	Body	N_Shelf	0.845	0.774	0.071	9.88E-04	5.60E-03
cg24407596	7	98718683	SMURF1	Body	OpenSea	0.576	0.510	0.066	9.89E-04	5.60E-03
cg10722267	3	197608554			OpenSea	0.779	0.718	0.061	9.97E-04	5.64E-03
cg09039751	11	45672100	CHST1	Body	Island	0.719	0.666	0.053	1.00E-03	5.65E-03
cg01176720	10	35081047	PARD3	Body	OpenSea	0.917	0.857	0.060	1.01E-03	5.68E-03
cg08178956	2	42345201			OpenSea	0.403	0.336	0.067	1.01E-03	5.71E-03
cg26132114	10	44223934			Island	0.714	0.659	0.055	1.02E-03	5.71E-03
cg05382012	16	67517841	AGRP	TSS200;TSS1500	S_Shelf	0.542	0.491	0.051	1.02E-03	5.73E-03
cg04592413	2	8379999			OpenSea	0.239	0.187	0.053	1.03E-03	5.75E-03
cg14177325	17	1541763	SCARF1	Body	S_Shelf	0.669	0.618	0.051	1.03E-03	5.75E-03
cg14207267	12	113655841	IQCD	5'UTR	N_Shelf	0.398	0.337	0.061	1.04E-03	5.78E-03
cg23254569	6	35451487	TEAD3	Body	N_Shelf	0.531	0.479	0.052	1.05E-03	5.85E-03
cg14003416	2	12557155			OpenSea	0.604	0.551	0.053	1.06E-03	5.86E-03
cg22283115	7	4911121	RADIL	Body	OpenSea	0.576	0.523	0.053	1.07E-03	5.92E-03
cg18234111	1	158155757	CD1D	3'UTR	OpenSea	0.298	0.247	0.051	1.08E-03	5.95E-03
cg06193043	1	11908199	NPPA	TSS1500	OpenSea	0.524	0.472	0.052	1.08E-03	5.96E-03
cg27073431	2	47404636	CALM2	TSS1500	S_Shore	0.680	0.629	0.051	1.09E-03	5.99E-03
cg06347643	2	137518979			N_Shelf	0.928	0.855	0.073	1.09E-03	6.00E-03
cg02852959	12	2944436	NRIP2	TSS1500	OpenSea	0.604	0.539	0.065	1.11E-03	6.08E-03
cg08742502	6	106546754	PRDM1	Body;TSS200	OpenSea	0.405	0.461	-0.056	1.11E-03	6.09E-03
cg23605961	7	751331	PRKAR1B	5'UTR	N_Shore	0.553	0.493	0.060	1.12E-03	6.10E-03
cg22566142	5	96298493	LNPEP	Body;5'UTR	OpenSea	0.438	0.384	0.054	1.12E-03	6.13E-03
cg07319315	2	135149599	MGAT5	Body	OpenSea	0.706	0.642	0.064	1.12E-03	6.13E-03
cg08122070	1	1689610	NADK	Body	N_Shelf	0.733	0.678	0.055	1.13E-03	6.13E-03
cg11002686	7	55412682			Island	0.523	0.472	0.051	1.13E-03	6.16E-03
cg20168849	3	188995030	TPRG1	Body	OpenSea	0.591	0.527	0.064	1.13E-03	6.17E-03
cg23985818	4	94607780	GRID2	Body	OpenSea	0.832	0.780	0.052	1.13E-03	6.17E-03
cg04784315	9	125797284	GPR21;RABGAP1	1stExon;Body	OpenSea	0.546	0.492	0.054	1.14E-03	6.18E-03
cg00261690	1	28856281	SNHG3-RCC1;RCC1	Body;5'UTR	N_Shore	0.613	0.553	0.060	1.14E-03	6.19E-03
cg18517369	2	231899207			N_Shelf	0.596	0.543	0.053	1.15E-03	6.22E-03
cg26426488	7	100461644	SLC12A9	Body	N_Shelf	0.452	0.393	0.059	1.16E-03	6.26E-03
cg24856673	12	2017134	CACNA2D4	Body	Island	0.343	0.283	0.060	1.17E-03	6.29E-03
cg14293575	22	18635460	USP18	5'UTR	S_Shelf	0.617	0.688	-0.071	1.17E-03	6.31E-03
cg02262553	4	87849250			OpenSea	0.506	0.453	0.052	1.18E-03	6.33E-03
cg15704662	8	128388831			OpenSea	0.696	0.639	0.057	1.18E-03	6.34E-03
cg15073631	13	104796427			OpenSea	0.379	0.329	0.051	1.19E-03	6.36E-03
cg14942952	22	44576268	PARVG	TSS1500;5'UTR;Body	OpenSea	0.588	0.523	0.066	1.19E-03	6.38E-03
cg05131696	12	31499320			OpenSea	0.441	0.373	0.068	1.19E-03	6.38E-03
cg00754253	11	63259187	HRASLS5	TSS1500	S_Shore	0.447	0.392	0.055	1.22E-03	6.48E-03
cg02922776	1	212420632			OpenSea	0.467	0.402	0.065	1.22E-03	6.49E-03
cg14646039	5	152729842			OpenSea	0.602	0.545	0.056	1.23E-03	6.51E-03
cg19049109	7	27450882			OpenSea	0.877	0.762	0.115	1.24E-03	6.56E-03
cg17837191	17	62318673	TEX2	5'UTR	OpenSea	0.555	0.502	0.053	1.24E-03	6.56E-03
cg10230190	1	85405081	MCOLN2	Body	OpenSea	0.747	0.695	0.053	1.27E-03	6.67E-03
cg04860432	14	52783454	PTGER2	Body	S_Shore	0.528	0.473	0.055	1.27E-03	6.68E-03
cg22629528	11	132951861	OPCML	Body	Island	0.638	0.578	0.060	1.29E-03	6.72E-03

cg01305421	12	102874286	IGF1	5'UTR;1stExon	OpenSea	0.326	0.267	0.059	1.31E-03	6.79E-03
cg04089901	7	7557590	COL28A1	Body	OpenSea	0.536	0.483	0.053	1.31E-03	6.80E-03
cg21476666	3	157221200	VEPH1	TSS200;5'UTR	OpenSea	0.832	0.780	0.052	1.32E-03	6.83E-03
cg08730245	12	53902893	NPFF	TSS1500	OpenSea	0.645	0.590	0.056	1.32E-03	6.84E-03
cg01664300	6	15911690			OpenSea	0.703	0.589	0.114	1.32E-03	6.84E-03
cg00453717	11	74344629	POLD3	Body	OpenSea	0.572	0.516	0.056	1.32E-03	6.85E-03
cg10718056	6	28884599	TRIM27	Body	OpenSea	0.515	0.462	0.053	1.38E-03	7.05E-03
cg19356022	1	154943932	SHC1	5'UTR;5'UTR;TSS1500;TSS1500	N_Shelf	0.558	0.506	0.051	1.39E-03	7.07E-03
cg00325531	1	75591353			Island	0.219	0.167	0.051	1.39E-03	7.10E-03
cg00782708	2	44933278	C2orf34	Body	OpenSea	0.635	0.581	0.053	1.40E-03	7.14E-03
cg07759162	15	75488313			OpenSea	0.615	0.561	0.054	1.43E-03	7.23E-03
cg16151538	20	33676388	TRPC4AP	Body	N_Shelf	0.722	0.669	0.053	1.43E-03	7.24E-03
cg14318679	17	700464			OpenSea	0.880	0.828	0.052	1.45E-03	7.28E-03
cg17696517	8	105343133			S_Shore	0.325	0.269	0.056	1.45E-03	7.29E-03
cg26629715	8	89379430			OpenSea	0.788	0.719	0.069	1.45E-03	7.31E-03
cg06106428	11	110584327	ARHGAP20	TSS1500	S_Shore	0.740	0.686	0.054	1.46E-03	7.35E-03
cg19989043	6	47468193	CD2AP	Body	OpenSea	0.417	0.353	0.064	1.48E-03	7.40E-03
cg11147155	1	150595206	ENSA	3'UTR	OpenSea	0.493	0.442	0.051	1.48E-03	7.41E-03
cg05758467	2	240035894	HDAC4	Body	S_Shelf	0.572	0.513	0.059	1.48E-03	7.42E-03
cg05980785	2	197583891	CCDC150	Body	OpenSea	0.527	0.460	0.067	1.49E-03	7.43E-03
cg23668184	2	5836713	SOX11	3'UTR;1stExon	Island	0.144	0.093	0.050	1.49E-03	7.44E-03
cg05788125	6	170559721			OpenSea	0.609	0.555	0.054	1.49E-03	7.44E-03
cg06579481	7	104621597			N_Shelf	0.675	0.623	0.052	1.50E-03	7.46E-03
cg17591816	1	172239092	DNM3	Body	OpenSea	0.728	0.667	0.060	1.51E-03	7.50E-03
cg22022881	2	105853494			Island	0.569	0.495	0.074	1.51E-03	7.52E-03
cg18188328	6	15505094	JARID2	Body	OpenSea	0.735	0.680	0.055	1.51E-03	7.52E-03
cg20155875	17	66452567	WIPI1	Body	N_Shore	0.620	0.563	0.057	1.52E-03	7.54E-03
cg06548519	17	34267111	LYZL6	TSS1500	OpenSea	0.682	0.630	0.052	1.53E-03	7.59E-03
cg02462081	2	220405635	CHPF	Body	N_Shore	0.815	0.763	0.053	1.57E-03	7.70E-03
cg17036458	17	28037399	SSH2	Body	OpenSea	0.633	0.565	0.068	1.58E-03	7.75E-03
cg10231675	12	2944493	NRIP2	TSS1500	OpenSea	0.596	0.537	0.059	1.60E-03	7.83E-03
cg08113187	16	87469329	ZCCHC14	Body	OpenSea	0.251	0.196	0.055	1.61E-03	7.83E-03
cg23201812	11	34771918			OpenSea	0.624	0.564	0.060	1.63E-03	7.92E-03
cg11740416	3	194409431	FAM43A	3'UTR;1stExon	S_Shore	0.321	0.259	0.062	1.64E-03	7.95E-03
cg20992708	4	5637894	EVC2	Body	OpenSea	0.529	0.472	0.057	1.64E-03	7.95E-03
cg19322969	1	226854847	ITPKB	Body	OpenSea	0.790	0.737	0.053	1.65E-03	7.96E-03
cg17657707	20	47541362	ARFGEF2	Body	S_Shelf	0.478	0.425	0.053	1.65E-03	7.97E-03
cg25769566	6	31651278			Island	0.750	0.691	0.060	1.65E-03	7.98E-03
cg14543285	14	103129037	RCOR1	Body	OpenSea	0.602	0.552	0.050	1.66E-03	8.01E-03
cg12475879	17	14251824			S_Shelf	0.754	0.696	0.058	1.67E-03	8.05E-03
cg13480937	1	177112230	ASTN1	Body	OpenSea	0.500	0.433	0.066	1.68E-03	8.07E-03
cg05667818	7	116786870	ST7;ST7OT2	Body;TSS1500	OpenSea	0.473	0.412	0.061	1.69E-03	8.11E-03
cg04695882	16	20756332			S_Shelf	0.380	0.328	0.052	1.72E-03	8.20E-03
cg20824294	3	142316082	PLS1	5'UTR	S_Shore	0.582	0.520	0.062	1.72E-03	8.22E-03
cg20442379	10	60024634	IPMK	Body	N_Shelf	0.369	0.305	0.064	1.74E-03	8.27E-03
cg18888137	6	32945759	BRD2	Body	OpenSea	0.529	0.478	0.051	1.74E-03	8.27E-03
cg08571738	2	2605398			OpenSea	0.671	0.619	0.052	1.76E-03	8.32E-03
cg19587237	1	54797076	SSBP3	Body	OpenSea	0.409	0.336	0.074	1.76E-03	8.33E-03

cg15659420	11	20034979	NAV2	Body	OpenSea	0.431	0.372	0.059	1.77E-03	8.36E-03
cg19876987	3	14258049			OpenSea	0.625	0.676	-0.051	1.77E-03	8.36E-03
cg13740929	6	31651291			Island	0.643	0.581	0.063	1.78E-03	8.40E-03
cg14209920	19	13137642	NFIX	Body	S_Shore	0.804	0.748	0.057	1.78E-03	8.40E-03
cg16735495	7	105690812			OpenSea	0.588	0.531	0.057	1.80E-03	8.46E-03
cg21502048	14	52780158	PTGER2	TSS1500	N_Shore	0.385	0.334	0.051	1.80E-03	8.46E-03
cg02136596	1	1384930	ATAD3C	TSS200	OpenSea	0.745	0.688	0.056	1.82E-03	8.52E-03
cg01598009	6	30286162	HCG18	Body	OpenSea	0.339	0.285	0.054	1.83E-03	8.58E-03
cg10338518	4	87895137	AFF1	Body	OpenSea	0.586	0.527	0.059	1.84E-03	8.60E-03
cg07033722	1	40539032	PPT1	3'UTR	OpenSea	0.569	0.517	0.052	1.85E-03	8.62E-03
cg06578342	16	4349215			OpenSea	0.491	0.440	0.051	1.85E-03	8.62E-03
cg01327169	3	183738470			S_Shelf	0.703	0.650	0.053	1.86E-03	8.68E-03
cg17620335	10	51566824	NCOA4	5'UTR	S_Shore	0.711	0.655	0.057	1.88E-03	8.72E-03
cg07277038	3	196373543	LRRC33	5'UTR	OpenSea	0.595	0.535	0.059	1.88E-03	8.73E-03
cg09750084	13	49005868	LPAR6;RB1	5'UTR;Body	OpenSea	0.346	0.282	0.064	1.89E-03	8.76E-03
cg20166027	10	51576452	NCOA4	1stExon;5'UTR;Body	S_Shelf	0.621	0.566	0.054	1.90E-03	8.79E-03
cg12847536	8	1822186	ARHGEF10	Body	S_Shore	0.798	0.730	0.068	1.90E-03	8.79E-03
cg06312985	7	32961866	RP9P	Body	OpenSea	0.466	0.415	0.050	1.92E-03	8.85E-03
cg22226592	1	109439213	GPSM2	Body	OpenSea	0.669	0.608	0.061	1.95E-03	8.96E-03
cg03707599	7	75956931	YWHAG	3'UTR	N_Shore	0.290	0.228	0.062	1.96E-03	8.99E-03
cg15251256	12	46101447			OpenSea	0.735	0.680	0.055	1.97E-03	9.03E-03
cg03123541	1	95699097	RWDD3	TSS1500	N_Shore	0.544	0.479	0.064	1.98E-03	9.06E-03
cg16706260	12	27849370	REP15	TSS200	OpenSea	0.729	0.675	0.054	1.98E-03	9.06E-03
cg20861489	6	55105265	HCRTR2	Body	OpenSea	0.590	0.529	0.061	1.99E-03	9.07E-03
cg04697624	12	69002178			N_Shelf	0.413	0.361	0.052	1.99E-03	9.10E-03
cg17130518	7	76825482	CCDC146;FGL2	Body;3'UTR	OpenSea	0.851	0.789	0.061	2.00E-03	9.12E-03
cg18801945	19	13215041	LYL1	TSS1500	N_Shore	0.231	0.171	0.060	2.01E-03	9.14E-03
cg16267322	14	92864074	SLC24A4	Body	OpenSea	0.713	0.663	0.050	2.02E-03	9.17E-03
cg17451677	10	12887270			OpenSea	0.598	0.526	0.073	2.05E-03	9.26E-03
cg16542646	16	22175965			S_Shelf	0.848	0.790	0.059	2.05E-03	9.28E-03
cg21587238	5	42953590			S_Shore	0.666	0.592	0.074	2.06E-03	9.30E-03
cg01175610	12	12224246	BCL2L14	5'UTR;TSS200	OpenSea	0.611	0.674	-0.063	2.06E-03	9.30E-03
cg22062741	8	1897075	ARHGEF10	Body	S_Shelf	0.577	0.525	0.052	2.10E-03	9.43E-03
cg16145324	6	36020012	MAPK14	Body	OpenSea	0.684	0.626	0.058	2.10E-03	9.44E-03
cg07091500	3	185678074			OpenSea	0.355	0.289	0.065	2.12E-03	9.49E-03
cg17974145	7	101671556	CUX1	Body	OpenSea	0.617	0.561	0.055	2.14E-03	9.56E-03
cg19871394	6	11539786	TMEM170B	Body	S_Shore	0.758	0.707	0.051	2.14E-03	9.57E-03
cg22431093	12	32654992	FGD4	TSS200	OpenSea	0.620	0.569	0.051	2.18E-03	9.67E-03
cg14138235	14	56281968			OpenSea	0.507	0.455	0.053	2.18E-03	9.68E-03
cg01752594	13	50696070	DLEU2	Body	N_Shore	0.678	0.623	0.056	2.21E-03	9.78E-03
cg05919621	10	127373136	C10orf122	TSS1500	OpenSea	0.645	0.763	-0.119	2.23E-03	9.83E-03
cg06943912	19	10715612	SLC44A2	Body	S_Shelf	0.200	0.148	0.052	2.23E-03	9.85E-03
cg02054457	1	175683971	TNR	5'UTR	OpenSea	0.802	0.735	0.067	2.24E-03	9.88E-03
cg05377949	3	127951287	EEFSEC	Body	OpenSea	0.748	0.682	0.066	2.28E-03	9.98E-03
cg24020157	10	43697521	RASGEF1A	Body	N_Shore	0.370	0.320	0.050	2.30E-03	1.00E-02
cg24179445	10	102496915			N_Shore	0.507	0.457	0.050	2.34E-03	1.02E-02
cg25367558	6	41840102	USP49	5'UTR	OpenSea	0.730	0.676	0.054	2.34E-03	1.02E-02
cg16490906	5	93712596	C5orf36	Body	OpenSea	0.605	0.551	0.054	2.35E-03	1.02E-02

cg26104986	11	75275303	SERPINH1	5'UTR	S_Shore	0.735	0.684	0.051	2.37E-03	1.03E-02
cg01477971	2	103405951	TMEM182	Body	OpenSea	0.625	0.557	0.068	2.38E-03	1.03E-02
cg26780042	7	6515961	KDELR2	Body	OpenSea	0.681	0.614	0.066	2.40E-03	1.04E-02
cg07091102	1	94385022			OpenSea	0.743	0.687	0.056	2.43E-03	1.04E-02
cg10599438	19	844589	PRTN3	Body	N_Shore	0.291	0.232	0.059	2.44E-03	1.05E-02
cg01903702	1	224351941			OpenSea	0.394	0.341	0.053	2.44E-03	1.05E-02
cg00750430	10	118654150	KIAA1598	Body	OpenSea	0.447	0.392	0.056	2.44E-03	1.05E-02
cg18854765	1	74665362	LRRIQ3;TNNI3K;FPGT	TSS1500;Body;Body	S_Shore	0.425	0.361	0.065	2.45E-03	1.05E-02
cg24827600	2	169355957	LASS6	Body	OpenSea	0.552	0.502	0.050	2.47E-03	1.06E-02
cg11897887	20	23550632	CST9L	TSS1500	OpenSea	0.833	0.712	0.121	2.50E-03	1.06E-02
cg08087969	12	132657669			OpenSea	0.527	0.464	0.064	2.50E-03	1.06E-02
cg14016236	3	171792545	FNDC3B	5'UTR	OpenSea	0.494	0.424	0.070	2.50E-03	1.07E-02
cg08920032	15	99332004	IGF1R	Body	OpenSea	0.623	0.568	0.055	2.51E-03	1.07E-02
cg13589463	14	100920326	WDR25	Body	OpenSea	0.738	0.688	0.050	2.52E-03	1.07E-02
cg03322353	4	141069770	MAML3	Body	N_Shore	0.642	0.585	0.057	2.64E-03	1.11E-02
cg08930843	10	31182298	ZNF438	5'UTR;Body	OpenSea	0.604	0.549	0.055	2.64E-03	1.11E-02
cg09408143	16	524778	RAB11FIP3	Body;TSS200	OpenSea	0.561	0.510	0.052	2.66E-03	1.11E-02
cg09991306	2	241975140	SNED1	Body	Island	0.461	0.518	-0.058	2.67E-03	1.12E-02
cg06699564	8	95651099			N_Shore	0.479	0.425	0.053	2.67E-03	1.12E-02
cg08594554	17	72231318	TTYH2	Body	OpenSea	0.333	0.280	0.053	2.68E-03	1.12E-02
cg16095551	6	155816164			OpenSea	0.715	0.658	0.058	2.69E-03	1.12E-02
cg23118561	13	51565237			OpenSea	0.872	0.817	0.055	2.69E-03	1.12E-02
cg14886849	14	103367489	TRAF3	Body	OpenSea	0.369	0.315	0.054	2.69E-03	1.12E-02
cg06940110	2	118616430			N_Shore	0.564	0.513	0.051	2.69E-03	1.12E-02
cg20922701	18	11978319			N_Shelf	0.672	0.618	0.054	2.73E-03	1.13E-02
cg07543138	21	16434067	NRIP1	5'UTR	N_Shelf	0.362	0.307	0.055	2.74E-03	1.14E-02
cg01066472	1	75591029			Island	0.350	0.285	0.066	2.76E-03	1.14E-02
cg00809820	17	80708513	TBCD;FN3K	TSS1500;Body	Island	0.612	0.516	0.097	2.78E-03	1.15E-02
cg17797848	1	109842096	MYBPHL	Body	OpenSea	0.770	0.718	0.052	2.78E-03	1.15E-02
cg04476865	13	36223476	MIR548F5;NBEA	Body	OpenSea	0.738	0.687	0.051	2.79E-03	1.15E-02
cg17514528	1	11862907	MTHFR	Body	N_Shelf	0.562	0.503	0.059	2.81E-03	1.15E-02
cg22962883	11	114178729	NNMT	Body	OpenSea	0.410	0.341	0.069	2.83E-03	1.16E-02
cg14386624	9	91006749	SPIN1	5'UTR	S_Shelf	0.455	0.384	0.070	2.83E-03	1.16E-02
cg26874229	2	105853672			Island	0.511	0.456	0.055	2.83E-03	1.16E-02
cg08130265	15	77519170	C15orf5;SGK269	TSS1500;5'UTR	OpenSea	0.577	0.516	0.061	2.84E-03	1.16E-02
cg11906162	19	8568808	PRAM1	TSS1500	N_Shore	0.559	0.499	0.060	2.86E-03	1.17E-02
cg15958422	2	150845309			OpenSea	0.695	0.582	0.114	2.87E-03	1.17E-02
cg10348234	14	31698967			OpenSea	0.254	0.203	0.051	2.89E-03	1.18E-02
cg02322400	11	95980186	MAML2	Body	OpenSea	0.333	0.279	0.054	2.90E-03	1.18E-02
cg08599355	13	99714609	DOCK9	Body	OpenSea	0.581	0.528	0.053	2.93E-03	1.19E-02
cg16452651	21	35016873	ITSN1	5'UTR	S_Shore	0.596	0.542	0.054	2.95E-03	1.20E-02
cg27186013	4	95264127	HPGDS	TSS200	OpenSea	0.259	0.207	0.052	2.96E-03	1.20E-02
cg18802998	3	172444423			OpenSea	0.627	0.571	0.056	2.98E-03	1.20E-02
cg05194426	10	135343193	CYP2E1	Body	S_Shore	0.391	0.313	0.078	2.98E-03	1.20E-02
cg17403881	1	2847164			Island	0.926	0.854	0.072	2.99E-03	1.21E-02
cg01841050	17	39539863	KRT34	TSS1500	OpenSea	0.774	0.723	0.051	2.99E-03	1.21E-02
cg01044849	6	30002723	NCRNA00171	Body	OpenSea	0.621	0.571	0.050	3.02E-03	1.21E-02
cg14251267	20	62406428	ZBTB46	Body	N_Shore	0.714	0.662	0.051	3.02E-03	1.22E-02

cg18613721	12	8861225	RIMKLB	5'UTR	OpenSea	0.602	0.550	0.052	3.03E-03	1.22E-02
cg09326832	6	7243643	RREB1	Body	N_Shelf	0.250	0.198	0.051	3.04E-03	1.22E-02
cg22965561	10	13046134			S_Shelf	0.555	0.497	0.058	3.06E-03	1.23E-02
cg13160251	15	56286575	NEDD4	TSS1500	S_Shore	0.452	0.393	0.058	3.07E-03	1.23E-02
cg01952226	8	102507382	GRHL2	Body	S_Shore	0.350	0.284	0.066	3.13E-03	1.24E-02
cg07045054	3	10005827	TMEM111	Body	OpenSea	0.929	0.847	0.082	3.15E-03	1.25E-02
cg04414860	6	116575894	TSPYL4	TSS1500	S_Shore	0.451	0.397	0.054	3.16E-03	1.25E-02
cg05991492	16	3988700			N_Shore	0.499	0.440	0.059	3.19E-03	1.26E-02
cg06119452	6	169621662	THBS2	Body	N_Shore	0.637	0.578	0.058	3.19E-03	1.26E-02
cg20714551	19	3370244	NFIC;NFIC	Body	S_Shore	0.806	0.742	0.064	3.20E-03	1.26E-02
cg14372705	22	19141030			S_Shelf	0.651	0.599	0.052	3.20E-03	1.26E-02
cg04663285	11	379455	B4GALNT4	Body	Island	0.847	0.787	0.060	3.22E-03	1.27E-02
cg24475517	2	118737667	CCDC93	Body	OpenSea	0.581	0.527	0.054	3.23E-03	1.27E-02
cg02796279	2	208101334			OpenSea	0.558	0.503	0.055	3.24E-03	1.28E-02
cg07575193	8	30402570	RBPMS	Body	OpenSea	0.493	0.439	0.054	3.27E-03	1.28E-02
cg07958192	4	90033974	TIGD2	1stExon;5'UTR	S_Shore	0.716	0.654	0.063	3.30E-03	1.29E-02
cg18069568	18	43683922	ATP5A1;HAUS1	5'UTR;TSS1500	N_Shore	0.623	0.552	0.072	3.34E-03	1.30E-02
cg06358171	1	54822008	SSBP3	Body	Island	0.372	0.320	0.052	3.39E-03	1.32E-02
cg18278694	1	179945973	CEP350	5'UTR	OpenSea	0.564	0.503	0.061	3.41E-03	1.32E-02
cg20131219	3	111676660	PHLDB2	Body	OpenSea	0.517	0.389	0.128	3.43E-03	1.33E-02
cg22301128	4	77011716	ART3	Body	OpenSea	0.317	0.253	0.064	3.44E-03	1.33E-02
cg25179758	13	36918614	SPG20	5'UTR	N_Shore	0.440	0.385	0.054	3.45E-03	1.34E-02
cg21683390	18	13259088	C18orf1	5'UTR	N_Shelf	0.713	0.655	0.058	3.46E-03	1.34E-02
cg20384231	10	131132572			OpenSea	0.810	0.749	0.061	3.47E-03	1.34E-02
cg14076239	13	53712850			OpenSea	0.617	0.564	0.053	3.48E-03	1.34E-02
cg14018429	9	129138146	FAM125B	Body	OpenSea	0.494	0.424	0.070	3.48E-03	1.34E-02
cg18561976	2	204801508	ICOS;ICOS	5'UTR;1stExon	OpenSea	0.384	0.436	-0.053	3.49E-03	1.34E-02
cg11387340	6	166970727	RPS6KA2	Body	OpenSea	0.366	0.314	0.052	3.52E-03	1.35E-02
cg18729664	6	76203675	FILIP1	TSS200	OpenSea	0.681	0.630	0.051	3.56E-03	1.36E-02
cg03188948	7	1209495			OpenSea	0.222	0.163	0.058	3.56E-03	1.36E-02
cg26928972	3	122043799	CSTA	TSS1500	OpenSea	0.662	0.600	0.062	3.56E-03	1.36E-02
cg26567012	5	151202148	GLRA1	3'UTR	OpenSea	0.578	0.527	0.051	3.60E-03	1.37E-02
cg27422857	2	105853526			Island	0.667	0.601	0.066	3.61E-03	1.38E-02
cg10620484	4	122685570	LOC100192379;TMEM155	TSS200;5'UTR	N_Shore	0.295	0.240	0.055	3.63E-03	1.38E-02
cg18363918	19	51829984	IGLON5	Body	N_Shore	0.410	0.352	0.057	3.65E-03	1.39E-02
cg17864487	2	238643342	LRRFIP1	Body	N_Shore	0.761	0.711	0.050	3.67E-03	1.39E-02
cg01440934	1	47051752	MKNK1	Body	OpenSea	0.618	0.564	0.054	3.69E-03	1.40E-02
cg26646867	5	68785451			N_Shelf	0.602	0.547	0.055	3.70E-03	1.40E-02
cg23390684	15	32197095			OpenSea	0.763	0.705	0.058	3.71E-03	1.40E-02
cg12940181	2	145353012			OpenSea	0.507	0.448	0.059	3.71E-03	1.40E-02
cg26398228	4	33062845			OpenSea	0.551	0.465	0.086	3.72E-03	1.40E-02
cg21144063	7	64035529			OpenSea	0.341	0.287	0.054	3.75E-03	1.41E-02
cg18657389	17	74023630	EVPL	TSS200	OpenSea	0.682	0.626	0.056	3.76E-03	1.42E-02
cg07643696	12	25844221			OpenSea	0.522	0.464	0.058	3.78E-03	1.42E-02
cg27035169	14	23653098	SLC7A8	TSS1500	OpenSea	0.546	0.488	0.058	3.79E-03	1.42E-02
cg15531512	8	123706630			OpenSea	0.556	0.503	0.053	3.80E-03	1.43E-02
cg02343628	15	93790914			OpenSea	0.545	0.493	0.052	3.80E-03	1.43E-02
cg15912390	3	194826514	C3orf21	Body	OpenSea	0.794	0.742	0.053	3.82E-03	1.43E-02

cg21227325	9	78776599	PCSK5	Body	OpenSea	0.721	0.667	0.053	3.83E-03	1.44E-02
cg02915920	9	133736469	ABL1	Body	N_Shore	0.739	0.685	0.053	3.85E-03	1.44E-02
cg08473752	17	26517057	NLK	Body	OpenSea	0.765	0.713	0.053	3.85E-03	1.44E-02
cg11494508	7	92849025	HEPACAM2	TSS200;Body	OpenSea	0.292	0.238	0.054	3.86E-03	1.44E-02
cg13506281	13	29914200	MTUS2	Body	Island	0.402	0.525	-0.122	3.87E-03	1.45E-02
cg27076160	10	64431533	ZNF365	3'UTR	OpenSea	0.491	0.606	-0.115	3.88E-03	1.45E-02
cg02404304	6	2752090	MYLK4	TSS1500	OpenSea	0.355	0.297	0.059	3.92E-03	1.46E-02
cg18198212	5	39455917			OpenSea	0.347	0.291	0.056	3.93E-03	1.46E-02
cg05372753	6	11804635			OpenSea	0.677	0.625	0.053	3.94E-03	1.46E-02
cg02169713	6	31651094			Island	0.472	0.415	0.057	3.95E-03	1.47E-02
cg02278646	12	45608691	ANO6;PLEKHA9	5'UTR;TSS1500	N_Shore	0.403	0.346	0.056	3.95E-03	1.47E-02
cg14584535	9	125797187	GPR21;RABGAP1	1stExon;Body	OpenSea	0.528	0.464	0.064	3.99E-03	1.48E-02
cg05249836	22	45609402	C22orf9;C22orf9	Body;TSS1500	OpenSea	0.532	0.476	0.056	4.00E-03	1.48E-02
cg21493727	8	127938624			OpenSea	0.496	0.442	0.055	4.04E-03	1.49E-02
cg18167921	1	68963198	DEPDC1	TSS1500	S_Shore	0.448	0.389	0.059	4.05E-03	1.49E-02
cg17065772	3	14991718	NR2C2	5'UTR	S_Shore	0.607	0.551	0.056	4.05E-03	1.49E-02
cg24197477	1	202975221	TMEM183B;TMEM183A	TSS1500	N_Shore	0.757	0.703	0.054	4.05E-03	1.49E-02
cg19903071	2	160473251	BAZ2B	TSS200	S_Shore	0.328	0.265	0.063	4.10E-03	1.51E-02
cg15976390	1	205054828	TMEM81	TSS1500	OpenSea	0.937	0.881	0.056	4.15E-03	1.52E-02
cg23222472	7	112135961			OpenSea	0.454	0.397	0.057	4.15E-03	1.52E-02
cg04590170	11	33893895	LMO2	5'UTR	S_Shelf	0.587	0.535	0.051	4.19E-03	1.53E-02
cg00682263	15	66188803	MEGF11	3'UTR	OpenSea	0.634	0.564	0.070	4.25E-03	1.54E-02
cg23916104	11	7110083	RBMXL2	TSS200	N_Shore	0.592	0.537	0.055	4.27E-03	1.55E-02
cg11905488	12	10123604	CLEC12A	TSS1500	OpenSea	0.621	0.556	0.065	4.27E-03	1.55E-02
cg09365147	6	111738477	REV3L	Body	OpenSea	0.508	0.453	0.055	4.31E-03	1.56E-02
cg09572125	6	33400477	SYNGAP1	Body	OpenSea	0.319	0.259	0.059	4.31E-03	1.56E-02
cg19931348	20	43803224	PI3	TSS1500	OpenSea	0.687	0.632	0.055	4.32E-03	1.56E-02
cg06585566	19	57276794			Island	0.410	0.353	0.057	4.38E-03	1.58E-02
cg05362850	7	150100037			N_Shore	0.802	0.739	0.063	4.39E-03	1.58E-02
cg11540476	6	31651249			Island	0.525	0.432	0.093	4.53E-03	1.62E-02
cg04839673	1	64105555	PGM1	Body	OpenSea	0.945	0.877	0.068	4.54E-03	1.62E-02
cg19599611	15	35148637	AQR	3'UTR	OpenSea	0.753	0.694	0.059	4.54E-03	1.62E-02
cg25537993	19	58545182	ZSCAN1	TSS1500	Island	0.181	0.128	0.054	4.57E-03	1.63E-02
cg09422696	8	144626190			S_Shelf	0.778	0.678	0.101	4.62E-03	1.64E-02
cg02187259	2	16804452	FAM49A	5'UTR	OpenSea	0.614	0.564	0.050	4.63E-03	1.64E-02
cg11054816	11	117183853	BACE1	Body	N_Shelf	0.524	0.474	0.051	4.65E-03	1.65E-02
cg04605532	1	234843592			OpenSea	0.682	0.624	0.058	4.66E-03	1.65E-02
cg04182865	5	141346431	RNF14	TSS200	N_Shelf	0.635	0.574	0.060	4.83E-03	1.69E-02
cg06885823	5	82675005			OpenSea	0.577	0.525	0.052	4.83E-03	1.69E-02
cg23047825	15	99317340	IGF1R	Body	OpenSea	0.791	0.728	0.063	4.84E-03	1.70E-02
cg00625110	16	53741731	FTO	Body	S_Shelf	0.566	0.496	0.070	4.85E-03	1.70E-02
cg20760057	6	130017522	ARHGAP18	Body	OpenSea	0.639	0.580	0.060	4.86E-03	1.70E-02
cg15033269	3	42631489	SS18L2	TSS1500	N_Shore	0.545	0.494	0.051	4.88E-03	1.71E-02
cg21786601	7	116222436			OpenSea	0.890	0.837	0.053	4.92E-03	1.71E-02
cg18075755	4	15429967	C1QTNF7	5'UTR;Body;5'UTR	OpenSea	0.854	0.756	0.098	4.92E-03	1.72E-02
cg12303247	1	155853542	SYT11	3'UTR	OpenSea	0.952	0.883	0.070	4.94E-03	1.72E-02
cg18551877	11	58612087	GLYATL2	TSS200	OpenSea	0.191	0.128	0.063	4.94E-03	1.72E-02
cg27340480	19	44170526	PLAUR	Body	N_Shelf	0.530	0.470	0.061	4.96E-03	1.73E-02

cg26508200	12	109235071	SSH1	Body	OpenSea	0.680	0.621	0.059	5.01E-03	1.74E-02
cg14086364	5	150678334	SLC36A3	Body	OpenSea	0.603	0.549	0.054	5.04E-03	1.75E-02
cg21263566	1	95561502			OpenSea	0.551	0.497	0.055	5.06E-03	1.75E-02
cg24401441	20	21679843			N_Shelf	0.472	0.419	0.054	5.07E-03	1.75E-02
cg15723028	8	90776474	RIPK2	Body	OpenSea	0.545	0.485	0.060	5.08E-03	1.75E-02
cg01556706	3	59804435	FHIT	Body	OpenSea	0.753	0.690	0.063	5.15E-03	1.77E-02
cg11842502	6	111862202			OpenSea	0.593	0.532	0.061	5.24E-03	1.79E-02
cg00637104	20	1785303			S_Shore	0.555	0.504	0.050	5.26E-03	1.80E-02
cg03156546	16	24759640	TNRC6A	Body	OpenSea	0.647	0.586	0.061	5.26E-03	1.80E-02
cg17500686	15	79621783			OpenSea	0.700	0.645	0.055	5.27E-03	1.80E-02
cg08445957	8	1246463			Island	0.952	0.877	0.076	5.28E-03	1.80E-02
cg05126289	1	156995411	ARHGEF11	Body	OpenSea	0.889	0.826	0.063	5.28E-03	1.80E-02
cg01422009	16	125896	MPG	TSS1500	N_Shore	0.724	0.671	0.053	5.29E-03	1.81E-02
cg11143674	15	99069100			OpenSea	0.403	0.352	0.051	5.31E-03	1.81E-02
cg04313565	16	17561865	XYLT1	Body	OpenSea	0.513	0.461	0.051	5.32E-03	1.81E-02
cg21139795	3	119243933	CD80	3'UTR	OpenSea	0.581	0.524	0.057	5.34E-03	1.82E-02
cg13158272	14	74257174			S_Shelf	0.857	0.799	0.058	5.34E-03	1.82E-02
cg04677123	5	90466501			OpenSea	0.644	0.586	0.058	5.36E-03	1.82E-02
cg08700083	4	48382815	SLAIN2	Body	OpenSea	0.506	0.440	0.066	5.39E-03	1.83E-02
cg18789918	13	84452994	SLITRK1	3'UTR;1stExon	N_Shore	0.240	0.186	0.054	5.41E-03	1.83E-02
cg23216724	6	167571584	GPR31	TSS1500	OpenSea	0.611	0.559	0.053	5.42E-03	1.84E-02
cg12744694	8	89300240	MMP16	Body	OpenSea	0.785	0.677	0.107	5.47E-03	1.85E-02
cg06363243	16	1630430	IFT140	Body	OpenSea	0.669	0.618	0.051	5.47E-03	1.85E-02
cg13551117	2	111979398			N_Shore	0.515	0.459	0.057	5.52E-03	1.86E-02
cg01692842	11	134126364	ACAD8	Body	S_Shelf	0.627	0.568	0.059	5.63E-03	1.89E-02
cg09540738	8	81080281	TPD52	Body	N_Shelf	0.665	0.612	0.053	5.64E-03	1.89E-02
cg00898013	13	113819073	PROZ	Body	OpenSea	0.507	0.449	0.058	5.65E-03	1.89E-02
cg17846016	17	80181015			S_Shore	0.568	0.516	0.053	5.67E-03	1.90E-02
cg26161528	10	133208692			OpenSea	0.892	0.819	0.073	5.68E-03	1.90E-02
cg24967811	12	123503709	PITPNM2	Body	OpenSea	0.567	0.508	0.059	5.69E-03	1.90E-02
cg14120049	19	36246408	HSPB6	3'UTR	Island	0.507	0.437	0.070	5.70E-03	1.90E-02
cg11860238	10	134726665			N_Shelf	0.823	0.745	0.078	5.71E-03	1.91E-02
cg26930596	1	2082315	PRKCZ	Body	Island	0.550	0.499	0.051	5.72E-03	1.91E-02
cg01366985	6	25167695			OpenSea	0.256	0.197	0.059	5.74E-03	1.91E-02
cg24002158	8	142369468	GPR20	5'UTR	S_Shore	0.453	0.392	0.060	5.75E-03	1.92E-02
cg22905866	4	185468777			OpenSea	0.481	0.428	0.053	5.78E-03	1.93E-02
cg02538681	10	90611911	ANKRD22	TSS200	OpenSea	0.651	0.594	0.058	5.80E-03	1.93E-02
cg05089897	21	38886664	DYRK1A	3'UTR	OpenSea	0.769	0.717	0.052	5.83E-03	1.94E-02
cg13935765	9	89348726			OpenSea	0.718	0.575	0.143	5.86E-03	1.94E-02
cg04714076	9	132426059			N_Shore	0.713	0.645	0.068	5.94E-03	1.96E-02
cg26407841	7	81475720			OpenSea	0.459	0.400	0.059	5.95E-03	1.97E-02
cg16788857	3	192577951	C3orf59	Body	OpenSea	0.733	0.669	0.064	6.02E-03	1.98E-02
cg04360793	1	79472361	ELTD1	5'UTR;1stExon	Island	0.279	0.228	0.051	6.04E-03	1.98E-02
cg13852284	10	77548353	C10orf11	Body	OpenSea	0.635	0.583	0.053	6.05E-03	1.99E-02
cg10206397	20	2085344	STK35	Body	S_Shore	0.678	0.620	0.059	6.05E-03	1.99E-02
cg04256466	4	169752398	PALLD	Body;TSS1500	N_Shore	0.255	0.204	0.052	6.05E-03	1.99E-02
cg15642380	1	172908788			OpenSea	0.467	0.402	0.066	6.08E-03	2.00E-02
cg02862885	2	55327371			OpenSea	0.590	0.539	0.052	6.09E-03	2.00E-02

cg01674119	6	31583231	AIF1	TSS1500;5'UTR;Body	OpenSea	0.306	0.254	0.052	6.15E-03	2.01E-02
cg10071929	9	130955135	CIZ1	TSS1500;5'UTR	N_Shore	0.189	0.138	0.051	6.18E-03	2.02E-02
cg02583091	8	145638881	SLC39A4	Body	Island	0.360	0.282	0.077	6.22E-03	2.03E-02
cg10920224	14	103367591	TRAF3	Body	OpenSea	0.297	0.233	0.064	6.24E-03	2.03E-02
cg17369406	21	34918578	SON	Body	OpenSea	0.414	0.352	0.062	6.24E-03	2.03E-02
cg13801402	1	114429965	BCL2L15	1stExon	OpenSea	0.724	0.672	0.052	6.27E-03	2.04E-02
cg21927991	8	135494242	ZFAT	Body	S_Shelf	0.680	0.804	-0.124	6.28E-03	2.04E-02
cg18517055	17	80581701	WDR45L	Body	OpenSea	0.705	0.654	0.051	6.30E-03	2.05E-02
cg02394698	4	86594376	ARHGAP24	Body	OpenSea	0.391	0.336	0.055	6.36E-03	2.06E-02
cg20382675	3	71591429	FOXP1;MIR1284	5'UTR;TSS200	OpenSea	0.542	0.487	0.055	6.38E-03	2.06E-02
cg16045423	22	39377790	APOBEC3B	TSS1500	OpenSea	0.676	0.610	0.066	6.43E-03	2.08E-02
cg02107279	5	14870749	ANKH	Body	N_Shore	0.252	0.202	0.050	6.44E-03	2.08E-02
cg15929078	6	33267996	TAPBP;RGL2	3'UTR;TSS1500	S_Shore	0.279	0.222	0.056	6.45E-03	2.08E-02
cg01283625	10	13568099			N_Shelf	0.698	0.648	0.050	6.46E-03	2.08E-02
cg19699682	3	119349840			OpenSea	0.623	0.558	0.065	6.48E-03	2.09E-02
cg13378649	1	222927856			OpenSea	0.504	0.451	0.054	6.50E-03	2.09E-02
cg15027294	12	1609537	LOC100292680	TSS200	OpenSea	0.476	0.416	0.061	6.53E-03	2.10E-02
cg10342963	15	99443213	IGF1R	Body	OpenSea	0.759	0.709	0.050	6.58E-03	2.11E-02
cg24416910	12	132290859			N_Shelf	0.803	0.750	0.053	6.61E-03	2.12E-02
cg25457927	22	38595422			N_Shelf	0.579	0.525	0.054	6.62E-03	2.12E-02
cg13068698	7	35078082	DPY19L1	TSS1500	S_Shore	0.376	0.318	0.058	6.65E-03	2.13E-02
cg13221899	13	103426862	C13orf27	TSS1500	Island	0.414	0.355	0.059	6.67E-03	2.13E-02
cg00069771	1	233089275	C1orf57	Body	S_Shelf	0.853	0.739	0.114	6.68E-03	2.13E-02
cg22023435	16	89034099	CBFA2T3	Body	Island	0.388	0.333	0.055	6.70E-03	2.14E-02
cg21912448	1	8727007	RERE	5'UTR	OpenSea	0.401	0.338	0.063	6.72E-03	2.14E-02
cg03371275	11	95974543	MAML2	Body	OpenSea	0.690	0.639	0.051	6.72E-03	2.14E-02
cg04326337	20	36664969	RPRD1B	Body	S_Shelf	0.863	0.806	0.057	6.76E-03	2.15E-02
cg26233651	1	90137953	LRRC8C	5'UTR	OpenSea	0.899	0.837	0.061	6.80E-03	2.16E-02
cg00001593	1	170490434			OpenSea	0.919	0.851	0.069	6.82E-03	2.17E-02
cg16871435	10	65682643			OpenSea	0.717	0.662	0.055	6.84E-03	2.17E-02
cg07600533	22	50986031	KLHDC7B	TSS1500	Island	0.727	0.786	-0.059	6.90E-03	2.18E-02
cg04958045	17	13508158			S_Shelf	0.913	0.845	0.068	6.92E-03	2.19E-02
cg26426470	5	169181253	DOCK2	Body	OpenSea	0.935	0.873	0.062	6.94E-03	2.19E-02
cg11957400	12	2017093	CACNA2D4	Body	N_Shore	0.408	0.353	0.055	7.00E-03	2.21E-02
cg12869659	1	244213618	ZNF238	TSS1500	Island	0.201	0.151	0.050	7.10E-03	2.23E-02
cg18558006	14	69725842	GALNTL1	TSS1500	N_Shore	0.796	0.745	0.051	7.14E-03	2.24E-02
cg05216056	6	28887836	TRIM27	Body	N_Shelf	0.646	0.595	0.051	7.20E-03	2.25E-02
cg05681977	8	145638934	SLC39A4	Body	Island	0.303	0.239	0.064	7.31E-03	2.28E-02
cg24941342	11	95093809			OpenSea	0.523	0.470	0.053	7.32E-03	2.28E-02
cg05976283	8	103140765			S_Shelf	0.523	0.461	0.062	7.35E-03	2.29E-02
cg12209946	3	14987434			N_Shore	0.613	0.551	0.062	7.37E-03	2.29E-02
cg09001226	6	32155082	PBX2	Body	OpenSea	0.329	0.275	0.055	7.41E-03	2.30E-02
cg00636427	18	77190089	NFATC1	Body;5'UTR	N_Shelf	0.399	0.348	0.051	7.50E-03	2.32E-02
cg09874643	2	239362030			S_Shore	0.836	0.929	-0.093	7.50E-03	2.32E-02
cg16301036	12	118808298	TAOK3	5'UTR	N_Shore	0.620	0.563	0.058	7.50E-03	2.32E-02
cg07147033	1	1549615	MIB2	TSS1500	N_Shore	0.453	0.403	0.051	7.53E-03	2.33E-02
cg10454879	8	602158			S_Shore	0.805	0.745	0.061	7.55E-03	2.33E-02
cg23260484	1	245132134	EFCAB2	TSS1500	N_Shore	0.668	0.617	0.052	7.57E-03	2.34E-02

cg11993173	8	54857695	RGS20	Body	OpenSea	0.445	0.386	0.059	7.61E-03	2.34E-02
cg27170268	14	104171695	XRCC3	Body	OpenSea	0.579	0.522	0.057	7.63E-03	2.35E-02
cg14221851	12	129329181			N_Shore	0.745	0.694	0.051	7.70E-03	2.36E-02
cg03466780	9	140247365	EXD3	Body	N_Shore	0.759	0.665	0.094	7.70E-03	2.37E-02
cg23825057	12	124014409	RILPL1	Body	N_Shelf	0.632	0.580	0.052	7.72E-03	2.37E-02
cg26133399	12	62583974	FAM19A2	5'UTR	N_Shore	0.326	0.270	0.056	7.85E-03	2.40E-02
cg06033531	5	176980153	FAM193B	5'UTR;Body	N_Shore	0.566	0.514	0.052	7.89E-03	2.41E-02
cg05260346	6	158013621	ZDHHC14	Body	N_Shore	0.914	0.858	0.056	7.90E-03	2.41E-02
cg07502444	19	57682795			N_Shore	0.624	0.573	0.051	7.91E-03	2.41E-02
cg15234627	13	33837178	STARD13	Body	OpenSea	0.598	0.526	0.071	7.94E-03	2.42E-02
cg13072943	6	167011311	RPS6KA2	Body	OpenSea	0.619	0.568	0.051	7.96E-03	2.42E-02
cg09916840	16	87248612			N_Shore	0.460	0.319	0.141	8.00E-03	2.43E-02
cg07920381	12	132303685			OpenSea	0.596	0.541	0.056	8.29E-03	2.50E-02
cg27105914	17	76445798	DNAH17	Body	N_Shore	0.578	0.522	0.057	8.31E-03	2.50E-02
cg19303728	4	53759295	SCFD2	Body	OpenSea	0.895	0.817	0.078	8.32E-03	2.50E-02
cg24762231	12	54690793	NFE2	TSS1500;5'UTR	OpenSea	0.439	0.389	0.050	8.37E-03	2.51E-02
cg11341144	3	185656289	TRA2B	TSS1500	S_Shore	0.728	0.676	0.053	8.37E-03	2.51E-02
cg11343072	2	160473528	BAZ2B	TSS1500	S_Shore	0.524	0.465	0.059	8.44E-03	2.53E-02
cg21153102	15	41252147			OpenSea	0.502	0.442	0.061	8.45E-03	2.53E-02
cg11884704	9	130854313	SLC25A25	Body;1stExon	OpenSea	0.524	0.465	0.059	8.48E-03	2.54E-02
cg09976142	9	130955436	CIZ1	5'UTR	Island	0.296	0.236	0.060	8.49E-03	2.54E-02
cg23334433	17	79129051	AATK	Body	N_Shelf	0.618	0.554	0.064	8.54E-03	2.55E-02
cg03051617	17	48562276	RSAD1	3'UTR	OpenSea	0.885	0.787	0.097	8.60E-03	2.56E-02
cg22579028	22	27056901	MIAT	Body	S_Shelf	0.702	0.643	0.059	8.61E-03	2.57E-02
cg13460556	15	89709638	ABHD2	Body	OpenSea	0.628	0.574	0.054	8.61E-03	2.57E-02
cg03409151	5	125929671	ALDH7A1	Body	N_Shore	0.758	0.702	0.056	8.65E-03	2.57E-02
cg17111663	4	7647874	SORCS2	Body	Island	0.600	0.505	0.094	8.69E-03	2.58E-02
cg09101941	5	133775258			OpenSea	0.474	0.421	0.053	8.70E-03	2.59E-02
cg12924402	2	218898511			Island	0.582	0.672	-0.090	8.71E-03	2.59E-02
cg27545630	16	2013058	SNORA64;SNORA10;RPS2	Body;TSS1500	N_Shore	0.692	0.745	-0.052	8.73E-03	2.59E-02
cg20673721	17	25859382	KSR1	5'UTR	OpenSea	0.542	0.491	0.051	8.79E-03	2.60E-02
cg21696012	1	24131000	HMGCL	Body	S_Shelf	0.589	0.536	0.053	8.81E-03	2.61E-02
cg15254822	7	564137			S_Shore	0.807	0.756	0.051	8.83E-03	2.61E-02
cg17165241	11	400385	PKP3	Body	Island	0.596	0.546	0.050	8.95E-03	2.64E-02
cg27395066	17	43221220	ACBD4	3'UTR	N_Shore	0.259	0.208	0.052	8.98E-03	2.64E-02
cg13302785	3	51104051	DOCK3	Body	OpenSea	0.395	0.337	0.058	9.01E-03	2.65E-02
cg11180667	10	101415865			N_Shelf	0.551	0.493	0.058	9.11E-03	2.67E-02
cg16350446	2	172338747	DCAF17	Body;3'UTR	OpenSea	0.498	0.441	0.057	9.13E-03	2.68E-02
cg00114784	15	97322504			OpenSea	0.724	0.651	0.074	9.13E-03	2.68E-02
cg20435535	6	31651151			Island	0.359	0.280	0.079	9.13E-03	2.68E-02
cg25140190	18	13641529	C18orf1	Body	N_Shore	0.576	0.518	0.058	9.14E-03	2.68E-02
cg25416319	17	37080373			OpenSea	0.553	0.501	0.052	9.19E-03	2.69E-02
cg02138331	20	32893975	AHCY	5'UTR	S_Shelf	0.759	0.707	0.052	9.29E-03	2.71E-02
cg13488811	11	128685313			OpenSea	0.597	0.544	0.054	9.31E-03	2.72E-02
cg17679427	10	35480749	CREM	Body;5'UTR	OpenSea	0.523	0.467	0.056	9.31E-03	2.72E-02
cg13753351	9	127134207	PSMB7	Body	OpenSea	0.559	0.503	0.056	9.32E-03	2.72E-02
cg13870520	4	77131705	SCARB2	Body	N_Shelf	0.539	0.488	0.051	9.36E-03	2.73E-02
cg16323245	3	114622118	ZBTB20;ZBTB20	5'UTR	OpenSea	0.520	0.462	0.059	9.37E-03	2.73E-02

cg05302896	7	108524793	C7orf66	TSS200	OpenSea	0.818	0.766	0.052	9.50E-03	2.75E-02
cg11809958	12	32654929	FGD4	TSS200	OpenSea	0.693	0.638	0.055	9.51E-03	2.76E-02
cg10926851	5	146253830	PPP2R2B	Body;5'UTR	N_Shelf	0.610	0.742	-0.132	9.54E-03	2.76E-02
cg01942558	2	152214027	TNFAIP6	TSS200	OpenSea	0.714	0.658	0.056	9.57E-03	2.77E-02
cg23050394	5	123599727			OpenSea	0.620	0.568	0.051	9.58E-03	2.77E-02
cg27512707	6	9896436			OpenSea	0.935	0.882	0.053	9.59E-03	2.77E-02
cg22440848	4	184320416			S_Shore	0.502	0.448	0.053	9.62E-03	2.78E-02
cg16396191	13	42184765	KIAA0564	Body	OpenSea	0.754	0.702	0.051	9.66E-03	2.79E-02
cg27019030	14	75749992			S_Shelf	0.245	0.193	0.052	9.73E-03	2.80E-02
cg05522042	16	85124401	KIAA0513	3'UTR	OpenSea	0.671	0.605	0.066	9.74E-03	2.80E-02
cg27598107	2	102759782			S_Shore	0.586	0.533	0.053	9.74E-03	2.80E-02
cg08324090	17	7253189	ACAP1	Body	N_Shore	0.722	0.665	0.056	9.87E-03	2.83E-02
cg12725760	1	192873912			OpenSea	0.334	0.274	0.060	9.88E-03	2.83E-02
cg01175220	20	30617892	C20orf160	3'UTR	N_Shore	0.761	0.695	0.065	9.88E-03	2.84E-02
cg14774440	8	37730745	RAB11FIP1	Body;5'UTR;1stExon	OpenSea	0.459	0.408	0.050	9.91E-03	2.84E-02
cg20078972	19	15391832	BRD4;BRD4	TSS1500	OpenSea	0.485	0.431	0.055	9.91E-03	2.84E-02
cg26277026	12	22486029	ST8SIA1	Body	N_Shore	0.470	0.415	0.056	9.95E-03	2.85E-02
cg03202732	11	63655087	MARK2	TSS1500;Body	OpenSea	0.427	0.366	0.061	9.95E-03	2.85E-02
cg15930703	2	225775142	DOCK10	Body	OpenSea	0.731	0.673	0.057	1.00E-02	2.86E-02
cg13074055	14	106329206			OpenSea	0.504	0.453	0.051	1.00E-02	2.87E-02
cg06654773	11	68199794	LRP5	Body	N_Shore	0.843	0.792	0.052	1.01E-02	2.88E-02
cg19867917	2	3642629	COLEC11	TSS200	Island	0.488	0.432	0.056	1.01E-02	2.89E-02
cg16580197	8	67841925			S_Shelf	0.508	0.452	0.056	1.02E-02	2.91E-02
cg27504269	12	21524305	SLCO1A2;IAPP	5'UTR;TSS1500	OpenSea	0.854	0.906	-0.052	1.02E-02	2.91E-02
cg08173216	15	41524250	CHP;EXD1	Body;TSS1500	S_Shore	0.445	0.393	0.052	1.03E-02	2.93E-02
cg13642260	9	130955380	CIZ1	5'UTR	Island	0.215	0.164	0.050	1.04E-02	2.94E-02
cg05162456	16	85671457	KIAA0182	5'UTR;Body	S_Shore	0.858	0.795	0.064	1.04E-02	2.94E-02
cg11147866	15	68835517			OpenSea	0.722	0.666	0.056	1.04E-02	2.94E-02
cg09672452	15	55665236	CCPG1;MIR628;CCPG1	Body;TSS200	OpenSea	0.278	0.227	0.051	1.05E-02	2.97E-02
cg25489105	3	129155354	MBD4	Body	N_Shelf	0.432	0.376	0.056	1.06E-02	2.98E-02
cg17586302	6	144013969	PHACTR2	Body	OpenSea	0.602	0.550	0.052	1.06E-02	2.98E-02
cg23210971	4	88049275	AFF1	Body	OpenSea	0.743	0.691	0.051	1.06E-02	2.98E-02
cg13679804	3	59734950			OpenSea	0.523	0.461	0.062	1.06E-02	2.99E-02
cg11839815	3	127539037	MGLL	Body	N_Shelf	0.620	0.563	0.057	1.07E-02	3.00E-02
cg10611016	11	6225759	C11orf42	TSS1500	OpenSea	0.632	0.574	0.058	1.07E-02	3.00E-02
cg21892720	19	54877827	LAIR1;LAIR1	TSS1500	OpenSea	0.553	0.497	0.056	1.08E-02	3.03E-02
cg14713146	2	171782647			N_Shelf	0.651	0.600	0.050	1.09E-02	3.06E-02
cg00211174	3	127332098	MCM2	Body	OpenSea	0.532	0.475	0.056	1.10E-02	3.07E-02
cg21199093	1	35836009	ZMYM4	Body	OpenSea	0.573	0.510	0.063	1.11E-02	3.08E-02
cg16291589	20	6023178	LRRN4	Body	S_Shore	0.736	0.685	0.051	1.11E-02	3.08E-02
cg04563996	3	57093519	ARHGEF3;SPATA12	5'UTR;TSS1500	OpenSea	0.329	0.274	0.056	1.11E-02	3.08E-02
cg11916609	2	102927488	IL1RL1	TSS1500	OpenSea	0.655	0.601	0.054	1.11E-02	3.10E-02
cg21321372	4	141264456	SCOC	TSS200;5'UTR	OpenSea	0.372	0.322	0.050	1.11E-02	3.10E-02
cg03494634	19	9238958	OR7G3	TSS1500	OpenSea	0.261	0.177	0.084	1.13E-02	3.13E-02
cg02799427	16	8623289	TMEM114	TSS1500	S_Shelf	0.725	0.669	0.055	1.13E-02	3.13E-02
cg27019278	4	101438804	EMCN	Body	OpenSea	0.471	0.421	0.050	1.14E-02	3.15E-02
cg14804593	4	189139691			OpenSea	0.683	0.620	0.063	1.14E-02	3.16E-02
cg03018489	9	136397058	ADAMTSL2	TSS1500	N_Shelf	0.713	0.600	0.113	1.14E-02	3.16E-02

cg18984103	14	104163025	KLC1	Body	N_Shelf	0.556	0.499	0.057	1.15E-02	3.17E-02
cg06027691	3	37998565	CTDSPL	Body	OpenSea	0.347	0.287	0.060	1.15E-02	3.18E-02
cg10278149	2	208016923	KLF7	Body	OpenSea	0.768	0.716	0.052	1.16E-02	3.20E-02
cg08862479	5	177889945	COL23A1	Body	OpenSea	0.799	0.748	0.051	1.17E-02	3.20E-02
cg02206980	6	13574034	SIRT5;SIRT5	TSS1500	N_Shore	0.482	0.414	0.067	1.17E-02	3.22E-02
cg21762728	6	170165905	C6orf70	Body	OpenSea	0.751	0.693	0.057	1.18E-02	3.23E-02
cg00278392	6	16802505			OpenSea	0.547	0.493	0.054	1.18E-02	3.23E-02
cg22386583	17	78753756	RPTOR	Body	OpenSea	0.598	0.545	0.054	1.18E-02	3.24E-02
cg18558767	3	71592056	FOXP1;MIR1284	5'UTR;TSS1500	OpenSea	0.442	0.391	0.051	1.19E-02	3.25E-02
cg03940883	16	14380714			S_Shore	0.462	0.551	-0.089	1.19E-02	3.26E-02
cg02747563	7	157289477			N_Shelf	0.960	0.902	0.058	1.20E-02	3.27E-02
cg24740647	10	72681285			S_Shelf	0.589	0.529	0.060	1.21E-02	3.29E-02
cg21386573	1	94219800			OpenSea	0.706	0.655	0.051	1.21E-02	3.30E-02
cg13387643	1	10737562	CASZ1	Body	OpenSea	0.566	0.468	0.098	1.21E-02	3.30E-02
cg10474881	6	114181686	MARCKS	Body	Island	0.800	0.857	-0.057	1.22E-02	3.30E-02
cg14419424	10	65388604			N_Shore	0.257	0.201	0.056	1.23E-02	3.33E-02
cg00093643	15	44579510	CASC4	TSS1500	N_Shore	0.543	0.489	0.054	1.23E-02	3.33E-02
cg26300135	1	3324243	PRDM16	Body	S_Shore	0.904	0.835	0.069	1.23E-02	3.34E-02
cg01832662	14	86086389	FLRT2	5'UTR	OpenSea	0.231	0.173	0.058	1.24E-02	3.34E-02
cg01997599	19	49689673	TRPM4	Body	S_Shelf	0.368	0.288	0.080	1.24E-02	3.36E-02
cg09727050	2	152214177	TNFAIP6;TNFAIP6	5'UTR;1stExon	OpenSea	0.640	0.579	0.061	1.25E-02	3.37E-02
cg03741458	2	200468445			N_Shore	0.504	0.446	0.058	1.25E-02	3.38E-02
cg17750572	10	2544120			Island	0.653	0.587	0.065	1.26E-02	3.39E-02
cg01800262	1	54360170	DIO1	1stExon;1stExon;Body;1stExon	OpenSea	0.932	0.876	0.056	1.26E-02	3.40E-02
cg14835981	7	752715	PRKAR1B	TSS200;5'UTR;TSS1500;1stExon	S_Shore	0.498	0.444	0.053	1.27E-02	3.41E-02
cg16346422	11	2920819	SLC22A18;SLC22A18AS	TSS200;Body	N_Shelf	0.490	0.435	0.055	1.27E-02	3.42E-02
cg02853355	16	85132373	FAM92B	3'UTR	OpenSea	0.635	0.582	0.054	1.28E-02	3.44E-02
cg02175321	11	103930844	PDGFD	Body	OpenSea	0.558	0.506	0.052	1.29E-02	3.44E-02
cg00883212	2	173795201	RAPGEF4	Body	OpenSea	0.649	0.597	0.052	1.30E-02	3.47E-02
cg07283015	18	22039857	HRH4	TSS1500	OpenSea	0.614	0.563	0.051	1.30E-02	3.47E-02
cg00614011	8	120436479	NOV	3'UTR	OpenSea	0.812	0.742	0.070	1.30E-02	3.48E-02
cg22579075	7	94250352	SGCE	Body	OpenSea	0.653	0.708	-0.055	1.31E-02	3.49E-02
cg18673341	7	22481962	MGC87042	Body	OpenSea	0.675	0.577	0.098	1.31E-02	3.50E-02
cg17547295	5	157282503	CLINT1	Body	N_Shelf	0.594	0.541	0.053	1.31E-02	3.50E-02
cg15727583	3	196757701	MFI2	TSS1500	S_Shore	0.822	0.751	0.072	1.31E-02	3.50E-02
cg18926797	14	69523229	DCAF5	Body	OpenSea	0.618	0.563	0.055	1.32E-02	3.50E-02
cg08161546	6	152011415	ESR1	TSS1500	OpenSea	0.679	0.624	0.055	1.32E-02	3.51E-02
cg11100795	5	77583915	AP3B1	Body	OpenSea	0.652	0.601	0.051	1.32E-02	3.51E-02
cg04264638	4	56411138	CLOCK	5'UTR	N_Shore	0.343	0.285	0.059	1.32E-02	3.51E-02
cg02806156	12	89728752			OpenSea	0.572	0.516	0.057	1.33E-02	3.53E-02
cg07903626	16	66098650			OpenSea	0.685	0.586	0.100	1.33E-02	3.53E-02
cg10758676	6	158010406	ZDHHC14	Body	N_Shelf	0.818	0.914	-0.095	1.33E-02	3.53E-02
cg25308322	13	86268291			OpenSea	0.541	0.470	0.071	1.34E-02	3.55E-02
cg05755715	2	21646627			OpenSea	0.644	0.535	0.109	1.35E-02	3.56E-02
cg19193595	15	67396487	SMAD3	Body	OpenSea	0.391	0.322	0.069	1.35E-02	3.58E-02
cg10881162	3	55613558	ERC2	3'UTR	OpenSea	0.571	0.493	0.079	1.36E-02	3.59E-02
cg06255004	9	139580799	AGPAT2	Body	N_Shore	0.425	0.368	0.057	1.36E-02	3.59E-02
cg25945642	3	143321098	SLC9A9	Body	OpenSea	0.605	0.551	0.055	1.38E-02	3.64E-02

cg01057573	5	118683890	TNFAIP8	Body	OpenSea	0.571	0.518	0.053	1.39E-02	3.64E-02
cg22774605	6	47247047	TNFRSF21	Body	OpenSea	0.680	0.629	0.051	1.39E-02	3.65E-02
cg13814396	2	65090621			N_Shore	0.504	0.453	0.051	1.39E-02	3.65E-02
cg11463428	15	32607260			Island	0.782	0.708	0.074	1.40E-02	3.66E-02
cg05025071	19	6887530	EMR1	TSS200	OpenSea	0.588	0.529	0.059	1.40E-02	3.67E-02
cg01655008	14	93652954	C14orf109	Body	S_Shore	0.713	0.658	0.054	1.40E-02	3.67E-02
cg21932814	3	122044172	CSTA	1stExon	OpenSea	0.538	0.484	0.054	1.41E-02	3.69E-02
cg11716267	2	24367828	LOC375190	Body	OpenSea	0.539	0.665	-0.126	1.42E-02	3.70E-02
cg19677302	12	33048259	PKP2	Body	N_Shore	0.711	0.656	0.055	1.42E-02	3.70E-02
cg01561719	10	90611855	ANKRD22	TSS200	OpenSea	0.493	0.443	0.050	1.42E-02	3.70E-02
cg00168694	21	40193056	ETS2	Body	N_Shore	0.528	0.474	0.053	1.43E-02	3.72E-02
cg03115019	17	80708279	FN3K	Body	Island	0.863	0.812	0.050	1.43E-02	3.73E-02
cg13071618	8	144599125	ZC3H3	Body	N_Shelf	0.449	0.374	0.075	1.44E-02	3.74E-02
cg08185095	8	74527135	STAU2	Body	OpenSea	0.687	0.629	0.058	1.45E-02	3.76E-02
cg06627617	2	9471179	ASAP2;ASAP2	Body	OpenSea	0.238	0.185	0.052	1.45E-02	3.77E-02
cg24914483	18	2653997			N_Shore	0.583	0.517	0.067	1.45E-02	3.77E-02
cg22851875	5	65888284			N_Shelf	0.184	0.076	0.107	1.46E-02	3.77E-02
cg01219135	7	158766336			Island	0.737	0.677	0.060	1.46E-02	3.78E-02
cg09231171	16	49518414			OpenSea	0.314	0.259	0.055	1.47E-02	3.79E-02
cg00254095	18	47016218	RPL17;SNORD58C;U58;RPL17	Body;TSS1500	N_Shelf	0.352	0.478	-0.126	1.47E-02	3.81E-02
cg12488187	12	65671664	MSRB3	TSS1500	N_Shore	0.660	0.605	0.055	1.48E-02	3.82E-02
cg24550026	1	114430031	BCL2L15	1stExon;5'UTR	OpenSea	0.557	0.494	0.063	1.49E-02	3.84E-02
cg26836402	16	87252649			S_Shore	0.630	0.562	0.068	1.49E-02	3.84E-02
cg13216331	1	156025757	ROBLD3	Body	S_Shore	0.454	0.394	0.061	1.49E-02	3.84E-02
cg25654653	6	133035266	VNN1	TSS200	OpenSea	0.311	0.255	0.057	1.50E-02	3.85E-02
cg13286116	11	13302098	ARNTL	5'UTR	S_Shore	0.490	0.434	0.057	1.50E-02	3.85E-02
cg00760729	2	161236088	RBMS1	Body	OpenSea	0.478	0.418	0.060	1.50E-02	3.86E-02
cg07681748	3	112053517	CD200	Body	S_Shore	0.479	0.419	0.059	1.51E-02	3.87E-02
cg16615357	5	135416594	MIR886	TSS1500	S_Shore	0.536	0.478	0.058	1.52E-02	3.90E-02
cg06493806	18	77278806	NFATC1	Body	N_Shore	0.881	0.799	0.082	1.53E-02	3.91E-02
cg13333954	12	1098663			N_Shore	0.506	0.455	0.051	1.53E-02	3.92E-02
cg15837233	2	162279324	TBR1	Body	N_Shore	0.431	0.380	0.050	1.54E-02	3.94E-02
cg03313271	13	113913094	CUL4A	Body	N_Shelf	0.715	0.662	0.053	1.55E-02	3.96E-02
cg00078996	12	132293329			N_Shore	0.677	0.619	0.057	1.55E-02	3.96E-02
cg20704555	7	28127748	JAZF1	Body	OpenSea	0.664	0.611	0.054	1.57E-02	3.98E-02
cg02304584	6	31650790			Island	0.414	0.339	0.075	1.57E-02	3.99E-02
cg21517812	11	125443136	EI24;EI24	Body	S_Shelf	0.877	0.798	0.079	1.57E-02	3.99E-02
cg07437923	16	4506231	DNAJA3	3'UTR	OpenSea	0.553	0.433	0.120	1.57E-02	4.00E-02
cg01925738	5	140480770	PCDHB3	1stExon	N_Shore	0.507	0.456	0.051	1.59E-02	4.03E-02
cg14520947	1	225942842			OpenSea	0.588	0.536	0.052	1.59E-02	4.03E-02
cg26792295	3	16540091	RFTN1	5'UTR	OpenSea	0.634	0.579	0.055	1.60E-02	4.05E-02
cg01323964	7	65219171	SNORA22;CCT6P1	TSS1500;Body	S_Shelf	0.627	0.569	0.057	1.60E-02	4.05E-02
cg14762436	7	24917750	OSBPL3	Body	OpenSea	0.502	0.452	0.050	1.61E-02	4.06E-02
cg10862468	10	135342218	CYP2E1	Body	Island	0.272	0.221	0.051	1.61E-02	4.07E-02
cg07930587	6	32823566	PSMB9	Body	S_Shore	0.653	0.745	-0.092	1.62E-02	4.09E-02
cg16101346	1	186650479	PTGS2	TSS1500	S_Shore	0.469	0.412	0.057	1.63E-02	4.10E-02
cg21727359	6	82459006	FAM46A	3'UTR	N_Shelf	0.542	0.486	0.055	1.63E-02	4.10E-02
cg25576997	14	56257750	C14orf34	Body	OpenSea	0.631	0.572	0.058	1.63E-02	4.11E-02

cg06784232	8	19463150	CSGALNACT1	Body;5'UTR	S_Shelf	0.687	0.635	0.052	1.64E-02	4.12E-02
cg04118119	13	47371987	ESD	TSS1500	S_Shore	0.584	0.533	0.050	1.64E-02	4.12E-02
cg02275784	11	85242554	DLG2	Body	OpenSea	0.721	0.670	0.052	1.65E-02	4.13E-02
cg23464284	6	166996837	RPS6KA2	Body	OpenSea	0.532	0.457	0.076	1.67E-02	4.18E-02
cg03594078	8	22131675	PIWIL2;PIWIL2	TSS1500	N_Shore	0.617	0.564	0.053	1.68E-02	4.20E-02
cg00027155	16	11099199	CLEC16A	Body	OpenSea	0.765	0.667	0.098	1.68E-02	4.20E-02
cg12319143	10	134726771			N_Shelf	0.537	0.452	0.085	1.68E-02	4.20E-02
cg21491967	6	156276515			OpenSea	0.418	0.365	0.053	1.68E-02	4.20E-02
cg18170989	13	49975383	CAB39L	5'UTR;5'UTR;1stExon	OpenSea	0.435	0.378	0.056	1.69E-02	4.22E-02
cg09199225	6	32149260	AGER	Body	OpenSea	0.813	0.732	0.082	1.69E-02	4.22E-02
cg11956442	4	39531927			S_Shelf	0.569	0.441	0.128	1.69E-02	4.22E-02
cg07240846	10	12438782	CAMK1D	Body	OpenSea	0.893	0.829	0.064	1.70E-02	4.24E-02
cg14280181	2	223291007	SGPP2	Body	S_Shore	0.421	0.363	0.058	1.70E-02	4.24E-02
cg06975018	6	131894358	ARG1	TSS200	OpenSea	0.632	0.556	0.077	1.70E-02	4.24E-02
cg18105134	13	113819100	PROZ	Body	OpenSea	0.751	0.676	0.075	1.71E-02	4.25E-02
cg06398138	11	132912368	OPCML	Body	OpenSea	0.829	0.771	0.058	1.71E-02	4.25E-02
cg08600378	13	28562900	PRHOXNB	TSS200	OpenSea	0.731	0.673	0.058	1.71E-02	4.25E-02
cg07629625	10	133961825	JAKMIP3	Body	OpenSea	0.529	0.604	-0.075	1.72E-02	4.27E-02
cg12866960	12	66635398	IRAK3	Body	OpenSea	0.624	0.571	0.053	1.73E-02	4.29E-02
cg18728025	4	121991645	C4orf31	5'UTR	N_Shore	0.325	0.272	0.053	1.74E-02	4.30E-02
cg24498885	13	45770307	KCTD4;GTF2F2	5'UTR;Body	OpenSea	0.608	0.558	0.050	1.75E-02	4.33E-02
cg00994804	21	36259383	RUNX1	1stExon;Body	Island	0.246	0.304	-0.058	1.75E-02	4.33E-02
cg02470008	7	2648343	IQCE	Body	S_Shelf	0.351	0.298	0.053	1.75E-02	4.34E-02
cg06939451	5	81043172	SSBP2	Body	N_Shelf	0.550	0.496	0.054	1.77E-02	4.36E-02
cg04525852	4	125632538	ANKRD50	5'UTR	N_Shore	0.355	0.303	0.052	1.77E-02	4.36E-02
cg05608790	7	69427388	AUTS2	Body	OpenSea	0.460	0.406	0.054	1.77E-02	4.37E-02
cg19603903	19	57742345	AURKC	TSS200	Island	0.680	0.627	0.053	1.77E-02	4.38E-02
cg12296550	6	32728862	HLA-DQB2	Body	N_Shore	0.784	0.733	0.051	1.78E-02	4.39E-02
cg12656896	6	8438818			S_Shelf	0.447	0.395	0.053	1.78E-02	4.39E-02
cg00382999	3	136649333	NCK1	Body	OpenSea	0.598	0.539	0.059	1.79E-02	4.40E-02
cg21953058	15	41309253	INO80	Body	OpenSea	0.500	0.449	0.051	1.79E-02	4.40E-02
cg14754555	2	157292018	GPD2	1stExon;TSS1500;5'UTR	N_Shore	0.424	0.365	0.059	1.79E-02	4.41E-02
cg14873022	4	146841701	ZNF827	Body	OpenSea	0.727	0.671	0.056	1.79E-02	4.41E-02
cg03348161	11	59824089	MS4A3	TSS200	OpenSea	0.535	0.483	0.052	1.80E-02	4.42E-02
cg07734159	10	75772918	VCL;VCL	Body	OpenSea	0.715	0.658	0.057	1.80E-02	4.42E-02
cg23445003	7	101535821	CUX1	Body	OpenSea	0.575	0.515	0.061	1.80E-02	4.43E-02
cg15553418	1	16696547	C1orf144	Body	S_Shelf	0.511	0.458	0.052	1.83E-02	4.47E-02
cg06160853	3	174155855			N_Shelf	0.577	0.520	0.057	1.83E-02	4.48E-02
cg21263605	1	166818686	POGK	Body	OpenSea	0.912	0.858	0.054	1.83E-02	4.48E-02
cg08347373	2	160653686	CD302	Body	N_Shore	0.565	0.507	0.059	1.84E-02	4.48E-02
cg08154963	17	33426885	RAD51L3	3'UTR	OpenSea	0.530	0.477	0.053	1.84E-02	4.49E-02
cg10530344	15	81453270			OpenSea	0.433	0.325	0.108	1.89E-02	4.58E-02
cg01521397	20	60590872	TAF4	Body	S_Shelf	0.684	0.631	0.053	1.91E-02	4.61E-02
cg00691123	3	11632974	VGLL4	Body	OpenSea	0.573	0.521	0.052	1.93E-02	4.66E-02
cg15169266	7	41376887			OpenSea	0.712	0.661	0.052	1.94E-02	4.68E-02
cg24166916	5	78282669	ARSB	TSS1500	S_Shore	0.135	0.188	-0.053	1.95E-02	4.69E-02
cg01742370	1	228352412	C1orf69	TSS1500	N_Shore	0.705	0.651	0.054	1.95E-02	4.69E-02
cg18876706	10	31107995			OpenSea	0.849	0.780	0.068	1.95E-02	4.69E-02

cg00956142	1	28765031	PHACTR4	Body	OpenSea	0.512	0.459	0.053	1.98E-02	4.75E-02
cg12192749	7	17029795			OpenSea	0.566	0.513	0.053	1.99E-02	4.76E-02
cg19951200	3	141071669	ZBTB38	5'UTR	OpenSea	0.844	0.783	0.061	1.99E-02	4.76E-02
cg24492778	15	57414740	TCF12	Body	OpenSea	0.455	0.394	0.061	1.99E-02	4.77E-02
cg19636224	1	44176844	ST3GAL3	5'UTR	S_Shelf	0.689	0.630	0.058	2.00E-02	4.79E-02
cg08539965	1	21396338	EIF4G3	5'UTR	OpenSea	0.640	0.580	0.060	2.01E-02	4.80E-02
cg14312439	3	46283902	CCR3	1stExon;5'UTR	OpenSea	0.513	0.460	0.053	2.01E-02	4.81E-02
cg03847895	12	56652945	ANKRD52	TSS1500	S_Shore	0.437	0.385	0.051	2.02E-02	4.82E-02
cg26677549	2	236398235			N_Shelf	0.872	0.821	0.051	2.04E-02	4.85E-02
cg25084760	13	100886412	PCCA	Body	OpenSea	0.674	0.618	0.056	2.05E-02	4.86E-02
cg11876048	17	55337654	MSI2;MSI2	Body	S_Shelf	0.431	0.378	0.053	2.05E-02	4.87E-02
cg18624102	19	39523840	FBXO27	TSS1500	S_Shore	0.620	0.732	-0.112	2.06E-02	4.88E-02
cg16744531	19	17905626	B3GNT3	TSS1500	Island	0.473	0.415	0.058	2.06E-02	4.89E-02
cg05492904	15	51604503	CYP19A1	5'UTR	OpenSea	0.627	0.577	0.050	2.08E-02	4.92E-02
cg18025838	17	79232329	SLC38A10	Body	N_Shelf	0.863	0.805	0.059	2.09E-02	4.94E-02
cg08628635	6	20483859	E2F3	Body	OpenSea	0.569	0.517	0.052	2.09E-02	4.95E-02
cg26303777	1	230311676	GALNT2	Body	OpenSea	0.619	0.736	-0.117	2.10E-02	4.96E-02
cg21574244	15	42568411	GANC	Body	S_Shelf	0.653	0.595	0.058	2.10E-02	4.97E-02
cg10968027	11	95405470			OpenSea	0.448	0.388	0.060	2.11E-02	4.97E-02
cg10068464	2	114036953	PAX8;LOC440839	TSS1500	S_Shore	0.783	0.691	0.092	2.11E-02	4.98E-02
cg03714676	8	48174649	KIAA0146	Body	S_Shore	0.634	0.581	0.053	2.11E-02	4.98E-02
cg17572191	11	532979	HRAS	Body;3'UTR	N_Shore	0.889	0.829	0.060	2.12E-02	5.00E-02

Table S2. Selected differentially methylated regions (DMRs) (N=315) associated with HIV after adjustment for age, sex, and cell type with |MaxΔβ| >0.05, Stouffer<0.05, and containing at least 2 CpG sites, sorted by Stouffer from smallest to largest

CHR	hg19 genomic coordinates	Number of CpG probes	Minimal p-value	Stouffer	Maximal β change	Mean β change	Genes associated to region
6	chr6:33128825-33155135	169	6.05E-33	1.07E-134	0.061	-0.011	COL11A2
6	chr6:32184296-32191895	46	1.01E-44	3.34E-67	0.056	-0.013	NOTCH4
1	chr1:3141992-3144710	11	5.31E-27	2.68E-31	-0.059	-0.020	NA
6	chr6:170590562-170601120	44	7.94E-20	4.65E-28	-0.098	-0.010	DLL1, FAM120B
5	chr5:140682503-140684561	14	5.95E-36	1.83E-27	-0.051	-0.027	SLC25A2
6	chr6:30874989-30886161	93	5.79E-36	1.03E-25	-0.060	-0.006	GTF2H4, VARS2, RN7SL175P
6	chr6:31021134-31027005	21	9.94E-18	2.03E-24	-0.065	-0.018	HCG22
15	chr15:97319911-97323132	13	3.97E-17	3.89E-22	-0.073	-0.020	SPATA8-AS1
6	chr6:32819921-32823941	53	2.06E-22	5.40E-20	0.062	0.005	TAP1, PSMB9
17	chr17:46651186-46658170	46	5.91E-13	1.58E-19	-0.055	0.006	HOXB3, HOXB4, MIR10A
20	chr20:62569104-62572875	9	6.87E-14	4.93E-19	-0.056	-0.021	MIR647, UCKL1, MIR1914
1	chr1:228545604-228548842	9	4.04E-17	3.69E-18	-0.061	-0.020	NA
1	chr1:163392482-163393693	5	3.02E-25	7.46E-18	-0.051	-0.016	RP11-408E1.1
8	chr8:1616111-1618448	12	4.21E-23	9.06E-18	-0.066	-0.020	NA
3	chr3:138762690-138764086	9	1.10E-19	9.33E-17	-0.062	-0.033	PRR23C
22	chr22:33195343-33198055	17	1.23E-18	1.28E-16	-0.050	-0.007	TIMP3
20	chr20:2728576-2731560	8	1.46E-23	1.68E-16	-0.161	-0.046	EBF4
2	chr2:3641709-3643682	13	5.68E-18	4.45E-16	-0.057	-0.030	COLEC11
2	chr2:168673805-168675357	4	1.39E-26	4.81E-15	-0.050	-0.016	B3GALT1
7	chr7:27159883-27164709	18	1.84E-10	1.20E-14	0.056	-0.004	HOXA3, HOXA-AS2
3	chr3:141495473-141497467	15	3.26E-14	1.80E-14	-0.050	-0.011	GRK7
6	chr6:31837439-31839025	9	5.25E-19	9.90E-14	-0.057	-0.019	SLC44A4
8	chr8:75735552-75737456	5	1.51E-15	1.35E-13	-0.051	-0.026	PI15
16	chr16:1492791-1502837	28	4.07E-07	1.59E-13	-0.050	-0.008	CCDC154, LA16c-390E6.4, CLCN7
6	chr6:30954150-30957462	18	5.87E-17	3.08E-13	0.066	-0.004	NA
2	chr2:105852798-105854051	9	3.71E-13	3.98E-13	-0.073	-0.042	NA
6	chr6:32148943-32152269	23	9.06E-17	4.34E-13	-0.099	-0.011	AGER, XXbac-BPG300A18.13
4	chr4:122684903-122687552	17	1.95E-11	6.21E-13	-0.063	-0.012	TMEM155
6	chr6:119470324-119471177	5	1.70E-21	6.49E-13	-0.056	-0.017	FAM184A
2	chr2:106054920-106055507	6	9.52E-17	1.39E-12	-0.071	-0.024	FHL2
19	chr19:11784246-11785248	14	3.44E-15	1.85E-12	-0.051	-0.019	NA
3	chr3:196754322-196758146	13	1.25E-07	1.85E-12	-0.065	-0.007	MFI2
2	chr2:220402771-220409543	19	2.29E-11	2.56E-12	-0.052	-0.009	CHPF, TMEM198
11	chr11:45671015-45674702	10	1.44E-10	3.35E-12	-0.051	-0.017	RP11-495O11.1, CHST1
16	chr16:85669462-85672175	12	3.15E-12	4.46E-12	-0.062	-0.012	NA
13	chr13:76430089-76430722	4	1.54E-14	4.89E-12	-0.053	-0.029	LMO7
6	chr6:31650735-31651362	20	2.84E-13	5.67E-12	-0.065	-0.038	LY6G5C
8	chr8:1244039-1247803	8	6.07E-10	6.27E-12	-0.070	-0.013	NA
17	chr17:79231688-79236161	15	3.28E-08	6.60E-12	-0.056	-0.015	NA
10	chr10:135340445-135343280	16	6.26E-09	1.46E-11	-0.075	-0.036	CYP2E1, AL161645.2

8	chr8:144398248-144401094	11	4.61E-08	4.02E-11	-0.073	-0.012	TOP1MT-013, AC087793.1-201
1	chr1:1371844-1377656	16	1.26E-11	4.14E-11	-0.051	-0.013	VWA1, RP4-758J18.10
6	chr6:32728862-32731127	23	4.09E-16	9.30E-11	-0.053	-0.017	HLA-DQB2
8	chr8:1109986-1113811	14	9.56E-08	1.43E-10	-0.082	-0.018	NA
7	chr7:26192756-26193805	6	1.70E-12	1.63E-10	0.053	0.026	NFE2L3-001
7	chr7:157369121-157372332	15	2.22E-14	1.99E-10	-0.072	-0.014	NA
4	chr4:187984412-187985472	6	2.04E-15	2.02E-10	-0.054	-0.023	NA
13	chr13:46275352-46277286	5	5.31E-20	2.73E-10	-0.052	-0.020	SPERT
16	chr16:4027502-4029363	10	1.86E-07	3.28E-10	0.051	-0.001	NA
5	chr5:404678-405986	8	9.55E-14	4.17E-10	-0.060	-0.011	CTD-2228K2.2-001
21	chr21:43441482-43442940	9	7.98E-14	5.82E-10	-0.086	-0.021	ZNF295-AS1
22	chr22:25594918-25595955	10	3.91E-12	6.65E-10	-0.050	-0.014	CRYBB3
1	chr1:15255231-15256481	6	1.16E-12	7.36E-10	-0.054	-0.020	KAZN
2	chr2:31637622-31638511	3	1.74E-21	8.80E-10	-0.061	-0.025	XDH
17	chr17:80004741-80012647	28	9.40E-06	1.01E-09	-0.052	-0.006	GPS1, RFNG
3	chr3:18484742-18486958	8	4.11E-15	1.93E-09	-0.067	-0.008	AC144521.1, TBC1D5, SATB1
20	chr20:2673003-2675996	8	1.21E-15	2.22E-09	-0.098	-0.028	AL035460.1, EBF4
16	chr16:57022486-57023715	9	6.12E-23	2.25E-09	0.083	0.012	NLRC5
3	chr3:52827704-52828818	8	7.47E-15	2.31E-09	-0.057	-0.018	ITIH3
17	chr17:7381622-7383488	12	1.42E-14	3.08E-09	-0.052	-0.008	ZBTB4, SLC35G6
4	chr4:527125-530543	11	2.31E-06	4.59E-09	0.077	0.004	PIGG
11	chr11:1000533-1002310	10	4.39E-07	6.92E-09	0.065	0.001	AP2A2
10	chr10:48416391-48417904	13	2.65E-13	7.32E-09	-0.050	-0.014	GDF2
22	chr22:50246573-50249174	9	7.26E-11	8.22E-09	-0.054	-0.008	ZBED4
14	chr14:101402494-101402822	2	3.16E-11	1.32E-08	-0.050	-0.032	SNORD113-4, AL132709.5, SNORD113-5
12	chr12:132269563-132270829	7	5.24E-14	1.60E-08	-0.062	-0.015	SFSWAP
22	chr22:50982818-50988451	16	7.04E-09	1.80E-08	0.072	0.006	KLHDC7B, CTA-384D8.31, CTA-384D8.35, CTA-384D8.34
7	chr7:150099648-150105713	21	5.62E-07	2.34E-08	-0.067	-0.003	ZNF775
11	chr11:1463541-1464792	8	9.27E-11	3.83E-08	-0.054	-0.025	BRSK2
4	chr4:15428750-15429967	6	3.10E-07	3.86E-08	-0.092	-0.019	C1QTNF7, RP11-665G4.1
10	chr10:133961235-133961825	5	6.56E-09	4.75E-08	0.082	0.004	NA
6	chr6:131147216-131148736	3	3.54E-13	5.32E-08	-0.080	-0.043	SMLR1
10	chr10:134793190-134798264	14	1.91E-09	6.56E-08	-0.053	-0.013	NA
8	chr8:144599027-144599347	3	3.08E-10	7.31E-08	-0.076	-0.042	NA
6	chr6:33091111-33093109	8	1.65E-07	9.06E-08	-0.068	-0.017	NA
2	chr2:3704501-3705649	7	4.58E-09	9.23E-08	-0.054	-0.035	ALLC
3	chr3:142314493-142316082	11	2.54E-09	1.06E-07	-0.058	-0.006	PLS1
12	chr12:2943902-2944493	8	1.76E-08	1.09E-07	-0.052	-0.029	NRIP2, ITFG2
6	chr6:31079152-31082631	8	3.24E-10	1.25E-07	-0.198	-0.034	C6orf15, PSORS1C1
6	chr6:31145302-31146942	8	1.11E-14	1.26E-07	-0.062	-0.017	PSORS1C3, POU5F1
19	chr19:5587239-5588795	5	4.60E-09	1.46E-07	-0.085	-0.024	SAFB2-010
1	chr1:2846836-2848427	8	3.49E-08	1.61E-07	-0.072	-0.017	NA
8	chr8:72459499-72460643	7	5.33E-15	1.67E-07	-0.050	-0.014	RP11-1102P16.1
5	chr5:135414858-135416613	19	7.11E-07	1.80E-07	-0.062	-0.038	VTRNA2-1
1	chr1:75590483-75591353	5	9.03E-08	1.97E-07	-0.051	-0.037	RP11-510C10.4
6	chr6:29690766-29693534	29	2.13E-21	2.04E-07	0.076	0.007	HLA-F
18	chr18:55887763-55888832	5	2.13E-09	2.31E-07	-0.051	-0.024	NEDD4L
2	chr2:231989146-231989824	3	2.83E-14	2.34E-07	-0.059	-0.040	HTR2B

17	chr17:5401814-5405260	18	2.98E-06	2.77E-07	0.065	0.009	NLRP1
2	chr2:208027870-208028218	2	1.42E-09	2.77E-07	-0.051	-0.032	NA
17	chr17:33473881-33475031	6	5.31E-15	2.79E-07	-0.065	-0.018	UNC45B
6	chr6:9896436-9897093	4	1.84E-08	3.33E-07	-0.052	-0.016	NA
19	chr19:57741325-57743416	9	2.06E-08	4.82E-07	-0.058	-0.031	AURKC
11	chr11:39689583-39689828	2	1.28E-08	5.17E-07	-0.054	-0.036	NA
2	chr2:242609764-242612684	10	1.13E-06	5.27E-07	-0.063	-0.011	ATG4B-029
11	chr11:65684313-65687892	13	1.06E-06	5.66E-07	-0.073	-0.010	DRAP1, C11orf68
11	chr11:58598239-58598338	2	1.82E-08	5.82E-07	-0.068	-0.064	NA
8	chr8:144651767-144656997	17	7.56E-06	6.59E-07	-0.054	-0.013	MROH6, NAPRT1, MROH6, RP11-661A12.9
7	chr7:4264980-4265548	6	1.77E-07	6.73E-07	-0.052	-0.014	SDK1
12	chr12:41581414-41582362	5	5.96E-16	6.79E-07	-0.067	-0.025	PDZRN4
11	chr11:126581947-126582317	5	1.08E-07	7.68E-07	-0.063	-0.033	NA
20	chr20:61297564-61299190	9	2.23E-07	9.50E-07	-0.057	-0.015	RP11-93B14.5, SLCO4A1
2	chr2:70141289-70143463	13	1.51E-09	9.65E-07	-0.050	-0.003	MXD1, snoU13.84
10	chr10:134923599-134924050	2	6.65E-08	1.07E-06	-0.061	-0.044	NA
6	chr6:76203530-76204308	7	1.41E-09	1.14E-06	-0.055	-0.028	FILIP1
6	chr6:32802598-32807895	48	1.40E-14	1.20E-06	-0.120	-0.004	TAP2, PSMB8
3	chr3:52302150-52303511	5	1.18E-08	1.23E-06	0.053	-0.002	MIRLET7G, WDR82
13	chr13:28562668-28563639	7	3.71E-07	1.27E-06	-0.070	-0.013	URAD, RN7SL272P
2	chr2:239360713-239362030	5	6.06E-07	1.32E-06	0.094	0.016	ASB1-008
21	chr21:36258423-36261702	14	3.83E-06	1.32E-06	0.056	0.023	RUNX1
6	chr6:28874547-28877495	13	5.80E-07	1.35E-06	-0.062	-0.012	TRIM27
16	chr16:3988694-3989432	7	1.91E-09	1.36E-06	-0.056	-0.023	NA
6	chr6:158013621-158014655	5	5.41E-07	1.36E-06	-0.067	-0.026	ZDHHC14
21	chr21:42796537-42799141	10	5.01E-06	1.37E-06	0.055	0.009	MX1
11	chr11:379296-380593	4	6.33E-07	1.64E-06	-0.058	-0.038	B4GALNT4
14	chr14:104625249-104626668	4	3.40E-12	1.93E-06	-0.073	-0.029	NA
3	chr3:37283665-37285071	12	3.16E-07	1.97E-06	-0.058	-0.006	AC097359.1, GOLGA4, RP11-259K5.2
11	chr11:116657918-116664039	24	1.08E-17	2.40E-06	-0.124	-0.011	ZNF259, APOA5
19	chr19:38039469-38040340	3	1.11E-06	2.59E-06	-0.057	-0.033	CTD-3064H18.4, ZNF540, ZNF571-AS1
13	chr13:40107605-40108007	4	1.90E-09	2.64E-06	-0.064	-0.027	NA
13	chr13:113819073-113819409	5	4.35E-07	2.96E-06	-0.068	-0.027	PROZ, RP11-98F14.11
13	chr13:113663206-113664019	4	1.19E-07	3.07E-06	-0.052	-0.016	RP11-120K24.5-001, RP11-120K24.4-001
1	chr1:2943306-2943891	2	1.55E-11	3.13E-06	-0.050	-0.040	NA
17	chr17:39661354-39662980	5	8.48E-07	3.66E-06	0.075	0.003	KRT13
1	chr1:166889789-166890924	7	2.86E-06	3.88E-06	-0.052	-0.020	NA
17	chr17:33842255-33842301	2	2.41E-07	4.28E-06	-0.060	-0.053	RP11-1094M14.4
4	chr4:167020896-167022415	3	8.55E-07	4.70E-06	-0.061	-0.030	NA
8	chr8:1327367-1328459	5	3.04E-06	5.42E-06	0.056	0.000	NA
19	chr19:51020297-51022341	7	4.96E-07	5.46E-06	-0.065	-0.014	NA
20	chr20:23548991-23550632	6	5.52E-06	5.51E-06	-0.114	-0.026	CST9L
17	chr17:34266290-34267111	2	3.39E-07	5.66E-06	-0.053	-0.036	LYZL6
10	chr10:127371542-127373136	5	8.09E-06	5.89E-06	0.121	0.016	TEX36, RP11-383C5.3
3	chr3:192576964-192577951	2	1.11E-07	6.05E-06	-0.068	-0.052	NA
13	chr13:100583767-100583872	3	1.06E-09	6.52E-06	-0.058	-0.030	NA
7	chr7:100881429-100884094	9	3.30E-10	6.58E-06	-0.055	-0.013	CLDN15, FIS1
17	chr17:80708279-80710877	13	9.35E-07	6.58E-06	-0.091	-0.016	TBCD

6	chr6:1101607-1102379	4	9.17E-08	7.77E-06	0.055	0.002	NA
3	chr3:157221198-157222026	4	8.35E-07	8.61E-06	-0.057	-0.018	VEPH1
18	chr18:71982691-71983824	3	2.21E-07	9.21E-06	-0.060	-0.032	C18orf63
12	chr12:132654742-132657879	13	2.07E-07	9.74E-06	-0.062	-0.016	NA
1	chr1:95698378-95700272	10	5.69E-09	1.07E-05	-0.053	-0.015	RWDD3, RP11-57H12.3
14	chr14:103367306-103367858	4	2.30E-06	1.15E-05	-0.051	-0.039	NA
2	chr2:1050113-1051469	4	3.35E-11	1.15E-05	-0.054	-0.021	NA
6	chr6:166571782-166572045	4	6.97E-07	1.16E-05	0.086	0.015	NA
14	chr14:74484925-74486651	9	6.84E-08	1.22E-05	-0.055	-0.006	ENTPD5, CCDC176
20	chr20:30027638-30028893	9	4.14E-06	1.25E-05	-0.050	-0.017	DEFB123
16	chr16:83985744-83986941	7	3.28E-07	1.28E-05	-0.057	-0.010	OSGIN1
19	chr19:15162623-15163470	4	1.47E-07	1.53E-05	-0.058	-0.026	CASP14, AC004699.1
17	chr17:1645410-1646196	4	4.22E-06	1.54E-05	0.084	0.016	SERPINF2
17	chr17:76445798-76446821	3	3.15E-06	1.58E-05	-0.055	-0.027	DNAH17
17	chr17:37321201-37322540	6	1.01E-04	2.06E-05	0.067	0.031	ARL5C
2	chr2:24367779-24367888	3	3.80E-06	2.06E-05	0.120	0.037	FAM228B
12	chr12:124826534-124827729	5	1.71E-05	2.26E-05	-0.078	-0.019	NCOR2
10	chr10:44223934-44224459	3	1.01E-05	2.87E-05	-0.056	-0.037	RP11-272J7.6
10	chr10:2543474-2544596	6	6.03E-05	2.99E-05	-0.055	-0.035	RP11-526P5.1
16	chr16:22175965-22176873	2	9.77E-06	3.11E-05	-0.057	-0.040	SDR42E2
1	chr1:54358862-54360170	7	1.34E-06	3.12E-05	-0.064	-0.018	DIO1
5	chr5:178564818-178565937	6	3.16E-06	3.25E-05	-0.058	-0.019	NA
12	chr12:2017093-2017746	5	7.50E-06	3.49E-05	-0.053	-0.028	NA
8	chr8:120427810-120428692	5	5.80E-07	3.61E-05	-0.053	-0.013	NOV
1	chr1:56453730-56454285	2	6.01E-06	3.71E-05	-0.054	-0.029	NA
12	chr12:11422897-11423120	3	2.64E-06	4.19E-05	-0.072	-0.027	PRB3
7	chr7:158766061-158767135	4	1.92E-05	4.28E-05	-0.063	-0.025	NA
5	chr5:1110019-1112251	11	1.87E-05	4.71E-05	0.055	0.003	SLC12A7-001
4	chr4:139144433-139145210	2	1.33E-06	4.83E-05	-0.054	-0.028	SLC7A11
1	chr1:215178601-215179216	5	1.10E-06	4.95E-05	-0.052	-0.017	KCNK2
16	chr16:8622412-8623289	4	9.08E-09	5.10E-05	-0.056	-0.034	TMEM114
10	chr10:35080181-35081047	3	3.76E-05	5.51E-05	-0.059	-0.034	NA
12	chr12:132293329-132293702	4	4.04E-05	5.83E-05	-0.051	-0.043	NA
11	chr11:68199656-68201194	7	5.14E-08	6.60E-05	-0.057	-0.014	NA
16	chr16:89219415-89220554	4	1.05E-05	7.38E-05	-0.052	-0.019	NA
9	chr9:130955135-130956057	4	6.75E-05	7.64E-05	-0.055	-0.041	CIZ1
2	chr2:85389840-85390041	3	5.53E-07	8.06E-05	-0.050	-0.022	NA
1	chr1:212002970-212004468	11	2.37E-05	9.08E-05	0.062	0.005	LPGAT1, RP11-552D8.1
1	chr1:203051472-203052423	2	2.77E-04	1.03E-04	-0.051	-0.036	NA
1	chr1:3102750-3105326	8	1.70E-04	1.06E-04	-0.061	-0.023	NA
17	chr17:78336395-78338052	7	5.31E-05	1.28E-04	-0.056	-0.012	RNF213
3	chr3:9744891-9746059	9	9.35E-07	1.29E-04	-0.104	-0.016	CPNE9
10	chr10:85985222-85986738	8	6.30E-07	1.30E-04	0.096	0.002	LRIT2
6	chr6:170531180-170532835	11	3.78E-06	1.45E-04	0.051	0.009	NA
8	chr8:142263798-142264816	6	6.46E-05	1.54E-04	0.082	0.010	SLC45A4, RP11-10J21.4
10	chr10:31107995-31108101	2	1.04E-05	1.56E-04	-0.064	-0.043	NA
17	chr17:39539075-39539863	2	7.25E-05	1.64E-04	-0.061	-0.036	KRT34
5	chr5:42953543-42953590	2	2.02E-05	1.99E-04	-0.056	-0.053	NA

19	chr19:10225927-10226701	5	7.16E-07	2.09E-04	-0.059	-0.018	EIF3G
2	chr2:242004954-242005101	2	5.22E-05	2.10E-04	-0.053	-0.030	AC005237.4-001
8	chr8:143645661-143645857	3	1.67E-06	2.15E-04	0.051	0.004	NA
3	chr3:46872239-46872949	2	1.66E-04	2.26E-04	-0.076	-0.058	NA
11	chr11:132912205-132912368	3	1.09E-04	2.28E-04	-0.080	-0.036	NA
9	chr9:74061171-74062096	5	2.87E-05	2.28E-04	-0.052	-0.022	TRPM3
7	chr7:112725899-112727914	14	6.66E-12	2.29E-04	-0.060	-0.014	GPR85
7	chr7:64034943-64035529	2	6.14E-05	2.39E-04	-0.057	-0.048	NA
14	chr14:68161980-68163792	7	9.97E-07	2.50E-04	0.060	0.008	RDH11
7	chr7:157193063-157193615	3	3.67E-05	2.51E-04	-0.052	-0.020	NA
2	chr2:118616155-118616576	4	1.48E-04	2.52E-04	-0.057	-0.038	HTR5BP
15	chr15:101084509-101085710	7	3.68E-04	2.53E-04	0.053	0.027	CERS3
10	chr10:133208486-133208984	5	1.47E-07	2.57E-04	-0.067	-0.017	NA
11	chr11:132951838-132951950	3	3.80E-05	2.76E-04	-0.058	-0.037	NA
1	chr1:203273149-203276595	13	4.52E-05	2.87E-04	-0.053	-0.006	BTG2, LINC01136
11	chr11:69462783-69464272	6	4.60E-07	3.12E-04	0.069	0.011	CCND1
16	chr16:14379220-14380714	5	2.01E-04	4.17E-04	0.107	0.023	NA
16	chr16:72459516-72460114	7	9.62E-05	4.26E-04	0.059	0.004	AC004158.3
13	chr13:41034925-41035061	2	2.54E-06	4.32E-04	-0.065	-0.033	LINC00598
8	chr8:2002012-2002553	2	3.12E-06	4.41E-04	-0.080	-0.049	NA
15	chr15:29423177-29424512	4	8.08E-05	4.50E-04	-0.050	-0.021	NA
5	chr5:78279841-78283014	12	4.82E-05	4.82E-04	0.063	0.006	ARSB
16	chr16:85122558-85124401	4	4.45E-06	4.83E-04	-0.085	-0.029	NA
2	chr2:200468445-200469026	5	4.89E-04	4.94E-04	-0.052	-0.048	NA
10	chr10:134910285-134912411	9	3.26E-04	5.66E-04	0.058	0.004	NA
15	chr15:25123287-25124213	3	8.33E-05	5.68E-04	-0.054	-0.035	NA
8	chr8:599963-600940	7	1.07E-03	6.13E-04	-0.050	-0.037	NA
8	chr8:142369468-142369705	2	5.10E-05	6.31E-04	-0.060	-0.045	NA
7	chr7:150497065-150499362	12	3.67E-06	6.36E-04	-0.056	-0.010	TMEM176A, TMEM176B
14	chr14:74703888-74704714	3	1.01E-04	6.59E-04	-0.074	-0.036	VSX2
11	chr11:71237522-71238205	2	5.31E-07	6.61E-04	0.052	0.015	KRTAP5-7
9	chr9:136397058-136397740	2	2.19E-04	7.59E-04	-0.102	-0.068	ADAMTSL2
16	chr16:83171155-83171992	3	3.65E-04	7.86E-04	-0.051	-0.038	NA
8	chr8:140926709-140927326	3	2.86E-05	8.47E-04	0.064	0.015	RP11-284H18.1
13	chr13:86373320-86373600	2	2.89E-05	8.54E-04	-0.050	-0.032	SLITRK6
8	chr8:145637525-145639181	8	1.41E-05	9.37E-04	-0.092	-0.039	SLC39A4
7	chr7:27450882-27451047	2	3.38E-04	9.90E-04	-0.107	-0.062	NA
7	chr7:1209495-1209742	3	5.98E-04	1.03E-03	-0.073	-0.050	NA
2	chr2:98858011-98858595	4	4.71E-05	1.12E-03	-0.053	-0.019	NA
8	chr8:144301594-144303509	5	1.19E-04	1.32E-03	-0.061	-0.029	NA
7	chr7:157986877-157987184	4	2.54E-04	1.39E-03	-0.057	-0.018	NA
5	chr5:170845058-170847313	11	8.78E-06	1.43E-03	-0.059	-0.006	FGF18
2	chr2:114035983-114037060	5	8.61E-05	1.56E-03	-0.098	-0.026	PAX8
5	chr5:16178784-16181039	18	3.46E-08	1.57E-03	-0.059	-0.007	MARCH11, RP11-19O2.2
3	chr3:109115356-109115491	3	1.14E-03	1.58E-03	-0.072	-0.030	RP11-702L6.1
7	chr7:108524200-108524793	2	8.54E-04	1.60E-03	-0.060	-0.032	C7orf66
20	chr20:58513509-58515891	12	8.08E-06	1.65E-03	-0.051	-0.007	FAM217B, PPP1R3D
14	chr14:94491763-94493296	8	1.62E-05	1.67E-03	-0.053	-0.009	OTUB2

12	chr12:4829115-4829827	6	9.83E-05	1.71E-03	-0.052	-0.020	GALNT8, RP11-234B24.2
5	chr5:180581301-180582587	7	3.59E-05	1.92E-03	0.060	0.003	OR2V2-001
10	chr10:134723491-134725087	5	2.97E-04	1.96E-03	-0.059	-0.019	TTC40-005
12	chr12:132290859-132290929	2	1.03E-03	2.04E-03	-0.050	-0.027	NA
16	chr16:1019956-1022650	13	8.79E-07	2.06E-03	0.098	0.007	LMF1
10	chr10:131669406-131669630	3	1.63E-03	2.17E-03	-0.060	-0.046	NA
12	chr12:127226034-127226110	2	4.65E-06	2.20E-03	-0.059	-0.042	LINC00943
21	chr21:46810558-46811242	2	5.78E-04	2.21E-03	-0.058	-0.031	NA
4	chr4:189139691-189140097	2	1.12E-03	2.28E-03	-0.060	-0.032	NA
1	chr1:205054684-205054828	2	1.11E-03	2.32E-03	-0.051	-0.028	TMEM81
12	chr12:41830066-41830372	2	9.39E-05	2.47E-03	-0.095	-0.060	PDZRN4
1	chr1:171282607-171283893	4	4.04E-04	2.71E-03	-0.065	-0.017	FMO4
3	chr3:10005747-10005827	2	1.31E-03	2.74E-03	-0.086	-0.045	NA
5	chr5:167830729-167831317	2	2.46E-04	2.86E-03	-0.064	-0.037	WWC1
9	chr9:140707378-140709558	4	7.89E-05	2.89E-03	-0.054	-0.015	EHMT1
17	chr17:17109570-17110353	10	1.44E-09	2.99E-03	-0.058	-0.026	PLD6
22	chr22:32600722-32601654	5	3.49E-03	3.20E-03	-0.064	-0.027	RFPL2, RP1-90G24.10
17	chr17:76449477-76449599	3	6.29E-04	3.36E-03	-0.051	-0.018	NA
10	chr10:134726665-134726771	2	1.95E-03	3.60E-03	-0.107	-0.092	NA
1	chr1:45285821-45286062	3	3.20E-03	3.89E-03	-0.050	-0.043	RNU5E-6P
1	chr1:217113226-217114101	2	1.22E-04	4.18E-03	-0.054	-0.029	ESRRG
16	chr16:83967808-83968911	3	3.01E-05	4.31E-03	0.051	0.010	NA
11	chr11:12620869-12621248	2	5.01E-04	4.39E-03	-0.057	-0.030	NA
4	chr4:33062752-33062845	2	2.38E-03	4.76E-03	-0.100	-0.054	NA
1	chr1:3323806-3324671	3	2.30E-05	4.80E-03	-0.069	-0.025	NA
3	chr3:155421970-155423168	5	1.68E-03	4.81E-03	-0.078	-0.022	PLCH1
3	chr3:2552181-2553187	4	9.24E-03	4.84E-03	0.072	0.007	CNTN4
4	chr4:155312410-155313256	3	3.55E-05	4.89E-03	0.066	0.014	DCHS2
14	chr14:68167893-68168587	2	4.16E-03	5.31E-03	-0.051	-0.032	RDH12
12	chr12:131515293-131517216	4	1.80E-10	5.45E-03	-0.050	-0.020	AC078925.1
11	chr11:3168455-3169027	3	1.09E-03	5.83E-03	-0.056	-0.025	NA
13	chr13:114161463-114161939	2	4.35E-04	5.84E-03	0.084	0.007	NA
9	chr9:139606200-139608277	7	2.67E-04	6.46E-03	-0.083	-0.013	FAM69B
16	chr16:88757588-88757816	3	1.66E-04	6.63E-03	-0.076	-0.021	NA
19	chr19:843541-844589	4	1.70E-04	6.82E-03	-0.056	-0.023	PRTN3
17	chr17:58165865-58166296	3	5.91E-03	6.86E-03	-0.080	-0.031	CTD-2319I12.1
17	chr17:80849289-80849463	3	2.27E-03	7.04E-03	-0.105	-0.039	TBCD
8	chr8:142283564-142283615	2	1.99E-03	7.30E-03	0.054	0.026	NA
11	chr11:50257256-50258750	6	3.70E-04	7.34E-03	-0.052	-0.020	RP11-347H15.4
7	chr7:154958558-154958976	4	7.09E-06	7.88E-03	0.053	0.006	NA
4	chr4:753903-754733	2	4.10E-04	8.83E-03	-0.055	-0.028	NA
7	chr7:75372083-75372874	2	3.90E-03	8.98E-03	-0.101	-0.052	NA
13	chr13:31734946-31736727	15	3.23E-04	9.08E-03	-0.051	-0.003	HSPH1
20	chr20:6034446-6035902	6	5.14E-04	9.31E-03	-0.083	-0.020	LRRN4
7	chr7:158250911-158251297	3	5.23E-03	9.46E-03	-0.054	-0.032	NA
14	chr14:77498304-77499911	9	5.69E-05	9.77E-03	-0.105	-0.012	NA
13	chr13:111464554-111466326	4	2.42E-03	9.99E-03	-0.062	-0.028	LINC00567-001
7	chr7:157289477-157289496	2	7.40E-03	1.09E-02	-0.058	-0.030	NA

3	chr3:108475765-108476878	5	4.88E-06	1.10E-02	-0.052	-0.022	RETNLB
1	chr1:156616554-156617074	6	5.01E-03	1.13E-02	0.055	0.037	BCAN, RP11-284F21.10
13	chr13:111969980-111970282	3	2.70E-04	1.25E-02	-0.091	-0.031	TEX29-001
13	chr13:110319562-110319607	3	1.43E-02	1.31E-02	-0.055	-0.043	NA
7	chr7:116199599-116200522	3	2.39E-03	1.33E-02	0.070	0.015	NA
17	chr17:78039368-78039437	2	9.34E-03	1.37E-02	-0.066	-0.038	NA
7	chr7:151134527-151135375	2	4.35E-03	1.42E-02	0.065	0.031	CRYGN
19	chr19:18959208-18960246	4	1.55E-03	1.45E-02	-0.080	-0.026	UPF1
17	chr17:78150934-78152466	8	7.66E-04	1.67E-02	0.056	0.001	CARD14
6	chr6:31379258-31380248	4	2.12E-02	1.79E-02	0.100	0.022	NA
14	chr14:97431088-97431367	4	1.40E-02	1.98E-02	-0.085	-0.023	NA
7	chr7:91795363-91795403	2	3.32E-03	2.02E-02	0.053	0.023	LRRD1
22	chr22:43739790-43741137	5	9.63E-03	2.11E-02	-0.054	-0.021	SCUBE1
11	chr11:71229988-71230303	2	1.69E-02	2.13E-02	0.096	0.043	NA
6	chr6:168435636-168436353	4	1.24E-02	2.16E-02	-0.054	-0.033	KIF25
4	chr4:7301176-7301854	6	6.80E-03	2.25E-02	-0.051	-0.020	NA
7	chr7:6653510-6655834	6	4.04E-04	2.32E-02	0.115	0.017	ZNF853
12	chr12:55368244-55369150	2	5.81E-04	2.45E-02	0.069	0.033	TESPA1
17	chr17:3833739-3833873	3	3.52E-03	2.60E-02	-0.061	-0.024	ATP2A3
20	chr20:55664666-55665224	2	2.52E-02	2.62E-02	-0.079	-0.047	NA
17	chr17:211482-212481	2	2.52E-02	2.95E-02	-0.057	-0.034	NA
14	chr14:69725831-69727352	13	2.38E-04	3.05E-02	-0.051	-0.005	GALNT16, RP11-363J20.2
1	chr1:43473840-43473978	2	2.67E-02	3.05E-02	0.073	0.035	NA
19	chr19:51829984-51831274	6	1.43E-03	3.07E-02	-0.050	-0.011	NA
10	chr10:601783-601816	2	2.80E-02	3.26E-02	-0.077	-0.069	NA
1	chr1:227505845-227506868	8	3.59E-04	3.65E-02	-0.058	-0.007	CDC42BPA
4	chr4:16077324-16077853	4	2.32E-02	3.70E-02	-0.063	-0.019	PROM1
6	chr6:114181686-114181798	2	2.29E-02	3.97E-02	0.054	0.039	NA
8	chr8:582908-583068	2	1.67E-02	4.01E-02	-0.057	-0.036	NA
8	chr8:1821338-1822568	6	3.01E-06	4.10E-02	-0.069	-0.013	ARHGEF10
12	chr12:1675846-1676077	3	1.98E-02	4.25E-02	-0.054	-0.032	NA
19	chr19:17905332-17906309	6	1.18E-03	4.39E-02	-0.057	-0.021	B3GNT3
18	chr18:47015710-47016218	3	2.57E-02	4.41E-02	0.118	0.038	RPL17-C18orf32, SNORD58C, SNORD58A, MIR1539, RPL17, SNORD58B
10	chr10:79273606-79273865	2	3.71E-02	4.43E-02	-0.053	-0.029	NA
9	chr9:86535572-86536513	3	4.58E-02	4.81E-02	-0.054	-0.019	KIF27

Table S3. Results from gene-set enrichment analysis of biological processes performed by DAVID (95 significant biological processes for 862 unique genes for 1,309 selected CpG sites)

Term	Genes involved	% (Involved Genes / Total Genes)	P-Value	Benjamini
regulation of multicellular organismal process	170	20.6	2.40E-08	1.50E-04
negative regulation of biological process	263	31.9	4.80E-08	1.50E-04
negative regulation of cellular process	247	30	7.40E-08	1.60E-04
cell adhesion	118	14.3	1.00E-07	1.60E-04
biological adhesion	118	14.3	1.20E-07	1.50E-04
cell-cell adhesion	88	10.7	1.90E-07	1.90E-04
regulation of cellular component organization	147	17.8	5.50E-07	5.00E-04
single-multicellular organism process	316	38.3	8.70E-07	6.80E-04
multicellular organism development	267	32.4	1.20E-06	8.40E-04
system development	240	29.1	1.20E-06	7.60E-04
regulation of cell projection organization	51	6.2	1.30E-06	7.20E-04
regulation of cell differentiation	105	12.7	1.70E-06	8.80E-04
regulation of developmental process	135	16.4	1.80E-06	8.60E-04
positive regulation of cellular process	259	31.4	2.60E-06	1.10E-03
regulation of multicellular organismal development	113	13.7	3.20E-06	1.40E-03
positive regulation of biological process	283	34.3	3.30E-06	1.30E-03
positive regulation of RNA metabolic process	96	11.7	5.70E-06	2.10E-03
regulation of signaling	178	21.6	7.30E-06	2.60E-03
positive regulation of nucleic acid-templated transcription	92	11.2	7.60E-06	2.50E-03
positive regulation of transcription, DNA-templated	92	11.2	7.60E-06	2.50E-03
positive regulation of developmental process	78	9.5	8.00E-06	2.50E-03
cell development	123	14.9	1.20E-05	3.60E-03
regulation of transcription from RNA polymerase II promoter	116	14.1	1.30E-05	3.80E-03
single organism cell adhesion	60	7.3	1.30E-05	3.70E-03
positive regulation of RNA biosynthetic process	92	11.2	1.40E-05	3.60E-03
regulation of neuron projection development	38	4.6	1.80E-05	4.50E-03
regulation of cell communication	173	21	2.20E-05	5.30E-03
developmental process	293	35.6	2.40E-05	5.50E-03
negative regulation of protein kinase activity	26	3.2	3.00E-05	6.60E-03
single-organism developmental process	285	34.6	3.30E-05	7.10E-03
regulation of small GTPase mediated signal transduction	30	3.6	3.40E-05	7.20E-03
anatomical structure development	284	34.5	4.40E-05	8.90E-03
nervous system development	130	15.8	4.70E-05	9.10E-03
Ras protein signal transduction	31	3.8	4.80E-05	9.10E-03
regulation of cell development	62	7.5	4.90E-05	9.00E-03

positive regulation of gene expression	106	12.9	5.10E-05	9.10E-03
cell-cell signaling	97	11.8	5.20E-05	9.10E-03
neuron projection development	60	7.3	5.30E-05	8.90E-03
positive regulation of macromolecule biosynthetic process	101	12.3	5.60E-05	9.20E-03
positive regulation of cell differentiation	61	7.4	6.50E-05	1.00E-02
negative regulation of phosphorylation	37	4.5	8.60E-05	1.30E-02
positive regulation of GTPase activity	49	5.9	8.80E-05	1.30E-02
negative regulation of macromolecule metabolic process	136	16.5	9.20E-05	1.40E-02
negative regulation of kinase activity	26	3.2	9.30E-05	1.30E-02
positive regulation of nucleobase-containing compound metabolic process	103	12.5	9.40E-05	1.30E-02
regulation of GTPase activity	52	6.3	1.00E-04	1.40E-02
positive regulation of transcription from RNA polymerase II promoter	71	8.6	1.10E-04	1.40E-02
positive regulation of nitrogen compound metabolic process	108	13.1	1.10E-04	1.50E-02
positive regulation of multicellular organismal process	91	11	1.20E-04	1.50E-02
positive regulation of cellular biosynthetic process	106	12.9	1.20E-04	1.50E-02
regulation of nervous system development	56	6.8	1.20E-04	1.50E-02
regulation of cell growth	35	4.2	1.30E-04	1.60E-02
behavior	44	5.3	1.30E-04	1.60E-02
regulation of response to stimulus	197	23.9	1.40E-04	1.60E-02
synapse organization	24	2.9	1.50E-04	1.70E-02
regulation of neuron differentiation	44	5.3	1.50E-04	1.70E-02
leukocyte differentiation	39	4.7	1.50E-04	1.70E-02
regulation of Ras protein signal transduction	22	2.7	1.70E-04	1.80E-02
neurogenesis	91	11	1.70E-04	1.80E-02
single organismal cell-cell adhesion	53	6.4	1.80E-04	1.90E-02
cell differentiation	197	23.9	1.80E-04	1.90E-02
growth	64	7.8	2.00E-04	2.00E-02
regulation of localization	141	17.1	2.10E-04	2.10E-02
generation of neurons	86	10.4	2.10E-04	2.10E-02
lamellipodium organization	12	1.5	2.20E-04	2.20E-02
peptidyl-threonine modification	13	1.6	2.30E-04	2.20E-02
positive regulation of biosynthetic process	106	12.9	2.30E-04	2.20E-02
regulation of neurogenesis	50	6.1	2.60E-04	2.40E-02
neuron development	65	7.9	3.00E-04	2.70E-02
transcription from RNA polymerase II promoter	108	13.1	3.50E-04	3.10E-02
positive regulation of cellular component organization	76	9.2	3.50E-04	3.10E-02
cell projection organization	82	10	3.70E-04	3.20E-02
negative regulation of protein phosphorylation	33	4	3.70E-04	3.20E-02
regulation of cell morphogenesis	43	5.2	3.90E-04	3.30E-02
T cell aggregation	36	4.4	4.10E-04	3.40E-02

T cell activation	36	4.4	4.10E-04	3.40E-02
lymphocyte aggregation	36	4.4	4.20E-04	3.40E-02
cell morphogenesis involved in differentiation	53	6.4	4.20E-04	3.40E-02
negative regulation of multicellular organismal process	67	8.1	4.40E-04	3.60E-02
locomotion	93	11.3	4.50E-04	3.60E-02
regulation of signal transduction	151	18.3	4.60E-04	3.60E-02
regulation of phagocytosis	11	1.3	5.20E-04	4.00E-02
multicellular organismal process	344	41.7	5.40E-04	4.10E-02
regulation of synapse structure or activity	23	2.8	5.40E-04	4.10E-02
cell growth	35	4.2	5.50E-04	4.00E-02
leukocyte aggregation	36	4.4	5.60E-04	4.10E-02
cell migration	75	9.1	5.60E-04	4.10E-02
negative regulation of cell communication	75	9.1	5.70E-04	4.10E-02
peptidyl-threonine phosphorylation	12	1.5	5.70E-04	4.00E-02
negative regulation of signaling	75	9.1	6.00E-04	4.20E-02
positive regulation of cell projection organization	27	3.3	6.20E-04	4.30E-02
leukocyte activation	52	6.3	6.50E-04	4.50E-02
developmental growth	43	5.2	6.70E-04	4.50E-02
regulation of protein kinase activity	51	6.2	6.70E-04	4.50E-02
regulation of locomotion	53	6.4	6.80E-04	4.50E-02

Table S4. CpG sites (N=60) out of the selected CpG sites for the combined group (N=1,309) that were also selected in stratified analyses for both boys and girls, sorted by P-value from smallest to largest. Also presented is the rank of each CpG site by P-value in each relevant analysis

CpG	CHR	Position	Gene symbol	Relation to gene	Relation to Island	Beta (HIV+)	Beta (HIV-)	Mean Diff	P	Adj P	All Rank	Boys Rank	Girls Rank
cg22690339	6	38249061	BTBD9	Body	OpenSea	0.797	0.740	0.057	3.29E-08	1.26E-04	10	957	5
cg08237220	3	156332967			OpenSea	0.536	0.464	0.072	2.28E-07	3.54E-04	13	716	10
cg18394552	5	159428643			OpenSea	0.737	0.661	0.076	8.50E-07	6.68E-04	16	472	17
cg07839457	16	57023022	NLRC5	TSS1500	N_Shore	0.254	0.343	-0.089	4.31E-06	1.64E-03	3	17	29
cg15773890	6	17259549			OpenSea	0.869	0.813	0.056	5.21E-06	1.78E-03	34	794	32
cg12027254	17	76055290	TNRC6C	Body	OpenSea	0.821	0.768	0.053	5.63E-06	1.83E-03	4	27	36
cg14418857	7	121120431			OpenSea	0.610	0.546	0.063	8.42E-06	2.24E-03	20	171	41
cg22647738	17	34304462	CCL16	3'UTR	OpenSea	0.638	0.570	0.068	1.16E-05	2.67E-03	21	221	48
cg09722609	5	149887422	NDST1	TSS1500	OpenSea	0.608	0.549	0.059	1.62E-05	3.24E-03	48	894	55
cg00399027	16	85676861	KIAA0182	5'UTR;Body	S_Shore	0.608	0.553	0.055	2.07E-05	3.68E-03	41	349	60
cg04339360	13	73635568	KLF5	Body	S_Shore	0.704	0.649	0.055	2.27E-05	3.86E-03	49	440	66
cg13561554	15	78795944			N_Shelf	0.627	0.577	0.050	2.63E-05	4.15E-03	66	946	73
cg25879365	1	40510058	CAP1	5'UTR	S_Shelf	0.590	0.519	0.072	3.38E-05	4.72E-03	84	913	80
cg04961225	12	91332479			OpenSea	0.576	0.519	0.057	3.86E-05	5.10E-03	25	192	87
cg20426710	22	50248907	ZBED4	5'UTR	N_Shore	0.515	0.457	0.058	4.26E-05	5.36E-03	50	245	90
cg00105306	15	85194978	WDR73	Body	N_Shelf	0.623	0.569	0.054	4.60E-05	5.58E-03	45	344	91
cg19624420	14	48541885			OpenSea	0.877	0.813	0.063	5.20E-05	5.94E-03	78	478	100
cg04223006	5	82845599	VCAN	Body	OpenSea	0.437	0.363	0.074	6.23E-05	6.56E-03	80	941	107
cg05512157	12	50901878	DIP2B	Body	S_Shelf	0.583	0.532	0.051	6.68E-05	6.83E-03	22	185	113
cg13707760	9	95986383	WNK2	Body	OpenSea	0.511	0.458	0.053	7.35E-05	7.13E-03	15	90	119
cg00787180	14	91751731	CCDC88C	Body	N_Shelf	0.389	0.337	0.052	9.98E-05	8.27E-03	75	907	137
cg10264529	14	24562064	PCK2	TSS1500	N_Shore	0.633	0.582	0.051	1.31E-04	9.58E-03	112	927	154
cg24499605	1	3142925	PRDM16	Body	OpenSea	0.556	0.505	0.052	1.31E-04	9.58E-03	46	329	155
cg01282174	11	119630144			OpenSea	0.492	0.423	0.069	1.38E-04	9.81E-03	158	1059	158
cg00390724	3	18484742			N_Shore	0.508	0.456	0.052	1.42E-04	9.94E-03	9	10	159
cg14799457	18	19927125			N_Shore	0.315	0.263	0.052	1.48E-04	1.02E-02	120	797	162
cg26475911	17	73056187	KCTD2	Body	OpenSea	0.455	0.401	0.054	1.87E-04	1.16E-02	65	308	180
cg27579313	17	7381622	ZBTB4	5'UTR;5'UTR	N_Shore	0.556	0.484	0.072	2.04E-04	1.21E-02	82	453	187
cg13518079	20	2675072	EBF4	Body	S_Shore	0.344	0.263	0.081	2.13E-04	1.24E-02	87	142	191
cg22497969	1	3143018	PRDM16	Body	OpenSea	0.799	0.746	0.052	2.61E-04	1.38E-02	116	688	201
cg14659930	3	114128301	ZBTB20	5'UTR	OpenSea	0.689	0.630	0.060	2.77E-04	1.42E-02	76	421	206
cg17781958	17	79428404	BAHCC1	Body	Island	0.760	0.699	0.060	3.14E-04	1.51E-02	252	1002	218
cg23720384	21	23747055			OpenSea	0.650	0.578	0.073	3.42E-04	1.58E-02	24	59	228
cg25670076	6	90807675	BACH2	5'UTR	OpenSea	0.761	0.707	0.055	3.59E-04	1.62E-02	92	119	232
cg18835596	6	135540107	MYB	3'UTR;	OpenSea	0.723	0.671	0.051	3.78E-04	1.66E-02	43	187	235
cg00602295	16	81328089			OpenSea	0.432	0.381	0.052	3.92E-04	1.70E-02	36	56	241
cg24786658	7	112727423	GPR85	TSS1500;5'UTR	OpenSea	0.698	0.642	0.056	4.56E-04	1.84E-02	251	1010	254
cg24263062	20	2730191	EBF4	Body	Island	0.637	0.565	0.072	4.60E-04	1.85E-02	68	68	255
cg17301248	12	112467203	NAA25	3'UTR	OpenSea	0.636	0.575	0.061	6.75E-04	2.26E-02	162	709	292
cg16321846	7	4050114	SDK1	Body	OpenSea	0.752	0.694	0.059	7.61E-04	2.40E-02	136	376	306
cg26955132	12	13181499			OpenSea	0.740	0.680	0.059	8.05E-04	2.47E-02	132	620	318
cg18808261	3	18464935	SATB1	5'UTR	N_Shore	0.293	0.240	0.053	8.64E-04	2.56E-02	201	802	325

cg05825244	20	2730488	EBF4	Body	Island	0.416	0.289	0.127	9.59E-04	2.70E-02	60	42	337
cg15034267	22	39106452	GTPBP1	Body	S_Shelf	0.767	0.711	0.057	1.03E-03	2.80E-02	169	745	346
cg13485718	14	93315488			OpenSea	0.557	0.507	0.050	1.03E-03	2.80E-02	142	656	347
cg03680873	3	148844300			N_Shelf	0.600	0.544	0.056	1.09E-03	2.89E-02	255	1042	359
cg01232748	4	54928819	CHIC2	Body	N_Shore	0.501	0.449	0.052	1.12E-03	2.92E-02	101	198	360
cg10196532	13	50134640	RCBTB1	Body	OpenSea	0.821	0.742	0.079	1.18E-03	3.00E-02	219	754	368
cg06040034	8	1618448	DLGAP2	Body	S_Shore	0.783	0.730	0.053	1.36E-03	3.22E-02	85	47	396
cg14819618	8	8180214	PRAGMIN	Body	S_Shelf	0.795	0.742	0.053	1.51E-03	3.39E-02	242	982	417
cg15847685	4	62129666			OpenSea	0.791	0.740	0.051	1.52E-03	3.40E-02	118	504	418
cg05739816	6	135989395	C6orf217	Body	OpenSea	0.522	0.467	0.055	1.59E-03	3.47E-02	240	868	422
cg08602008	19	48076841			Island	0.337	0.287	0.050	1.61E-03	3.49E-02	204	748	423
cg08739755	2	172815385	HAT1;HAT1	Body;Body	OpenSea	0.799	0.740	0.059	1.87E-03	3.76E-02	295	633	448
cg07264238	17	33474376	UNC45B	TSS1500	OpenSea	0.775	0.725	0.050	1.99E-03	3.87E-02	51	88	455
cg05857996	20	2675418	EBF4	Body	S_Shore	0.789	0.701	0.088	2.21E-03	4.08E-02	192	305	474
cg00084338	6	170595920	DLL1	Body	N_Shore	0.566	0.466	0.100	2.52E-03	4.36E-02	272	553	496
cg24392970	13	86373600	SLITRK6	TSS200	OpenSea	0.534	0.481	0.053	2.78E-03	4.56E-02	259	1014	513
cg24530147	3	138763894	PRR23C	TSS200	Island	0.795	0.737	0.058	3.07E-03	4.80E-02	322	658	534
cg18640098	5	9888660	LOC285692	Body	OpenSea	0.864	0.811	0.053	3.23E-03	4.91E-02	93	22	546

Table S5. Genes in sex-stratified analysis with 3 or more adjacent differentially methylated CpG sites

**Sex**	**Gene**	**Number of adjacent CpG sites**	**Chromosome**
Boys	COLEC11	6	2
Boys	ALLC	4	2
Boys	PCDHGA2	4	5
Boys	PCDHGB4	4	5
Boys	ADARB2	3	10
Boys	WT1	4	11
Boys	LOC254559	3	15
Boys	BRCA1	3	17
Boys	NBR2	3	17
Girls	B3GNT7	3	2
Girls	C7orf50	3	7
Girls	GPR146	3	7
Girls	IGSF9B	3	11
Girls	CCDC144NL	4	17
Girls	CDH20	4	18

Table S6. CpG sites (N=390) out of the selected 1,309 CpG sites for the primary analysis of all HIV-infected children compared to HIV-uninfected children that were also selected in stratified analyses for both HIV-infected children who started ART early and HIV-infected children who started ART late (both compared to HIV-uninfected children), sorted by P-value from smallest to largest. Also presented is the rank of each CpG site by P-value in each relevant analysis

CpG	CHR	Position	Gene	Relation to Gene	Relation to Island	HIVpos	HIVneg	Beta	P	Adj P	All Rank	EarlyNeg Rank	LateNeg Rank
cg09239591	2	31638511	XDH	TSS1500	OpenSea	0.675	0.623	0.053	2.44E-15	1.97E-11	1	1	1
cg23101680	13	46277286	SPERT	Body	OpenSea	0.717	0.663	0.053	4.89E-14	1.83E-10	2	7	3
cg07839457	16	57023022	NLRC5	TSS1500	N_Shore	0.261	0.353	-0.092	4.69E-11	2.87E-08	3	23	2
cg12027254	17	76055290	TNRC6C	Body	OpenSea	0.824	0.766	0.058	1.00E-10	4.99E-08	4	18	7
cg07970325	6	106497542			OpenSea	0.569	0.512	0.057	1.39E-10	6.38E-08	5	14	14
cg26280998	6	53589544			OpenSea	0.715	0.659	0.056	1.72E-10	7.29E-08	6	37	5
cg00390724	3	18484742			N_Shore	0.519	0.456	0.063	2.66E-10	1.01E-07	9	20	22
cg22690339	6	38249061	BTBD9	Body	OpenSea	0.792	0.735	0.058	3.47E-10	1.22E-07	10	173	4
cg05929755	1	110663559			OpenSea	0.398	0.339	0.059	7.36E-10	2.15E-07	11	174	9
cg12021671	15	42423807			OpenSea	0.744	0.690	0.054	1.02E-09	2.75E-07	12	35	19
cg08237220	3	156332967			OpenSea	0.531	0.459	0.072	1.07E-09	2.85E-07	13	15	36
cg05006384	14	95942319	C14orf49	TSS200	OpenSea	0.648	0.595	0.053	1.74E-09	4.09E-07	14	27	24
cg13707760	9	95986383	WNK2	Body	OpenSea	0.509	0.449	0.060	2.03E-09	4.58E-07	15	109	8
cg18394552	5	159428643			OpenSea	0.732	0.650	0.081	2.42E-09	5.17E-07	16	50	20
cg10336193	1	206848389			OpenSea	0.644	0.584	0.060	2.88E-09	5.88E-07	17	24	39
cg22348290	8	72459499			N_Shore	0.767	0.709	0.058	3.22E-09	6.37E-07	18	54	17
cg26898099	5	95192949	C5orf27	Body	OpenSea	0.706	0.650	0.055	6.44E-09	1.07E-06	19	112	15
cg14418857	7	121120431			OpenSea	0.616	0.535	0.080	6.86E-09	1.13E-06	20	13	109
cg22647738	17	34304462	CCL16	3'UTR	OpenSea	0.639	0.561	0.078	8.18E-09	1.29E-06	21	80	31
cg23720384	21	23747055			OpenSea	0.665	0.583	0.082	1.10E-08	1.61E-06	24	139	29
cg09083958	12	131516365	GPR133	Body	N_Shore	0.696	0.642	0.054	1.53E-08	2.04E-06	28	93	40
cg02341197	21	34185927	C21orf62	5'UTR;1stExon	OpenSea	0.466	0.408	0.058	1.54E-08	2.05E-06	29	16	123
cg08937102	1	1690389	NADK	Body	N_Shore	0.481	0.428	0.053	1.75E-08	2.24E-06	30	56	66
cg16426670	20	2675996	EBF4	Body	S_Shore	0.812	0.750	0.062	2.29E-08	2.74E-06	33	90	52
cg15773890	6	17259549			OpenSea	0.866	0.812	0.054	2.73E-08	3.12E-06	34	141	51
cg00602295	16	81328089			OpenSea	0.441	0.375	0.065	2.81E-08	3.18E-06	36	134	30
cg23429698	8	98878169			N_Shelf	0.324	0.268	0.056	3.13E-08	3.44E-06	39	59	72
cg00399027	16	85676861	KIAA0182	5'UTR;Body	S_Shore	0.607	0.547	0.060	3.72E-08	3.89E-06	41	44	113
cg18835596	6	135540107	MYB	3'UTR	OpenSea	0.725	0.659	0.066	4.46E-08	4.45E-06	43	153	26
cg03182584	7	36364854	KIAA0895	3'UTR	OpenSea	0.689	0.631	0.058	4.53E-08	4.49E-06	44	307	18
cg00105306	15	85194978	WDR73	Body	N_Shelf	0.626	0.569	0.057	4.88E-08	4.75E-06	45	114	90
cg24499605	1	3142925	PRDM16	Body	OpenSea	0.550	0.489	0.061	5.85E-08	5.41E-06	46	144	65
cg09722609	5	149887422	NDST1	TSS1500	OpenSea	0.607	0.551	0.055	6.05E-08	5.54E-06	48	261	48
cg04339360	13	73635568	KLF5	Body	S_Shore	0.710	0.644	0.066	6.23E-08	5.67E-06	49	95	83
cg07264238	17	33474376	UNC45B	TSS1500	OpenSea	0.778	0.707	0.071	7.23E-08	6.32E-06	51	63	255
cg25718604	20	57601474	TUBB1	3'UTR	S_Shelf	0.831	0.774	0.057	7.34E-08	6.40E-06	52	170	61
cg00864012	17	62294665	TEX2	5'UTR	OpenSea	0.724	0.670	0.053	8.38E-08	7.01E-06	55	132	67
cg00991744	12	41581484	PDZRN4	TSS1500	N_Shore	0.852	0.791	0.061	8.42E-08	7.04E-06	56	420	21
cg08066875	2	163197084			N_Shelf	0.637	0.581	0.056	8.63E-08	7.15E-06	58	259	41

cg08324152	19	57172404			N_Shelf	0.283	0.216	0.067	9.18E-08	7.49E-06	59	399	69
cg05825244	20	2730488	EBF4	Body	Island	0.428	0.271	0.157	9.32E-08	7.58E-06	60	407	12
cg26475911	17	73056187	KCTD2	Body	OpenSea	0.458	0.390	0.067	1.14E-07	8.78E-06	65	89	142
cg26413942	5	124081751	ZNF608	TSS1500	OpenSea	0.421	0.361	0.060	1.24E-07	9.27E-06	67	202	63
cg24263062	20	2730191	EBF4	Body	Island	0.636	0.550	0.086	1.30E-07	9.59E-06	68	294	25
cg02577773	2	208027870	KLF7	Body	N_Shelf	0.652	0.588	0.064	1.56E-07	1.09E-05	73	429	11
cg00787180	14	91751731	CCDC88C	Body	N_Shelf	0.393	0.340	0.052	1.79E-07	1.21E-05	75	227	45
cg14659930	3	114128301	ZBTB20	5'UTR	OpenSea	0.694	0.626	0.068	1.80E-07	1.21E-05	76	535	16
cg19624420	14	48541885			OpenSea	0.869	0.798	0.071	2.00E-07	1.31E-05	78	385	23
cg04223006	5	82845599	VCAN	Body	OpenSea	0.433	0.362	0.072	2.23E-07	1.42E-05	80	206	28
cg27579313	17	7381622	ZBTB4;ZBTB4	5'UTR	N_Shore	0.559	0.481	0.078	2.49E-07	1.54E-05	82	146	80
cg25879365	1	40510058	CAP1	5'UTR	S_Shelf	0.598	0.517	0.081	2.73E-07	1.64E-05	84	140	78
cg06040034	8	1618448	DLGAP2	Body	S_Shore	0.780	0.719	0.061	2.80E-07	1.67E-05	85	188	75
cg09029192	17	76015204	TNRC6C	5'UTR	OpenSea	0.709	0.654	0.054	3.16E-07	1.84E-05	86	185	88
cg13518079	20	2675072	EBF4	Body	S_Shore	0.350	0.262	0.088	3.33E-07	1.90E-05	88	413	49
cg00059652	17	67605498			OpenSea	0.639	0.579	0.061	3.37E-07	1.92E-05	89	190	122
cg25671837	6	127513912	RSPO3	Body	OpenSea	0.437	0.369	0.067	3.53E-07	1.99E-05	90	332	50
cg04873188	2	70143463	MXD1	Body	S_Shore	0.567	0.494	0.073	3.91E-07	2.14E-05	91	49	219
cg25670076	6	90807675	BACH2	5'UTR	OpenSea	0.773	0.708	0.065	4.04E-07	2.19E-05	92	449	58
cg18640098	5	9888660	LOC285692	Body	OpenSea	0.869	0.807	0.063	4.05E-07	2.19E-05	93	364	279
cg02127888	16	1665900	CRAMP1L	Body	S_Shore	0.428	0.346	0.082	4.06E-07	2.20E-05	94	347	115
cg08036553	1	64649805			OpenSea	0.844	0.790	0.055	4.16E-07	2.24E-05	95	252	132
cg12039595	1	244480459			OpenSea	0.512	0.455	0.057	4.49E-07	2.36E-05	97	325	60
cg22986770	8	61648691	CHD7	5'UTR	OpenSea	0.477	0.404	0.073	4.51E-07	2.37E-05	99	47	497
cg01232748	4	54928819	CHIC2	Body	N_Shore	0.511	0.451	0.060	4.86E-07	2.50E-05	101	416	32
cg23824801	12	54653403	CBX5	TSS200;5'UTR	OpenSea	0.280	0.218	0.062	5.41E-07	2.70E-05	104	143	149
cg08173709	3	71103443	FOXP1	Body	OpenSea	0.726	0.669	0.057	5.42E-07	2.71E-05	105	212	77
cg25217710	1	156609523			N_Shelf	0.649	0.591	0.058	5.57E-07	2.76E-05	108	192	193
cg12409074	11	129870648	PRDM10	5'UTR	N_Shore	0.489	0.430	0.060	5.65E-07	2.78E-05	109	318	166
cg16356622	1	36351841	EIF2C1	Body	S_Shelf	0.522	0.466	0.056	6.43E-07	3.05E-05	111	462	68
cg10264529	14	24562064	PCK2;PCK2	TSS1500	N_Shore	0.634	0.573	0.061	6.67E-07	3.13E-05	112	256	205
cg20755748	12	31799116	C12orf72	TSS1500	OpenSea	0.572	0.515	0.057	6.73E-07	3.15E-05	113	546	64
cg02302089	7	23401203	IGF2BP3	Body	OpenSea	0.415	0.354	0.061	6.76E-07	3.15E-05	114	421	120
cg22497969	1	3143018	PRDM16	Body	OpenSea	0.784	0.728	0.057	7.34E-07	3.35E-05	116	198	159
cg05236731	13	41035061	LOC646982	Body	OpenSea	0.852	0.797	0.055	8.46E-07	3.72E-05	117	487	155
cg15847685	4	62129666			OpenSea	0.786	0.726	0.060	8.59E-07	3.76E-05	118	241	117
cg07349663	1	160261619	COPA	Body	OpenSea	0.658	0.598	0.061	8.74E-07	3.80E-05	119	180	138
cg20433822	10	124690751	C10orf88	3'UTR	OpenSea	0.278	0.214	0.063	1.09E-06	4.46E-05	123	329	106
cg25199005	1	246446300	SMYD3	Body	OpenSea	0.777	0.713	0.064	1.12E-06	4.56E-05	124	249	152
cg07143462	2	157289513			N_Shelf	0.688	0.622	0.066	1.20E-06	4.78E-05	125	550	62
cg00391741	3	127307227	TPRA1	5'UTR	N_Shore	0.735	0.679	0.056	1.25E-06	4.94E-05	128	205	161
cg07176842	14	43972223			OpenSea	0.574	0.483	0.092	1.41E-06	5.37E-05	130	377	86
cg26955132	12	13181499			OpenSea	0.744	0.673	0.070	1.46E-06	5.53E-05	132	236	176
cg16321846	7	4050114	SDK1	Body	OpenSea	0.751	0.684	0.067	1.71E-06	6.16E-05	136	390	103
cg19956914	7	56147257	SUMF2	Body	OpenSea	0.663	0.610	0.053	1.80E-06	6.42E-05	140	694	42
cg24134261	2	33661362	RASGRP3	TSS200	OpenSea	0.403	0.346	0.057	1.88E-06	6.60E-05	141	352	105
cg13485718	14	93315488			OpenSea	0.560	0.498	0.062	1.89E-06	6.62E-05	142	264	259

cg06121226	4	72134060	SLC4A4	Body	OpenSea	0.829	0.774	0.055	1.92E-06	6.69E-05	144	483	118
cg11945474	2	55717980			OpenSea	0.773	0.711	0.062	2.00E-06	6.89E-05	145	488	89
cg14989243	6	76203530	FILIP1	TSS200	OpenSea	0.527	0.452	0.075	2.08E-06	7.10E-05	147	268	162
cg12081645	4	157920476			OpenSea	0.444	0.375	0.069	2.31E-06	7.68E-05	149	956	44
cg02329430	15	73921385	NPTN	Body	N_Shelf	0.391	0.328	0.063	2.50E-06	8.13E-05	151	258	79
cg13717434	9	97854044			OpenSea	0.602	0.545	0.057	2.65E-06	8.50E-05	152	762	43
cg25015371	6	108874950			N_Shelf	0.580	0.527	0.053	2.99E-06	9.26E-05	154	800	55
cg23970645	14	89695299	FOXN3	Body	OpenSea	0.777	0.714	0.063	3.10E-06	9.52E-05	155	301	280
cg26837192	2	48813184	STON1;STON1-GTF2A1L	Body	OpenSea	0.778	0.714	0.063	3.18E-06	9.69E-05	156	555	59
cg07425204	17	66511183	PRKAR1A	5'UTR	S_Shelf	0.481	0.421	0.060	3.26E-06	9.86E-05	157	401	153
cg01282174	11	119630144			OpenSea	0.484	0.415	0.069	3.35E-06	1.01E-04	158	245	173
cg17301248	12	112467203	NAA25	3'UTR	OpenSea	0.642	0.564	0.078	3.66E-06	1.07E-04	162	375	125
cg26943120	4	5472116	STK32B	Body	OpenSea	0.631	0.574	0.057	3.68E-06	1.08E-04	163	530	134
cg12549908	10	4230567			OpenSea	0.761	0.705	0.056	3.80E-06	1.10E-04	164	220	242
cg03820688	2	55477848	MTIF2	Body	OpenSea	0.744	0.683	0.061	4.03E-06	1.15E-04	165	466	180
cg24760467	10	102760784	LZTS2	5'UTR	S_Shore	0.622	0.565	0.057	4.18E-06	1.18E-04	167	406	213
cg15034267	22	39106452	GTPBP1	Body	S_Shelf	0.768	0.700	0.068	4.36E-06	1.21E-04	169	316	325
cg27416489	2	149823115	KIF5C	Body	OpenSea	0.750	0.686	0.064	4.46E-06	1.23E-04	172	704	54
cg25688583	15	57510460	TCF12	TSS1500;Body	OpenSea	0.674	0.610	0.064	4.48E-06	1.24E-04	173	361	160
cg17568809	5	142153529	ARHGAP26	Body	S_Shelf	0.742	0.681	0.061	4.83E-06	1.31E-04	175	581	148
cg01948202	3	122400474	PARP14	Body	S_Shore	0.156	0.211	-0.054	4.85E-06	1.31E-04	176	854	34
cg05264870	2	206599315	NRP2	Body	OpenSea	0.606	0.545	0.061	4.90E-06	1.32E-04	177	441	172
cg16153042	6	3282307	SLC22A23	Body	OpenSea	0.646	0.587	0.058	5.15E-06	1.37E-04	179	306	225
cg15059804	1	33766318	ZNF362	3'UTR	OpenSea	0.731	0.649	0.082	5.34E-06	1.41E-04	180	568	73
cg19449565	2	240230892	HDAC4	Body	OpenSea	0.898	0.843	0.055	5.85E-06	1.51E-04	181	545	209
cg06899237	6	153353519	RGS17	Body	OpenSea	0.427	0.371	0.056	6.22E-06	1.57E-04	184	426	133
cg06832406	10	4230482			OpenSea	0.810	0.758	0.052	6.22E-06	1.57E-04	185	194	227
cg05475386	12	65723693	MSRB3;MSRB3	Body	OpenSea	0.304	0.243	0.061	6.25E-06	1.58E-04	187	372	246
cg22491058	1	207277466	C4BPA	TSS200	OpenSea	0.502	0.448	0.054	6.38E-06	1.60E-04	188	534	190
cg00950497	10	116393423	ABLIM1	Body	S_Shore	0.630	0.569	0.062	6.73E-06	1.67E-04	190	431	147
cg17111025	2	111936357			OpenSea	0.944	0.887	0.056	6.87E-06	1.69E-04	191	1275	174
cg05857996	20	2675418	EBF4	Body	S_Shore	0.786	0.686	0.101	6.88E-06	1.69E-04	192	697	116
cg13547053	2	179184864	OSBPL6	TSS200;Body	OpenSea	0.717	0.659	0.058	7.13E-06	1.74E-04	194	1017	33
cg03529358	17	78198361	SLC26A11	Body	S_Shelf	0.935	0.875	0.060	7.78E-06	1.85E-04	196	547	338
cg06071730	10	112021300	MXI1	Body	OpenSea	0.714	0.658	0.055	7.87E-06	1.87E-04	197	587	131
cg10366878	4	79531297	ANXA3	3'UTR	OpenSea	0.657	0.594	0.063	7.98E-06	1.88E-04	198	807	124
cg05684528	5	76473917			N_Shelf	0.636	0.577	0.060	8.11E-06	1.90E-04	199	876	108
cg10437839	11	64701910	PPP2R5B	3'UTR	OpenSea	0.657	0.604	0.052	8.11E-06	1.91E-04	200	228	305
cg08340023	16	23098187	USP31	Body	OpenSea	0.689	0.623	0.066	8.33E-06	1.94E-04	202	521	202
cg02061820	3	152046751	MBNL1	Body	OpenSea	0.459	0.385	0.074	9.36E-06	2.12E-04	206	572	158
cg27192248	15	65285669			S_Shelf	0.409	0.517	-0.108	9.67E-06	2.17E-04	208	653	94
cg07661704	4	139144433	SLC7A11	Body	OpenSea	0.561	0.496	0.065	9.74E-06	2.18E-04	209	195	391
cg15644413	1	36185939	C1orf216	TSS1500	S_Shore	0.699	0.643	0.056	9.98E-06	2.22E-04	210	464	231
cg26235748	14	86087511	FLRT2	5'UTR	OpenSea	0.531	0.454	0.077	1.01E-05	2.23E-04	211	509	261
cg10902738	8	134895500			OpenSea	0.667	0.605	0.062	1.04E-05	2.29E-04	212	771	175

cg07291349	4	40964962	APBB2	Body	OpenSea	0.643	0.572	0.071	1.07E-05	2.33E-04	213	317	494
cg04139359	1	45085438	RNF220	Body	S_Shelf	0.808	0.747	0.062	1.07E-05	2.33E-04	214	414	512
cg10196532	13	50134640	RCBTB1	Body	OpenSea	0.818	0.734	0.084	1.18E-05	2.50E-04	219	1030	144
cg26293423	5	71613149	MRPS27	Body	N_Shelf	0.749	0.695	0.054	1.19E-05	2.52E-04	220	635	98
cg00021275	2	128142155			N_Shelf	0.731	0.674	0.057	1.22E-05	2.56E-04	221	573	322
cg20141108	1	165907859			N_Shelf	0.653	0.587	0.066	1.22E-05	2.57E-04	222	637	171
cg27408285	12	54653364	CBX5	1stExon;TSS200;5'UTR	OpenSea	0.331	0.265	0.066	1.23E-05	2.58E-04	223	201	342
cg14342532	2	233916534			OpenSea	0.355	0.299	0.056	1.28E-05	2.65E-04	226	1150	76
cg07624918	3	136533970			N_Shelf	0.752	0.699	0.053	1.31E-05	2.69E-04	227	791	214
cg24235882	4	54928822	CHIC2	Body	N_Shore	0.572	0.518	0.055	1.32E-05	2.70E-04	228	1083	57
cg20211629	18	48352490	MRO	TSS1500	OpenSea	0.585	0.531	0.054	1.33E-05	2.73E-04	229	736	201
cg20675439	6	82466362			S_Shelf	0.695	0.636	0.059	1.34E-05	2.73E-04	230	610	154
cg18470427	17	33842301	SLFN12L	Body	OpenSea	0.840	0.768	0.073	1.37E-05	2.78E-04	231	324	363
cg19841423	20	62366755	ZGPAT;LIME1	Body;TSS1500	S_Shore	0.713	0.660	0.053	1.38E-05	2.80E-04	232	234	402
cg22992279	4	26414901	RBPJ	Body	OpenSea	0.745	0.691	0.054	1.39E-05	2.81E-04	233	748	177
cg05808246	1	221741553			OpenSea	0.799	0.740	0.059	1.43E-05	2.88E-04	234	295	392
cg10440877	2	208378475			OpenSea	0.723	0.666	0.057	1.47E-05	2.93E-04	235	905	112
cg08106319	6	37849427	ZFAND3	Body	OpenSea	0.381	0.309	0.073	1.50E-05	2.96E-04	236	350	223
cg01822050	20	304156			N_Shore	0.748	0.686	0.062	1.50E-05	2.97E-04	237	1053	82
cg16778107	16	381873	AXIN1	Body	OpenSea	0.511	0.451	0.059	1.52E-05	2.99E-04	239	808	189
cg05739816	6	135989395	C6orf217	Body	OpenSea	0.534	0.459	0.074	1.53E-05	3.01E-04	240	417	345
cg11952493	10	28570835	MPP7	5'UTR	OpenSea	0.744	0.693	0.051	1.58E-05	3.09E-04	241	579	248
cg14819618	8	8180214	PRAGMIN	Body	S_Shelf	0.793	0.729	0.063	1.67E-05	3.20E-04	242	313	448
cg02543850	11	33346306	HIPK3	Body	OpenSea	0.595	0.539	0.057	1.70E-05	3.25E-04	244	418	230
cg18784409	11	67868331	CHKA	Body	OpenSea	0.509	0.456	0.053	1.78E-05	3.36E-04	247	270	317
cg05850338	17	79933123			N_Shelf	0.686	0.623	0.062	1.78E-05	3.36E-04	248	553	267
cg24786658	7	112727423	GPR85	TSS1500;5'UTR	OpenSea	0.705	0.647	0.058	1.90E-05	3.52E-04	251	498	340
cg17781958	17	79428404	BAHCC1	Body	Island	0.758	0.702	0.057	1.93E-05	3.56E-04	252	133	556
cg15931921	6	170597588	DLL1	Body	N_Shore	0.748	0.669	0.080	1.95E-05	3.59E-04	253	894	203
cg03680873	3	148844300			N_Shelf	0.600	0.535	0.065	2.04E-05	3.71E-04	255	388	244
cg20176142	8	81408357	ZBTB10	Body	OpenSea	0.728	0.678	0.050	2.11E-05	3.79E-04	256	949	156
cg12114888	8	107738435	OXR1	Body	OpenSea	0.336	0.283	0.053	2.28E-05	4.02E-04	257	711	169
cg06857116	17	15885326	ZSWIM7	Body	OpenSea	0.594	0.540	0.054	2.36E-05	4.11E-04	260	624	278
cg18638434	14	74486651	C14orf45;ENTPD5	Body;TSS1500	S_Shore	0.732	0.670	0.063	2.39E-05	4.14E-04	263	768	299
cg26136772	7	4754681	FOXK1	Body	N_Shelf	0.708	0.647	0.061	2.54E-05	4.33E-04	267	873	300
cg04567302	6	31846956	SLC44A4	TSS200	OpenSea	0.789	0.730	0.059	2.60E-05	4.40E-04	269	274	456
cg00084338	6	170595920	DLL1	Body	N_Shore	0.598	0.495	0.103	2.67E-05	4.49E-04	272	855	197
cg18388786	10	134924050	GPR123	Body	N_Shore	0.312	0.254	0.058	2.70E-05	4.51E-04	273	344	413
cg27246129	6	170595947	DLL1	Body	N_Shore	0.550	0.480	0.070	2.71E-05	4.52E-04	274	759	224
cg11254053	1	33497125	AK2;AK2	Body	OpenSea	0.554	0.496	0.058	2.77E-05	4.59E-04	278	924	206
cg00634542	2	219254588	SLC11A1	Body	N_Shore	0.556	0.495	0.062	2.85E-05	4.70E-04	279	221	470
cg19723805	5	33147539			OpenSea	0.452	0.396	0.055	2.92E-05	4.77E-04	280	890	249
cg05142445	14	76309157	TTLL5	Body	OpenSea	0.716	0.657	0.059	3.04E-05	4.92E-04	282	789	127
cg12559197	5	76654783	PDE8B	Body	OpenSea	0.515	0.440	0.074	3.21E-05	5.10E-04	283	1105	95
cg07539709	17	80545454	FOXK2	Body	Island	0.598	0.538	0.060	3.24E-05	5.15E-04	285	788	198

cg14324502	9	88898587	ISCA1	TSS1500	S_Shore	0.555	0.489	0.066	3.31E-05	5.23E-04	286	351	506
cg13573513	12	14996143	ART4;ART4	5'UTR;1stExon	OpenSea	0.763	0.698	0.065	3.49E-05	5.42E-04	289	330	694
cg18067134	3	71084634	FOXP1	Body	OpenSea	0.728	0.667	0.061	3.56E-05	5.50E-04	290	543	296
cg03529189	12	64292138	SRGAP1	Body	OpenSea	0.743	0.689	0.053	3.60E-05	5.55E-04	293	753	337
cg23711422	21	43442085	C21orf121	TSS200	OpenSea	0.502	0.430	0.073	3.66E-05	5.62E-04	294	1172	298
cg08739755	2	172815385	HAT1	Body	OpenSea	0.803	0.737	0.066	3.82E-05	5.78E-04	295	1291	266
cg19560014	12	31799118	C12orf72	TSS1500	OpenSea	0.614	0.554	0.061	3.86E-05	5.83E-04	296	434	642
cg10584024	1	84234998			OpenSea	0.541	0.469	0.072	3.95E-05	5.92E-04	300	654	328
cg07951201	13	103340215	C13orf39	Body	OpenSea	0.444	0.378	0.065	4.01E-05	5.98E-04	304	937	236
cg12647970	6	3234297			S_Shelf	0.805	0.750	0.055	4.23E-05	6.22E-04	305	778	344
cg05233670	12	109058033	CORO1C	Body	OpenSea	0.479	0.415	0.064	4.24E-05	6.23E-04	306	887	354
cg08707875	1	224575077	WDR26	3'UTR	OpenSea	0.702	0.646	0.056	4.31E-05	6.31E-04	307	763	294
cg21781157	20	47874111	ZNFX1	Body	OpenSea	0.530	0.468	0.063	4.37E-05	6.37E-04	308	783	204
cg17846127	15	89940505	LOC254559	Body	N_Shelf	0.688	0.632	0.056	4.39E-05	6.38E-04	309	265	697
cg06663615	17	79423959	BAHCC1	Body	N_Shore	0.565	0.513	0.052	4.42E-05	6.42E-04	310	515	460
cg06633438	19	6272158	MLLT1	Body	Island	0.606	0.525	0.080	4.67E-05	6.66E-04	314	1319	121
cg22596016	17	61711557	MAP3K3	Body	OpenSea	0.266	0.201	0.065	4.82E-05	6.82E-04	315	824	128
cg17129188	8	134307597	NDRG1	5'UTR	N_Shore	0.774	0.722	0.052	4.82E-05	6.82E-04	316	779	222
cg18115721	11	73567838	MRPL48	Body	OpenSea	0.807	0.751	0.056	5.02E-05	7.01E-04	317	668	359
cg02112168	14	45579561	PRPF39;SNORD127	Body;TSS1500	OpenSea	0.663	0.592	0.070	5.06E-05	7.06E-04	318	1242	168
cg27134944	13	76111107	COMMD6	Body	N_Shore	0.560	0.501	0.059	5.10E-05	7.09E-04	319	1352	99
cg08064068	14	60671024			OpenSea	0.663	0.603	0.060	5.15E-05	7.14E-04	320	819	320
cg24530147	3	138763894	PRR23C	TSS200	Island	0.773	0.707	0.066	5.35E-05	7.34E-04	322	323	422
cg26104475	5	169693176	LCP2	Body	OpenSea	0.640	0.584	0.056	5.45E-05	7.43E-04	324	931	235
cg07769015	8	142238770	SLC45A4	TSS200	S_Shore	0.624	0.568	0.057	5.55E-05	7.53E-04	327	461	486
cg13607230	2	103352296	MFSD9	Body	N_Shore	0.473	0.404	0.069	5.56E-05	7.55E-04	329	689	187
cg08481967	6	166765599			OpenSea	0.358	0.272	0.086	5.59E-05	7.57E-04	330	353	617
cg17510957	11	121466629	SORL1	Body	OpenSea	0.700	0.637	0.063	5.61E-05	7.59E-04	331	840	215
cg22249612	12	56121485	CD63	Body	N_Shore	0.659	0.589	0.069	5.65E-05	7.63E-04	332	556	532
cg20925233	17	47661136	NXPH3	3'UTR	OpenSea	0.563	0.505	0.059	5.84E-05	7.81E-04	334	442	638
cg17164954	6	157345266	ARID1B	Body	S_Shelf	0.457	0.396	0.061	6.06E-05	8.02E-04	335	598	384
cg16805291	7	36022575			Island	0.692	0.623	0.069	6.14E-05	8.08E-04	336	340	323
cg06190046	16	83986382	OSGIN1	5'UTR;TSS1500;Body	OpenSea	0.500	0.427	0.074	6.26E-05	8.20E-04	337	1108	250
cg06487194	6	15345097	JARID2	Body	OpenSea	0.589	0.525	0.064	6.27E-05	8.21E-04	338	642	281
cg20043649	2	47537239			OpenSea	0.699	0.636	0.064	6.60E-05	8.50E-04	339	690	417
cg14044785	2	43560368	THADA;THADA	Body	OpenSea	0.692	0.623	0.069	6.70E-05	8.60E-04	341	526	459
cg16702362	2	135078044	MGAT5	Body	OpenSea	0.719	0.654	0.064	6.94E-05	8.82E-04	342	969	257
cg24165638	19	831456	AZU1	Body	N_Shelf	0.640	0.586	0.054	7.00E-05	8.88E-04	344	281	672
cg09803321	10	104913480	NT5C2	Body	OpenSea	0.727	0.670	0.057	7.10E-05	8.96E-04	345	1244	207
cg10805254	3	72433837	RYBP	Body	OpenSea	0.581	0.518	0.063	7.58E-05	9.38E-04	349	614	371
cg02209770	20	35062903	DLGAP4	Body	N_Shore	0.553	0.502	0.051	7.80E-05	9.58E-04	351	1103	330
cg05687149	11	112035945	IL18	TSS1500	OpenSea	0.278	0.227	0.052	7.82E-05	9.60E-04	352	850	273
cg27416337	3	32748004	CNOT10	Body	OpenSea	0.343	0.280	0.063	8.04E-05	9.79E-04	353	494	514
cg03305017	7	151036715			N_Shelf	0.494	0.434	0.060	8.07E-05	9.81E-04	355	595	573

cg25263801	7	112727506	GPR85	TSS1500;1stExon;5'UTR	OpenSea	0.719	0.664	0.055	8.11E-05	9.85E-04	356	615	432
cg07772516	15	52107742	TMOD2	3'UTR	OpenSea	0.611	0.537	0.074	8.54E-05	1.02E-03	360	929	339
cg13127231	13	111806949	ARHGEF7	Body;5'UTR	S_Shore	0.459	0.409	0.050	8.59E-05	1.03E-03	361	870	277
cg21579539	18	43751229			N_Shelf	0.709	0.656	0.052	8.78E-05	1.04E-03	362	1064	233
cg25757140	19	38039469			N_Shore	0.611	0.553	0.058	9.06E-05	1.07E-03	364	601	424
cg11210069	5	140761259	PCDHGA4;PCDHGA6;PCDHGA1;PCDHGA5;PCDHGB1;PCDHGA3;PCDHGA2;PCDHGB2;PCDHGA7;PCDHGA7;PCDHGB3	Body;TSS1500	N_Shore	0.699	0.641	0.058	9.15E-05	1.07E-03	366	463	541
cg05581469	12	49413435	MLL2;PRKAG1	3'UTR;TSS1500	S_Shore	0.368	0.313	0.055	9.21E-05	1.08E-03	367	918	318
cg09305680	8	117778069	UTP23	TSS1500	N_Shore	0.600	0.545	0.055	9.26E-05	1.08E-03	368	548	588
cg10976975	2	69098569	BMP10;BMP10	5'UTR;1stExon	OpenSea	0.714	0.649	0.065	9.62E-05	1.11E-03	370	833	290
cg23679982	17	1686737	SMYD4	Body	OpenSea	0.636	0.570	0.066	9.76E-05	1.12E-03	371	473	724
cg24926791	6	31082187	PSORS1C1	TSS1500	OpenSea	0.653	0.483	0.170	1.01E-04	1.15E-03	374	939	313
cg05916684	17	49008322			N_Shore	0.503	0.444	0.059	1.05E-04	1.18E-03	381	1092	170
cg24590430	10	99097076			S_Shelf	0.792	0.718	0.074	1.06E-04	1.19E-03	383	841	641
cg21697512	1	208081541	CD34	Body	N_Shelf	0.575	0.515	0.060	1.07E-04	1.20E-03	384	1563	186
cg01238669	7	21797276	DNAH11	Body	OpenSea	0.446	0.386	0.060	1.09E-04	1.21E-03	385	851	393
cg23098068	2	172650722	SLC25A12	Body	OpenSea	0.420	0.343	0.077	1.15E-04	1.26E-03	386	471	620
cg06740950	3	171878318	FNDC3B	Body	OpenSea	0.473	0.416	0.057	1.19E-04	1.29E-03	388	1213	217
cg08131499	12	109090588	CORO1C	Body	OpenSea	0.308	0.247	0.061	1.20E-04	1.29E-03	389	1146	210
cg02291010	5	96079433	CAST	Body;1stExon	OpenSea	0.669	0.612	0.057	1.21E-04	1.30E-03	391	885	367
cg10789050	3	31713847	OSBPL10	Body	OpenSea	0.509	0.425	0.084	1.21E-04	1.30E-03	393	693	451
cg17797863	4	2009396	WHSC2	Body	N_Shore	0.542	0.489	0.052	1.21E-04	1.31E-03	395	908	365
cg02457461	8	9913680	MSRA	Body	S_Shore	0.251	0.193	0.058	1.27E-04	1.34E-03	398	1241	100
cg06392753	2	102783282	IL1R1	Body	OpenSea	0.549	0.488	0.061	1.29E-04	1.36E-03	399	695	447
cg19947463	7	1113237	C7orf50	Body	OpenSea	0.459	0.384	0.075	1.30E-04	1.37E-03	400	1416	306
cg24265969	3	55654481	ERC2	3'UTR	OpenSea	0.322	0.262	0.060	1.33E-04	1.39E-03	402	1015	307
cg02769705	1	202975059	TMEM183B;TMEM183A	TSS1500	N_Shore	0.588	0.520	0.069	1.35E-04	1.41E-03	403	574	596
cg27301230	7	65761563	TPST1	Body	OpenSea	0.737	0.682	0.055	1.35E-04	1.41E-03	404	663	498
cg18281939	5	77783895	LHFPL2	3'UTR	OpenSea	0.452	0.390	0.062	1.38E-04	1.43E-03	405	1111	304
cg06794244	2	196434818			OpenSea	0.470	0.416	0.054	1.40E-04	1.44E-03	408	1482	218
cg21932231	2	36668864	CRIM1	Body	OpenSea	0.543	0.488	0.055	1.43E-04	1.47E-03	410	1304	264
cg00819078	5	16615081	FAM134B	Body	N_Shore	0.686	0.627	0.059	1.48E-04	1.50E-03	411	404	546
cg01014262	16	48533930			S_Shelf	0.354	0.294	0.060	1.49E-04	1.51E-03	412	713	409
cg24238409	10	93998677	CPEB3	Body	N_Shore	0.759	0.704	0.055	1.51E-04	1.52E-03	414	842	662
cg00796963	6	84936558	KIAA1009	5'UTR	N_Shore	0.518	0.463	0.055	1.61E-04	1.59E-03	420	1309	276
cg21487561	2	11990393			OpenSea	0.916	0.850	0.066	1.62E-04	1.60E-03	421	1612	353

cg23362032	7	126990845			S_Shelf	0.770	0.717	0.052	1.62E-04	1.60E-03	422	1091	331
cg27586596	7	41426609			OpenSea	0.444	0.390	0.054	1.64E-04	1.61E-03	423	730	455
cg03854238	6	22071933	FLJ22536	Body	OpenSea	0.619	0.554	0.065	1.65E-04	1.62E-03	424	826	263
cg25287268	13	50694004	DLEU2	Body	N_Shelf	0.439	0.379	0.060	1.66E-04	1.63E-03	425	1391	245
cg01550445	11	72929983	P2RY2	5'UTR	S_Shore	0.633	0.581	0.052	1.67E-04	1.63E-03	426	1178	284
cg25589001	10	98605052	LCOR	5'UTR	OpenSea	0.557	0.501	0.055	1.81E-04	1.73E-03	434	726	400
cg24159514	19	11785248	ZNF833	Body	Island	0.454	0.378	0.076	1.84E-04	1.74E-03	435	756	442
cg00421164	6	7111068	RREB1	5'UTR	S_Shore	0.827	0.774	0.053	1.90E-04	1.79E-03	436	1099	396
cg10368935	10	18240316	SLC39A12;SLC39A12	TSS1500	OpenSea	0.733	0.666	0.066	1.97E-04	1.83E-03	437	602	416
cg14325153	3	138763899	PRR23C	TSS200	Island	0.537	0.470	0.067	2.01E-04	1.86E-03	439	287	594
cg19619014	8	37250930			OpenSea	0.392	0.331	0.061	2.04E-04	1.88E-03	440	1169	327
cg26861460	22	44575455	PARVG	TSS1500;5'UTR;Body	OpenSea	0.528	0.474	0.054	2.06E-04	1.89E-03	443	1154	394
cg18854872	2	198292151	SF3B1	Body	OpenSea	0.631	0.576	0.056	2.06E-04	1.89E-03	444	1204	311
cg01966791	20	62572875	MIR1914;UCKL1	Body	S_Shore	0.479	0.411	0.068	2.08E-04	1.91E-03	445	912	315
cg07023317	8	28961315	KIF13B	Body	OpenSea	0.740	0.688	0.053	2.13E-04	1.93E-03	447	998	482
cg03673989	11	380069	B4GALNT4	Body	Island	0.884	0.828	0.055	2.14E-04	1.94E-03	448	1175	505
cg12884009	2	219079038			N_Shelf	0.780	0.729	0.051	2.21E-04	1.99E-03	449	1435	272
cg24142603	8	72753888	MSC	3'UTR	Island	0.789	0.851	-0.062	2.28E-04	2.03E-03	451	1473	228
cg14312063	3	85010606	CADM2	Body	S_Shore	0.505	0.443	0.062	2.30E-04	2.05E-03	452	612	618
cg11058932	7	130372167	TSGA13	TSS1500	OpenSea	0.706	0.649	0.057	2.39E-04	2.10E-03	456	1132	465
cg04777091	4	6050440	JAKMIP1	Body	OpenSea	0.229	0.172	0.058	2.41E-04	2.11E-03	457	479	558
cg13059136	11	2986541	SNORA54;NAP1L4	TSS1500;Body	OpenSea	0.664	0.607	0.056	2.47E-04	2.15E-03	460	828	476
cg21078654	15	52130305	TMOD3	5'UTR	OpenSea	0.287	0.233	0.054	2.48E-04	2.16E-03	461	1059	559
cg05351887	16	3988869			N_Shore	0.425	0.362	0.063	2.55E-04	2.20E-03	463	696	537
cg22716738	7	27784932	TAX1BP1	5'UTR	OpenSea	0.424	0.359	0.064	2.67E-04	2.27E-03	471	907	651
cg25338454	12	26900022	ITPR2	Body	OpenSea	0.764	0.706	0.058	2.76E-04	2.32E-03	473	776	685
cg21992696	2	9125026	MBOAT2	Body	OpenSea	0.429	0.362	0.066	2.78E-04	2.33E-03	474	821	598
cg06122613	1	151297900	PI4KB	Body	N_Shelf	0.757	0.700	0.057	2.80E-04	2.34E-03	475	881	643
cg23705979	7	92397161	CDK6	Body	OpenSea	0.354	0.301	0.053	2.83E-04	2.36E-03	476	1137	378
cg05697976	12	29376483	FAR2	TSS200	OpenSea	0.518	0.453	0.066	2.86E-04	2.38E-03	478	934	689
cg13088432	8	9522664	TNKS	Body	OpenSea	0.570	0.498	0.072	2.87E-04	2.39E-03	479	801	561
cg11168614	14	87179368			OpenSea	0.651	0.588	0.063	2.87E-04	2.39E-03	480	1502	262
cg14111928	10	76602391	MYST4	5'UTR	OpenSea	0.711	0.655	0.056	2.93E-04	2.42E-03	482	1251	582
cg04231677	1	184808004	FAM129A	Body	OpenSea	0.519	0.453	0.066	2.95E-04	2.43E-03	483	1550	268
cg09982419	15	68343575			N_Shelf	0.641	0.582	0.059	3.00E-04	2.46E-03	484	731	721
cg05888181	8	1952319	KBTBD11	3'UTR	S_Shore	0.666	0.598	0.068	3.06E-04	2.50E-03	485	649	602
cg13424302	10	80516893			OpenSea	0.417	0.359	0.058	3.06E-04	2.50E-03	486	871	429
cg08195176	11	116781303	SIK3	Body	OpenSea	0.604	0.551	0.053	3.07E-04	2.50E-03	487	1396	289
cg03633574	3	149954846			OpenSea	0.804	0.752	0.052	3.22E-04	2.59E-03	493	1273	287
cg05454562	6	33254447	WDR46	Body	N_Shelf	0.624	0.566	0.058	3.41E-04	2.69E-03	499	1441	369
cg20610950	17	75096202			OpenSea	0.765	0.700	0.065	3.43E-04	2.70E-03	501	1594	303
cg08598221	8	121824929	SNTB1	TSS1500	S_Shore	0.589	0.534	0.055	3.49E-04	2.73E-03	503	1492	253
cg11952340	15	63814440	USP3	Body	OpenSea	0.604	0.547	0.056	3.49E-04	2.73E-03	504	879	585
cg10499974	3	46244099	CCR1	3'UTR	OpenSea	0.668	0.614	0.054	3.52E-04	2.75E-03	506	1102	673

cg26105956	1	154471433	SHE	Body	N_Shelf	0.733	0.668	0.065	3.52E-04	2.75E-03	507	1042	351
cg06645778	2	64680425	HSPC159	TSS1500	N_Shore	0.565	0.513	0.052	3.62E-04	2.80E-03	510	1657	226
cg02320474	16	1758022	MAPK8IP3	Body	OpenSea	0.434	0.371	0.063	3.65E-04	2.82E-03	512	970	496
cg26253500	7	136641740	CHRM2	5'UTR	OpenSea	0.758	0.703	0.055	3.84E-04	2.92E-03	517	1321	386
cg21216258	9	140942584	CACNA1B	Body	S_Shore	0.590	0.518	0.073	3.87E-04	2.93E-03	518	1330	435
cg07805500	6	151380818	MTHFD1L	Body	OpenSea	0.480	0.391	0.089	4.05E-04	3.02E-03	522	1026	426
cg26828017	16	22409023			OpenSea	0.684	0.623	0.060	4.15E-04	3.07E-03	524	1100	619
cg07229212	8	11056726	XKR6	Body	N_Shore	0.605	0.546	0.059	4.18E-04	3.09E-03	525	1049	521
cg07113414	8	144652930	C8orf73	Body	N_Shore	0.551	0.485	0.065	4.19E-04	3.10E-03	527	1516	518
cg12237948	20	2085157	STK35	Body	S_Shore	0.724	0.662	0.062	4.20E-04	3.10E-03	528	1041	452
cg22159528	19	44039727	ZNF575	Body	Island	0.605	0.551	0.055	4.23E-04	3.12E-03	531	1142	372
cg05279761	3	132312243	ACAD11	Body	OpenSea	0.568	0.498	0.069	4.28E-04	3.14E-03	532	897	640
cg21426759	6	30303126	TRIM39	Body	OpenSea	0.656	0.603	0.053	4.42E-04	3.21E-03	536	1472	484
cg13640297	6	170099238	WDR27	5'UTR	N_Shelf	0.676	0.625	0.051	4.42E-04	3.22E-03	537	1571	309
cg07541020	10	44068714	ZNF239	5'UTR	N_Shore	0.703	0.640	0.063	4.46E-04	3.23E-03	539	1598	366
cg01924292	12	1815334	ADIPOR2	5'UTR	OpenSea	0.656	0.584	0.072	4.52E-04	3.26E-03	542	1449	383
cg00117012	15	73996358	CD276	Body	OpenSea	0.810	0.708	0.102	4.74E-04	3.37E-03	547	1062	431
cg22630754	15	42866523	STARD9	TSS1500	N_Shore	0.687	0.626	0.061	5.04E-04	3.52E-03	550	1540	373
cg07948875	1	169187004	NME7	Body	OpenSea	0.314	0.257	0.058	5.15E-04	3.57E-03	551	1480	352
cg08760493	4	109994039	COL25A1	Body	OpenSea	0.533	0.480	0.053	5.18E-04	3.59E-03	553	1349	535
cg11321181	19	17721489	UNC13A	Body	S_Shelf	0.374	0.310	0.063	5.35E-04	3.67E-03	558	950	709
cg10603275	3	239665	CHL1	5'UTR	Island	0.180	0.125	0.055	5.49E-04	3.73E-03	560	1562	183
cg18507018	8	92035517	TMEM55A	Body	OpenSea	0.726	0.654	0.072	5.52E-04	3.75E-03	561	1025	609
cg24406240	11	88153520			OpenSea	0.614	0.549	0.065	5.57E-04	3.77E-03	565	1547	421
cg14308082	10	131568021			OpenSea	0.596	0.542	0.054	5.68E-04	3.82E-03	567	569	730
cg26975524	8	1713012	CLN8	5'UTR	S_Shore	0.647	0.587	0.060	5.74E-04	3.85E-03	571	1405	444
cg09069499	8	26216910	PPP2R2A	Body	OpenSea	0.770	0.713	0.057	5.80E-04	3.88E-03	575	1609	379
cg19116959	4	146841472	ZNF827	Body	OpenSea	0.479	0.421	0.058	5.81E-04	3.89E-03	576	822	681
cg21597811	8	123687197			OpenSea	0.685	0.631	0.054	5.84E-04	3.89E-03	577	1095	493
cg12437013	13	114161939	TMCO3	Body	N_Shelf	0.647	0.556	0.091	5.84E-04	3.90E-03	578	1400	238
cg26162326	7	75957061	YWHAG	3'UTR	N_Shore	0.507	0.442	0.064	5.85E-04	3.90E-03	579	872	543
cg26872780	2	149478494	EPC2	Body	OpenSea	0.631	0.571	0.059	5.90E-04	3.92E-03	581	1556	405
cg02282631	5	42953543			S_Shore	0.696	0.635	0.060	6.03E-04	3.98E-03	584	1018	515
cg03967798	4	145268453			OpenSea	0.535	0.481	0.054	6.09E-04	4.01E-03	585	1130	637
cg04998202	1	61545546	NFIA	Body	N_Shelf	0.399	0.333	0.066	6.19E-04	4.05E-03	586	802	564
cg19238325	3	51988425	GPR62	TSS1500	N_Shore	0.386	0.335	0.051	6.29E-04	4.10E-03	587	994	702
cg16520357	13	53625240	OLFM4	3'UTR	OpenSea	0.726	0.667	0.059	6.37E-04	4.13E-03	589	989	699
cg04350202	10	111653363	XPNPEP1	Body	OpenSea	0.554	0.501	0.053	6.43E-04	4.16E-03	592	1495	549
cg25865108	6	45559104			OpenSea	0.668	0.606	0.062	6.71E-04	4.28E-03	595	997	659
cg03269667	3	9744891	CPNE9	TSS1500	N_Shore	0.920	0.819	0.101	6.75E-04	4.30E-03	598	1626	380
cg09643312	2	160655081	CD302	TSS1500	S_Shore	0.492	0.434	0.057	6.86E-04	4.35E-03	600	1423	499
cg09101062	5	43487508	C5orf34	Body	S_Shelf	0.857	0.794	0.062	6.96E-04	4.40E-03	603	1676	377
cg05696877	1	79088769	IFI44L	5'UTR	OpenSea	0.449	0.521	-0.072	7.33E-04	4.56E-03	609	1334	437
cg03950493	11	126582183	KIRREL3	Body	OpenSea	0.516	0.443	0.073	7.49E-04	4.62E-03	611	1094	406
cg23395177	14	77498304			N_Shore	0.693	0.585	0.109	7.51E-04	4.63E-03	612	1046	539
cg03818715	6	7591348	SNRNP48	Body	S_Shore	0.588	0.528	0.059	7.62E-04	4.68E-03	614	1070	593
cg21341487	20	60760919	GTPBP5	5'UTR	S_Shelf	0.497	0.440	0.057	7.71E-04	4.71E-03	617	1281	492
cg09858862	1	23887514	ID3	TSS1500	S_Shore	0.585	0.518	0.067	7.80E-04	4.75E-03	620	1106	663

cg25656283	10	50733196	ERCC6;PGBD3	Body;TSS1500	OpenSea	0.762	0.709	0.053	7.99E-04	4.83E-03	623	1369	433
cg04139465	19	51020297	LRRC4B	3'UTR	Island	0.540	0.468	0.071	8.06E-04	4.86E-03	625	1410	412
cg01701649	16	48589954	N4BP1	Body	OpenSea	0.382	0.316	0.067	8.27E-04	4.95E-03	627	1623	333
cg15084543	1	79472408	ELTD1	5'UTR;1stExon	Island	0.360	0.298	0.061	8.44E-04	5.02E-03	628	1451	314
cg24361586	17	62744534			OpenSea	0.709	0.653	0.056	8.53E-04	5.06E-03	629	1643	527
cg09506675	7	112727914	GPR85	TSS1500;TSS200;TSS200	OpenSea	0.546	0.491	0.055	8.81E-04	5.17E-03	637	1153	579
cg09357350	3	171894094	FNDC3B	Body	OpenSea	0.641	0.583	0.058	9.07E-04	5.27E-03	644	1456	563
cg23670353	1	59833489	FGGY	Body	OpenSea	0.540	0.479	0.061	9.17E-04	5.31E-03	646	1584	567
cg02884928	14	105251610	AKT1	Body	Island	0.600	0.543	0.058	9.20E-04	5.33E-03	648	1272	738
cg09729012	3	72395774			OpenSea	0.396	0.325	0.071	9.46E-04	5.43E-03	655	1161	696
cg17797940	3	171759519	FNDC3B	5'UTR	S_Shore	0.539	0.479	0.061	9.52E-04	5.46E-03	656	619	645
cg19214707	7	3157722			OpenSea	0.398	0.238	0.160	9.66E-04	5.51E-03	660	1494	531
cg15843217	4	699224	PCGF3	TSS1500	N_Shore	0.442	0.390	0.052	9.68E-04	5.52E-03	661	1266	415
cg26132114	10	44223934			Island	0.714	0.659	0.055	1.02E-03	5.71E-03	672	1060	589
cg22283115	7	4911121	RADIL	Body	OpenSea	0.576	0.523	0.053	1.07E-03	5.92E-03	679	1165	458
cg06193043	1	11908199	NPPA	TSS1500	OpenSea	0.524	0.472	0.052	1.08E-03	5.96E-03	681	1217	635
cg06347643	2	137518979			N_Shelf	0.928	0.855	0.073	1.09E-03	6.00E-03	683	1566	718
cg23605961	7	751331	PRKAR1B	5'UTR	N_Shore	0.553	0.493	0.060	1.12E-03	6.10E-03	686	1434	679
cg07319315	2	135149599	MGAT5	Body	OpenSea	0.706	0.642	0.064	1.12E-03	6.13E-03	688	1240	529
cg20168849	3	188995030	TPRG1	Body	OpenSea	0.591	0.527	0.064	1.13E-03	6.17E-03	691	1529	725
cg00261690	1	28856281	SNHG3-RCC1;RCC1	Body;5'UTR	N_Shore	0.613	0.553	0.060	1.14E-03	6.19E-03	694	1432	669
cg18517369	2	231899207			N_Shelf	0.596	0.543	0.053	1.15E-03	6.22E-03	695	1668	443
cg24856673	12	2017134	CACNA2D4	Body	Island	0.343	0.283	0.060	1.17E-03	6.29E-03	697	1225	500
cg02262553	4	87849250			OpenSea	0.506	0.453	0.052	1.18E-03	6.33E-03	699	1678	578
cg15073631	13	104796427			OpenSea	0.379	0.329	0.051	1.19E-03	6.36E-03	701	1546	688
cg14942952	22	44576268	PARVG	TSS1500;5'UTR;Body	OpenSea	0.588	0.523	0.066	1.19E-03	6.38E-03	702	1628	603
cg05131696	12	31499320			OpenSea	0.441	0.373	0.068	1.19E-03	6.38E-03	703	1379	554
cg22629528	11	132951861	OPCML	Body	Island	0.638	0.578	0.060	1.29E-03	6.72E-03	711	837	632
cg08730245	12	53902893	NPFF	TSS1500	OpenSea	0.645	0.590	0.056	1.32E-03	6.84E-03	715	1548	687
cg01664300	6	15911690			OpenSea	0.703	0.589	0.114	1.32E-03	6.84E-03	716	1442	608
cg00782708	2	44933278	C2orf34	Body	OpenSea	0.635	0.581	0.053	1.40E-03	7.14E-03	721	1429	736
cg11147155	1	150595206	ENSA	3'UTR	OpenSea	0.493	0.442	0.051	1.48E-03	7.41E-03	729	1522	675
cg20155875	17	66452567	WIPI1	Body	N_Shore	0.620	0.563	0.057	1.52E-03	7.54E-03	738	1692	711
cg10231675	12	2944493	NRIP2	TSS1500	OpenSea	0.596	0.537	0.059	1.60E-03	7.83E-03	742	1378	729
cg08113187	16	87469329	ZCCHC14	Body	OpenSea	0.251	0.196	0.055	1.61E-03	7.83E-03	743	1306	568
cg11740416	3	194409431	FAM43A	3'UTR;1stExon	S_Shore	0.321	0.259	0.062	1.64E-03	7.95E-03	745	1606	479
cg13480937	1	177112230	ASTN1	Body	OpenSea	0.500	0.433	0.066	1.68E-03	8.07E-03	752	1385	698
cg20824294	3	142316082	PLS1	5'UTR	S_Shore	0.582	0.520	0.062	1.72E-03	8.22E-03	755	1197	684
cg01327169	3	183738470			S_Shelf	0.703	0.650	0.053	1.86E-03	8.68E-03	771	1318	542
cg08920032	15	99332004	IGF1R	Body	OpenSea	0.623	0.568	0.055	2.51E-03	1.07E-02	820	1357	732
cg22962883	11	114178729	NNMT	Body	OpenSea	0.410	0.341	0.069	2.83E-03	1.16E-02	839	1634	473
cg05991492	16	3988700			N_Shore	0.499	0.440	0.059	3.19E-03	1.26E-02	863	1625	624
cg14076239	13	53712850			OpenSea	0.617	0.564	0.053	3.48E-03	1.34E-02	880	1616	690

Table S7. Amplicons selected for targeted bisulfite sequencing

Chr	Gene	Justification for selection	Number of amplicons selected
2	XDH	#1 site in site approach, also identified in region approach	1 amplicon
13	SPERT	#2 site in site approach, also identified in region approach	1 amplicon
16	NLRC5	#3 site in site approach, also identified in region approach	2 amplicons
20	EBF4	6 adjacent sites in site approach, also identified in region approach	6 amplicons
3	FOXP1	4 adjacent sites in site approach	2 amplicons
3	FNDC3B	4 adjacent sites in site approach	1 amplicon
6	RPS6KA2	4 adjacent sites in site approach	1 amplicon
6	DLL1	Top 10 in region approach; also identified in site approach	1 amplicon
6	HCG22	Top 10 in region approach; also identified in site approach	1 amplicon

Table S8. Probes filtered in pre-processing

Filtering step	Description	Probes removed
Control probes	Removed 65 control probes	65
Non-CpG probes	Removed non-CPG probes including CAG (n=1050), CAH (n=144), and CTG (n=7)	1,266
Failed probes	Removed poor quality probes with Greedycut, an iterative algorithm that filters out probes with the highest fraction of unreliable measurements one at a time	1,602
Single nucleotide polymorphism (SNP)-enriched probes	Remove probes that overlap with SNPs	79,432
Cross-reactive probes	Removed probes with high likelihood of cross-hybridization	23,038
Sex chromosome (non-autosomal) probes	Removed probes at X and Y chromosomes	9,556

Table S9. Comparison of estimated mean (SD) cell percentages of white blood cells in our study to two pediatric references and mean (SD) differentials from a prior study by our group

Current study				Reference			
**Cell Type**[1]	**All (N=179)**	**HIV-infected (N=119)**	**HIV-uninfected (N=60)**	**Cell Type**	**Harriet Lane**[2]	**BARC**[3]	**Prior study**[4]
Lymphocytes	53.2 (14.8)	54.9 (15.4)	49.9 (12.8)	Lymphocytes	38-50	42	48.6 (12.3)
Monocytes	7.1 (3.2)	6.9 (3.2)	7.5 (3.3)	Monocytes	4-5	5	8.0 (2.5)
Granulocytes	39.7 (13.0)	38.3 (13.6)	42.5 (11.2)	Neutrophils	42-54	48	39.8 (12.6)
				Eosinophils	2-3	3	3.0 (3.3)
				Basophils	NA	2	0.55 (0.4)

[1]Lymphocytes includes $CD8^+$ T-cells, $CD4^+$ T-cells, Natural killer cells, and B-cells; granulocytes includes neutrophils and eosinophils

[2]Pediatric reference for children ages 4-9 years from the Harriet Lane Handbook

[3]Pediatric reference from BARC Global Central Laboratories for children ages 6-10 years for lymphocytes and neutrophils, children ages 4-12 years for monocytes, children 1 month to 12 years for eosinophils and 0 days to 12 years for basophils

[4]Data from the 48 week visit of the Neverest 3 clinical trial; HIV-infected children were a mean (SD) age of 4.74 (0.94) years (20)

Table S10. Comparison of estimated mean (SD) cell percentages of lymphocytes in our study to one pediatric reference

Cell Type	All (N=179)	HIV-infected (N=119)	HIV-uninfected (N=60)	Reference[1]
$CD8^+$ T-cells	29.3 (7.1)	29.5 (6.8)	28.8 (7.7)	25 (18-35)
$CD4^+$ T-cells	34.7 (9.4)	33.9 (9.2)	36.2 (9.6)	37 (31-47)
Natural killer cells	14.7 (11.1)	15.8 (11.4)	12.3 (10.2)	9 (4-17)
B-cells	21.4 (6.8)	20.8 (7.1)	22.6 (6.0)	18 (13-27)

[1]Pediatric reference for children 6-12 years in Shearer *et al.* (35); reported is median and 10th to 90th percentile

Table S11. Estimated mean (SD) cell percentages of white blood cells and lymphocytes, by HIV status and age at ART start for HIV-infected children

Cell type, Mean (SD)	HIV-infected (N=119)	HIV-uninfected (N=60)	P[1]	HIV-infected, started ART <6 months (N=60)	HIV-infected, started ART 6-24 months (N=59)	P[2]
**White Blood Cells**						
$CD8^+$ T-cell percentage	16.3 (6.3)	14.5 (5.4)	0.055	16.3 (6.3)	16.4 (6.3)	0.92
$CD4^+$ T-cell percentage	18.6 (7.2)	18.2 (7.1)	0.71	17.9 (7.1)	19.3 (7.4)	0.31
Natural killer cell percentage	8.7 (6.8)	6.1 (5.3)	0.012	9.7 (7.5)	7.6 (6.0)	0.09
B-cell percentage	11.3 (4.8)	11.2 (4.1)	0.86	11.3 (5.4)	11.3 (4.1)	0.97
Monocyte percentage	6.9 (3.2)	7.6 (3.3)	0.18	6.9 (3.0)	6.8 (3.3)	0.76
Granulocyte percentage	38.3 (13.6)	42.5 (11.2)	0.039	37.9 (14.0)	38.7 (13.3)	0.73
**Lymphocytes**						
$CD8^+$ T-cell percentage	29.5 (6.8)	28.8 (7.7)	0.55	29.2 (7.1)	29.8 (6.5)	0.63
$CD4^+$ T-cell percentage	33.9 (9.2)	36.2 (9.6)	0.12	32.8 (9.7)	35.0 (8.7)	0.18
Natural killer cell percentage	15.8 (11.4)	12.3 (10.2)	0.047	17.5 (12.5)	14.1 (10.1)	0.11
B-cell percentage	20.8 (7.1)	22.6 (6.0)	0.08	20.5 (8.4)	21.0 (5.5)	0.71

[1]P-value represents result of t-test comparing HIV-infected to HIV-uninfected group
[2]P-value represents result of t-test comparing those who started ART <6 months to those who started ART 6-24 months

Table S12. Top 20 sites associated with sex in our study, sorted by smallest to largest p-value, and ranking relative to top sites (20 or 30) from 4 other studies of sex and DNA methylation

Top 20 sites associated with sex in our study and ranking								Ranking of top sites from other studies			
**Ranking**	**CpG**	**CHR**	**Gene**	**Beta (Boys)**	**Beta (Girls)**	**Δβ**	**Adj P**	**Inoshita**	**Spiers**	**Xu**	**Yousefi**
**1**	cg11643285	3	RFTN1	0.705	0.975	-0.271	2.23E-151		3	3	
**2**	cg03691818	12	KRT77	0.024	0.125	-0.101	5.55E-96	5	2	4	1
**3**	cg12691488	1		0.278	0.090	0.188	2.20E-95	1	1		
**4**	cg04946709	16	LOC644649	0.842	0.664	0.178	1.63E-77	8	6	2	13
**5**	cg03618918	1		0.816	0.676	0.14	1.02E-76	2		7	
**6**	cg02325951	14	FOXN3	0.764	0.655	0.109	1.03E-61		4		9
**7**	cg06710937	13		0.033	0.130	-0.097	2.40E-58	14	11	16	
**8**	cg21148594	14		0.802	0.679	0.123	5.23E-56		18		
**9**	cg12052203	11	B3GNT1	0.067	0.020	0.047	6.06E-54	13			
**10**	cg08906898	20	RBM39	0.973	0.931	0.042	4.45E-50				12
**11**	cg04858776	11		0.047	0.128	-0.081	7.93E-50			6	
**12**	cg20299935	17		0.728	0.861	-0.134	1.99E-48	12			
**13**	cg22345911	17	CSNK1D	0.024	0.047	-0.023	2.87E-45		8		17
**14**	cg23719534	15		0.872	0.960	-0.088	6.82E-42		9		
**15**	cg15817705	1		0.825	0.685	0.140	3.46E-41	16	7	5	
**16**	cg09513416	6		0.881	0.796	0.085	7.69E-40				
**17**	cg17743279	7	CDK6	0.015	0.026	-0.011	1.82E-39				3
**18**	cg24016844	1	C1orf103	0.045	0.027	0.018	3.19E-39		16		27
**19**	cg03608000	19	ZNF69	0.011	0.021	-0.010	4.11E-38			20	8
**20**	cg25304146	18	WBP11P1	0.630	0.526	0.104	7.45E-38	4		9	16
21	cg12900929	12	PRDM4	0.056	0.106	-0.050	2.36E-37				30
22	cg06152526	16	PLLP	0.825	0.735	0.090	3.69E-37				19
24	cg09971754	16	ANKRD11	0.043	0.020	0.023	4.05E-37		20		
25	cg06759085	2	NAB1	0.940	0.861	0.079	1.65E-36			17	
26	cg23814743	3	NICN1	0.211	0.263	-0.052	3.22E-34	15			
27	cg05100634	18	SMAD2	0.015	0.022	-0.007	2.33E-32				7
32	cg01906879	3	GBE1	0.019	0.032	-0.012	1.22E-29				18
33	cg26355737	13	TFDP1	0.960	0.905	0.055	1.96E-29			11	5
35	cg07628841	2	GPN1;CCDC121	0.361	0.423	-0.062	8.58E-28				22
40	cg22266749	4	COL25A1	0.107	0.063	0.045	3.40E-26	9			
43	cg25438440	3	CLDND1	0.015	0.022	-0.007	1.91E-24				25
46	cg26516287	7	SCIN	0.745	0.820	-0.074	6.35E-24			13	

48	cg16727519	1	H3F3A	0.544	0.488	0.057	7.42E-24			15	
49	cg23001456	17	KIAA0664; CLUH	0.025	0.037	-0.012	7.57E-24		12		23
52	cg02989351	2	YWHAQ	0.131	0.163	-0.032	3.00E-23				14
56	cg16169375	6	C6orf108	0.015	0.022	-0.007	1.23E-22			18	
57	cg12204423	4	PHF17	0.050	0.036	0.013	1.60E-22				15
59	cg12177922	1	HAX1	0.120	0.205	-0.085	2.52E-22	10			
63	cg26213873	1	CTTNBP2NL	0.028	0.041	-0.013	6.38E-22				24
68	cg06644124	1	ZNF281	0.265	0.319	-0.054	5.44E-20				21
69	cg04874129	16	SLC6A2	0.077	0.112	-0.035	6.29E-20		15		11
70	cg01225095	12	YARS2	0.038	0.054	-0.016	1.28E-19			14	
72	cg03687700	2	FKBP1B	0.171	0.233	-0.062	5.54E-19		5		
76	cg12614789	15		0.071	0.101	-0.030	1.18E-18		19		
101	cg11841231	2	PARD3B	0.957	0.927	0.030	2.73E-16		10		28
124	cg22227586	10	FAM35A	0.020	0.025	-0.005	1.06E-13			12	
192	cg03218192	17	AP2B1	0.250	0.332	-0.082	1.97E-11	17	13		
Not in top 1000	cg26921482	16	AMDHD2								2
Not in top 1000	cg07852945	9	TLE1					18			4
Not in top 1000	cg25568337	6	ARID1B					7		10	6
Not in top 1000	cg17612569	21	GABPA; ATP5J								10
Not in top 1000	cg04190002	22	SHANK3								20
Not in top 1000	cg07816873	12	ERC1								26
Not in top 1000	cg13323902	5	VTRNA1-1								29
Not in top 1000	cg25294185	11	RNASEH2C					20		1	
Not in top 1000	cg17232883	11						6		8	
Not in top 1000	cg20808136	15								19	
Not in top 1000	cg06642617	2	CCT7						14		
Not in top 1000	cg22921760	14	SALL2						17		
Not in top 1000	cg23256579	12	PRR4					19			
Not in top 1000	cg17238319	3	RFTN1					3			

- Spiers et al. 2015; Methylomic trajectories across human fetal brain development; Table 3 – The 20 top ranked autosomal sex differences in DNA methylation in the fetal brain
- Inoshita et al. 2015; Sex differences of leukocytes DNA methylation, adjusted for estimated cellular proportions; Table 1 – Top 20 autosomal CpG sites with significant sex differences
- Xu et al. 2014; Sex-biased methylome and transcriptome in human prefrontal cortex; Table 2 – Top 20 autosomal CpGs with sex-biased methylation
- Yousefi et al. 2015; Sex differences in DNA methylation assessed by 450 K in newborns; Table 3 – Results for the top 30 gene-annotated autosomal DMPs associated with sex in CHAMACOS newborns
